# Proteome Analysis and In Vitro Antiviral, Anticancer and Antioxidant Capacities of the Aqueous Extracts of *Lentinula edodes* and *Pleurotus ostreatus* Edible Mushrooms

**DOI:** 10.3390/molecules26154623

**Published:** 2021-07-30

**Authors:** Shaza M. Elhusseiny, Taghrid S. El-Mahdy, Mohamed F. Awad, Nooran S. Elleboudy, Mohamed M. S. Farag, Mahmoud A. Yassein, Khaled M. Aboshanab

**Affiliations:** 1 Department of Microbiology and Immunology, Faculty of Pharmacy, Ahram Canadian University (ACU), 4th Industrial Area, 6th of October City, Cairo 2566, Egypt; shaza.ahmed@acu.edu.eg (S.M.E.); taghrid.elmahdy@acu.edu.eg (T.S.E.-M.); 2 Department of Microbiology and Immunology, Faculty of Pharmacy, Helwan University, Cairo 11795, Egypt; 3 Department of Biology, College of Science, Taif University, Taif 11099, Saudi Arabia; m.fadl@tu.edu.sa; 4 Department of Microbiology and Immunology, Faculty of Pharmacy, Ain Shams University, Organization of African Unity Street, Cairo 11566, Egypt; nooran.elleboudy@pharma.asu.edu.eg (N.S.E.); mahmoud.yassien@pharma.asu.edu.eg (M.A.Y.); 5 Botany and Microbiology Department, Faculty of Science, Al-Azhar University, Cairo 11884, Egypt; mohamed.farag@azhar.edu.eg

**Keywords:** white rot fungus, *Pleurotus ostreatus*, *Lentinula edodes*, shiitake, antioxidant, antitumor, antiviral

## Abstract

In this study, we examined aqueous extracts of the edible mushrooms *Pleurotus ostreatus* (oyster mushroom) and *Lentinula edodes* (shiitake mushroom). Proteome analysis was conducted using LC-Triple TOF-MS and showed the expression of 753 proteins by *Pleurotus ostreatus*, and 432 proteins by *Lentinula edodes*. Bioactive peptides: Rab GDP dissociation inhibitor, superoxide dismutase, thioredoxin reductase, serine proteinase and lectin, were identified in both mushrooms. The extracts also included promising bioactive compounds including phenolics, flavonoids, vitamins and amino acids. The extracts showed promising antiviral activities, with a selectivity index (SI) of 4.5 for *Pleurotus ostreatus* against adenovirus (Ad7), and a slight activity for *Lentinula edodes* against herpes simplex-II (HSV-2). The extracts were not cytotoxic to normal human peripheral blood mononuclear cells (PBMCs). On the contrary, they showed moderate cytotoxicity against various cancer cell lines. Additionally, antioxidant activity was assessed using DPPH radical scavenging, ABTS radical cation scavenging and ORAC assays. The two extracts showed potential antioxidant activities, with the maximum activity seen for *Pleurotus ostreatus* (IC50 µg/mL) = 39.46 ± 1.27 for DPPH; 11.22 ± 1.81 for ABTS; and 21.40 ± 2.20 for ORAC assays. This study encourages the use of these mushrooms in medicine in the light of their low cytotoxicity on normal PBMCs vis à vis their antiviral, antitumor and antioxidant capabilities.

## 1. Introduction

Mushrooms have great prospects in medicine and nutraceutical production. In addition to having good organoleptic properties and high nutritional values, many mushrooms were reported to have a myriad of pharmacological activities [1,2,3]. Among the most widely cultivated mushrooms are *Lentinula edodes* (shiitake mushroom) and *Pleurotus ostreatus* (oyster mushroom). Shiitake comes in second to *Agaricus bisporus* as the most consumed edible mushroom worldwide [4]. This mushroom has promising antibacterial, antifungal, antiviral, hepatoprotective, antihyperglycemic and immunomodulatory effects [5,6,7]. It is rich in bioactive molecules, the most studied of which is “lentinan”, a polysaccharide with an effect against bacteria, viruses and tumors, in addition to “lentinacin”, which showed help in controlling dyslipidemia and hyperglycemia [8]. *Pleurotus ostreatus*, the most common species of the genus *Pleurotus* [9], also known as the oyster mushroom, is a wood decomposer and has a broad range of biological activities [10]. In comparison to other therapeutic mushrooms, oyster mushrooms are becoming more popular as health promoters [11]. The presence of a large number of nutritious components such as lectins, polysaccharides, vitamins and minerals in oyster mushrooms makes them able to possess potential anticancer, antioxidant, antidiabetic, antimicrobial and anti-hypercholesterolemic properties [12,13]. In comparison to other edible mushrooms, oyster and shiitake mushrooms have a brief growing period and can be harvested throughout the year [14]. As a result of their ease of cultivation and testing, high nutritional values and promising medicinal benefits, these mushrooms have tremendous potential in the food and pharmaceutical industries [4,15]. Indeed, higher Basidiomycetes can be promising multifunctional food sources.

Many bioactive compounds, including sugars, physiologically active proteins, un-saturated fatty acids, phenolics (phenolic acids and polyphenols), flavonoids, terpenoids, glycoproteins, polyketides, steroids and alkaloids, were found in these two mushrooms [16,17]. These compounds act solely or synergistically to bring about the broad pharmacological actions of these fungi. Shiitake and oyster mushrooms are reported to have antibacterial, antiviral, antihypertensive, immunomodulatory and antioxidant activities [1,18,19]. Seo et al. [20] summarized the mechanisms of the antiviral activity of mushroom biomolecules, indicating that the reduction in viral infection is mainly through interfering with the uptake of the virus into host cells, its replication and its protein and enzyme synthesis, in addition to stimulating the host immune response [21]. Antioxidants are important molecules in the face of reactive oxygen species which are behind many health problems. Since many red flags were raised against the use of synthetic antioxidants, the study of natural ones has become mandatory [22]. The antitumor effect of these mushroom products was related to biomolecules including glucans, ergosterol, proteoglucans and amino acids (arginine and glutamine) [23]. The postulated mechanism underlying this effect may include the stimulation of T-lymphocytes, suppression of neovascularization and induction of cancer cell death, in addition to triggering the immune response against cancer cells [24,25].

Proteomics has proven itself as one of the valuable tools in bioresearch, particularly agricultural research [26]. Lindequist et al. [27] were the first group to shed light on the importance of employing proteomics in edible mushroom research. They emphasized the need for using “omics” for the study of fungal bioactive molecules. Proteomics has been used scarcely in edible mushroom research vis à vis pathogenic fungi [28]. Accordingly, the aims of this study were to analyze the proteome of the two edible mushrooms, *Pleurotus ostreatus* and *Lentinula edodes*, and to investigate their potential antiviral, antitumor and antioxidant activities.

## 2. Results

### 2.1. Proteome Analysis

**Figure 1:**
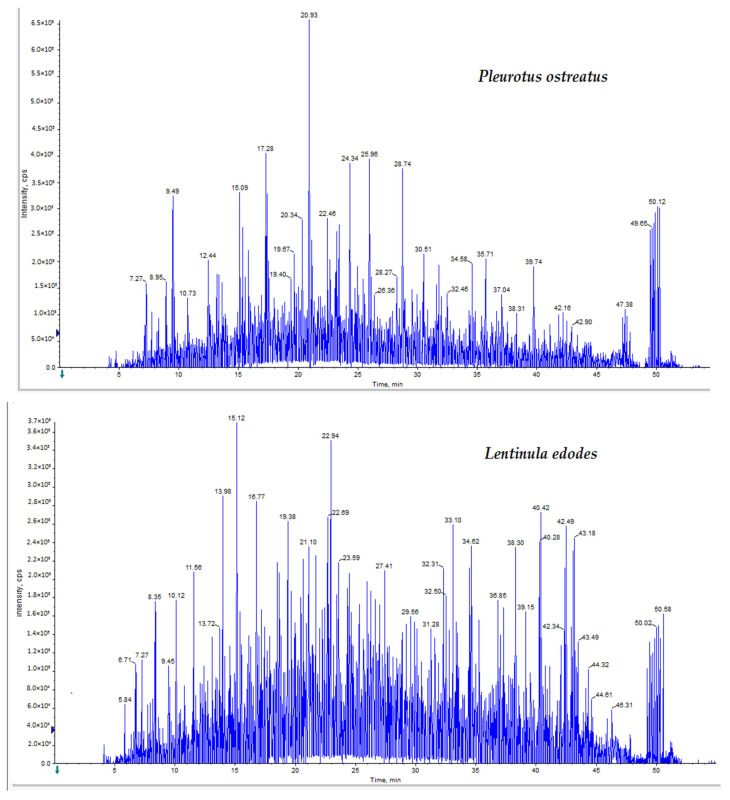
LC-Triple TOF-MS spectra showing the ion peaks of the mushroom proteins using Analyst (Sciex software).

Proteome analysis was conducted using LC-Triple TOF-MS analysis with a false discovery rate (FDR) of <5% and a “95% confidence of identification”. For *P. ostreatus*, a total of 753 proteins were identified, 34 of which were reversed hits. A total of 432 proteins were detected in *L. edodes*, 48 of which were reversed hits. The recorded accession numbers of the proteins are included in Tables S1 and S2 for *P. ostreatus* and *L. edodes*, respectively. In Tables S1 and S2, the MS-Triple TOF data were analyzed by ProteinPilot with the Paragon Algorithm. Proteins were identified with peptides that gave more than a 95% confidence of identification. Bioactive proteins: Rab GDP dissociation inhibitor, superoxide dismutase, thioredoxin reductase, serine proteinase and lectin, were expressed in both *P. ostreatus* and *L. edodes* extracts [29,30,31,32,33,34,35]. *P. ostreatus* also expressed ostreolysin and pleurotolysin, whereas latcripin and valosin-containing protein were expressed by *L. edodes*. LC-Triple TOF-MS spectra of the mushroom extracts analyzed by Analyst TF 1.7.1 (Sciex software) are shown in Figure 1.

### 2.2. Total Bioactive Compounds

#### Total Contents

In assessing the total contents, this included the assessment of the total carbohydrates, proteins, phenolics and flavonoids. The *P. ostreatus* extract contained almost triple the amount of carbohydrates and double the amount of proteins present in the *L. edodes* extract; nonetheless, more flavonoids were detected in the *L. edodes* extract than the *P. ostreatus* extract (Table 1).

### 2.3. Phenolic and Flavonoid Molecules

Both mushroom extracts contained catechin (detected at a retention time of 26.48 min). *P. ostreatus* additionally included kaempferol and apigenin (detected at retention times of 59.13 min and 59.56 min, respectively), whereas *L. edodes* also contained quercetin (retention time = 56.86 min; Table 2; Figure S1).

### 2.4. Vitamins

Water-soluble as well as fat-soluble vitamin contents were determined via HPLC (Table 3 and Figures S2 and S3). Both mushrooms are rich in vitamin C, with detectable amounts of vitamins B3, B6 and D. There was no significant difference between both mushrooms in the amounts of vitamins they possess. On the other hand, vitamins B1, B2, B9, A and E were undetected in both mushrooms.

### 2.5. Amino Acids Analysis

A Sykam Amino Acid Analyzer was used for the amino acid analysis. Total amino acid contents were 5.14 and 4.35 in *P. ostreatus* and *L. edodes*, respectively, as shown in Table 4, and the two mushrooms are rich in glutamic acid. While proline was detected in a considerable concentration (0.57 g/100 g protein) in the *P. ostreatus* extract, it was not detected in the *L. edodes* extract. Histidine and aspartic acid were detected in neither of the two mushrooms.

### 2.6. Biological Activities

#### 2.6.1. Antiviral Activity

Antiviral activity was evaluated against two viruses: adenovirus and herpes simplex-II. As shown in Table 5, the *P. ostreatus* extract showed an effective antiviral activity against adenovirus, with a selectivity index as high as 4.5. Promising antiviral effects were also recorded for the *P. ostreatus* extract against herpes simplex-II and for the *L. edodes* extract against both viruses. Figures S4 and S5 represent the dose–response curves.

#### 2.6.2. Cytotoxic Activity

##### Against Normal Human PBMCs

Minimal cytotoxicity (IC_50_ µg/mL; 66.41 ± 3.7 and 82.81 ± 2.72, for *P. ostreatus* and *L. edodes,* respectively) was recorded against normal human PBMCs.

##### Against Cancer Cell Lines

**Figure 2:**
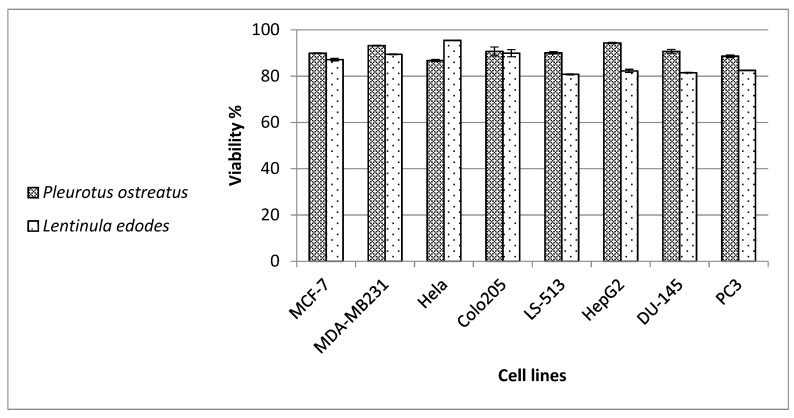
Cytotoxic effect of the mushroom extracts *Pleurotus ostreatus* and *Lentinula edodes* against cancer cell lines MCF-7, MDA-MBA-231, Hela, Colo-205, LS-513, HepG2, Du-145 and PC-3.

Varied cancer cell lines were used in this test including prostate cancer (DU-145 and PC3); hepatocellular carcinoma (HepG2); colorectal carcinoma (Colo-205); cecum carcinoma (LS-513); cervical cancer (HeLa); and breast adenocarcinoma (MDA-MB-231 and MCF-7). Doxorubicin was used as a positive control (caused a decrease in viability to 53% in HepG2; 44% in MDA-MB-231; 39% in PC3; 35% in DU-145; 29% in MCF-7; 25% in LS-513; 23% in HeLa; and 19% in Colo-205 cells). The results in Figure 2 show that the *L. edodes* and *P. ostreatus* extracts decreased the viability of the tested cancerous cell lines of LS-513, HepG2, DU-145 and PC-3 by approximately 20%.

##### Against Leukemia and Lymphoma Cell Lines

**Figure 3:**
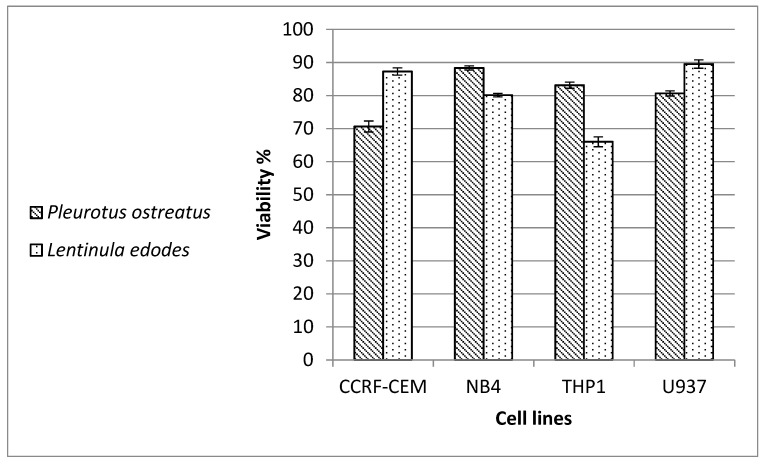
Cell viability of leukemic cells (CCRF-CEM, NB4 and THP1) and lymphoma U937 after treatment with *Pleurotus ostreatus* and *Lentinula edodes* extracts.

Mushroom extracts of *P. ostreatus* and *L. edodes* were tested for cytotoxicity against leukemia (CCR-CEM, NB-4, THP-1) and lymphoma (U937) cells. Doxorubicin was the positive standard, causing a decrease in cell viability to 29%, 25.5%, 20.3% and 19.1% in U937, NB4, CCRF-CEM and THP1 cells, respectively. The *L. edodes* extract decreased the viability of THP1 cells to 66.02%, whereas the *P. ostreatus* extract reduced the viability of CCRF-CEM cells to 70.64% (Figure 3).

#### 2.6.3. Antioxidant Activity

**Figure 4:**
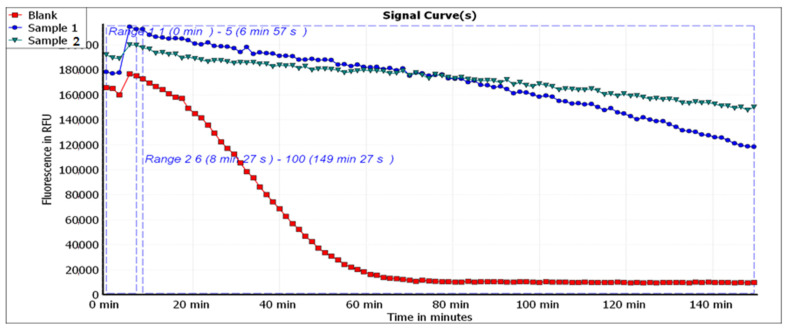
Signal curves indicating the decay of fluorescein upon applying the extracts: (1) *Pleurotus ostreatus* and (2) *Lentinula edodes*.

The results of the antioxidant effect studied by the three assessment methods, namely, DPPH radical scavenging, ABTS radical cation scavenging and ORAC assays, in comparison to the antioxidant standard, Trolox, are shown in Table 6 and Figure S6a,b. The results show that the *P. ostreatus* extract had a more potent antioxidant capacity than the *L. edodes* extract, as indicated by the three antioxidant assays used. Figure 4 shows the fluorescein signal decay induced by the extracts in the ORAC assay.

## 3. Discussion

White rot fungi have stirred the scientific community’s interest due to their medicinal properties, which include immune system modulation, hypoglycemic and antithrombotic activity and antihypertensive, anti-inflammatory, antimicrobial and antitumor properties, as well as the ability to lower blood cholesterol levels [24,36,37,38]. The two white rot fungi *P. ostreatus* and *L. edodes* are two of the most highly consumed edible mushrooms in many countries [39]. A multitude of bioactive proteins are produced by these two mushrooms, and proteome analysis is currently the most effective tool for protein profiling [40]. In the current study, proteome analysis showed the expression of bioactive proteins including the Rab GDP dissociation inhibitor, thioredoxin reductase, serine proteinase, superoxide dismutase and lectin in the two test mushrooms. The Rab GDP dissociation inhibitor (Rab GDI) regulates the function of Rab GTPases which play a pivotal role in membrane trafficking in tumor cells. Accordingly, using Rab GDI is a promising anticancer strategy [29].

Serine proteinase contributes to the antiviral activity of edible mushrooms [30,31]. Moreover, Yap et al. (2018) reported a strong selective cytotoxicity of serine proteinase in the face of a human breast adenocarcinoma cell line (MCF7) and suggested that the mechanism involves the collaborative effect of both extrinsic and intrinsic cell death mechanisms, in addition to the stimulation of caspase-8 and -9 and inhibition of Bcl-2 [41].

The intake of antioxidants is an auspicious prophylactic strategy against reactive oxygen species (ROS)-mediated pathophysiology [22]. Our results show the expression of enzymes of the antioxidant defense system by *Pleurotus ostreatus* and *Lentinula edodes.* These enzymes include superoxide dismutase, catalase and glutathione peroxidase, which counterbalance the production of ROS [32]. Superoxide dismutase starts by converting superoxides into hydrogen peroxide, which is, in turn, converted by catalase and glutathione peroxidase into water [33].

The expression of lectin was also detected in the two mushrooms. Many researchers reported the remarkably diverse biological profiles of lectin with a vast range of activities encompassing anti-inflammatory, antidepressant, anticonceptive and vasodilatory activities [34,35,42]. Lectins are carbohydrate-binding proteins with a variety of cellular functions including in vitro and in vivo suppression of tumor growth via the selective binding to tumor cell membranes or their receptors, resulting in the activation of protein kinases, or modulation of immune responses through interleukin production [43]. Additionally, mushroom lectins also have a role in triggering different cell death pathways, including apoptosis, necrosis and/or autophagy [42].

Our data show the expression of both ostreolysin and pleurotolysin in *Pleurotus ostreatus.* These are pore-forming proteins with highly selective anticancer activities [44,45]. Ostreolysin is a 15 kDa cytolytic protein with the ability to permeabilize erythrocytes and other cells at sub-micromolar concentrations. It acts via a colloid osmotic mechanism and induces the formation of wide membrane pores [46,47]. Nimri et al. produced recombinant pleurotolysin with potent antitumor activity against human and mouse colon tumor cells [48].

In *L. edodes*, valosin-containing protein and latcripin were expressed. Valosin-containing protein was previously reported to have antioxidant properties which could help in neuronal syndromes such as Alzheimer’s disease, lateral sclerosis and dementia [49,50,51]. Latcripin is a potential anticancer agent [50]. Riaz Ud Din et al. (2020) investigated the anticancer mechanism of latcripin against breast cancer cell lines. They reported its ability to induce cell death as well as autophagy, in addition to its inhibitory effect on migration and invasion [52].

A range of bioactive compounds were quantified in *P. ostreatus* and *L. edodes* extracts. TPNs of 19.37 ± 0.39 and 24.14 ± 1.01 mg/g of extract for *P. ostreatus* and *L. edodes*, respectively, were detected in the current study. Those values are higher than those recorded by Rahimah et al., who used the ammonium and Shinoda tests to quantify the total flavonoid content of *P. ostreatus* as 6.67 mg/g of extract [53]. Additionally, our results are higher than those found by Montibus et al., who assayed the TPC in *L. edodes* extracts using the Folin–Ciocalteu method and found 0.8–1.5% dry weight (dw) in the caps and 0.8–1.1% dw in the stipes [54].

Catechin was detected in both *P. ostreatus* and *L. edodes* extracts. Catechin is a plant secondary phenolic metabolite, with potent free radical scavenging properties [55]. The *L. edodes* extract was found to contain the flavonoid quercetin. The antioxidant effect of quercetin is mediated via the regulation of glutathione levels and an increase in the production of antioxidant enzymes including glutathione transferase and aldo-keto reductase [56]. In addition, quercetin is reported to have antitumor activity through interrupting the cell cycle and promoting apoptosis [57,58]. Lee et al. reported that quercetin causes cell arrest in the PC3, Du145 and U937 cancer cell lines [59]. Our results also reveal that kaempferol and apigenin were found in *P. ostreatus.* Kaempferol and apigenin have been proven to have antioxidant and anti-proliferative activities [60,61,62]. Glutamic acid was found to be the most abundant amino acid in both mushroom extracts. In the same vein, Chirinang et al. analyzed the amino acid content of *P. ostreatus* and *P. sajor-caju* and found that the glutamic acid content was the highest, followed by aspartic acid and then arginine [63].

*P. ostreatus and L. edodes* showed promising antiviral activities against adenovirus and *herpes simplex virus-II*, with the SI reaching 4.5 for the *P. ostreatus* extract against adenovirus. The SI was used to evaluate the efficacy and safety of the extracts as it estimates the window between the cytotoxic and antiviral activities. The higher the SI, the safer and more efficient the compound [64,65]. Related data were recently shown by Urbancikova et al., where pleuran (insoluble β-1,3/1,6-D-glucan isolated from *P. ostreatus*)-based supplements significantly shortened the duration of *herpes simplex virus-I* symptoms, with a lower severity of respiratory symptoms, in *herpes simplex virus-I*-positive patients than the placebo group, without significant side effects, proposing pleuran for possible future use in the treatment of acute *herpes simplex virus-I* [66].

The antiviral effect of mushrooms is mostly due to interfering with viral uptake, replication, enzyme activity and functioning peptides as well as potentiating the host immune system [67]. Seo and Choi (2021) proposed that the β-glucan in the polysaccharide fraction of *P. ostreatus* and *L. edodes* may be responsible for its antiherpetic effect through pre- and post-treatment effects [20]. The anti-HIV effect of the aqueous extracts of *P. ostreatus* and *L. edodes* may be caused by inhibition of the reverse transcriptase enzyme by ubiquitin-like protein and lentin, respectively [68,69].

The aqueous extracts of our mushrooms were shown to inhibit the viability of the tested cancer cell lines by approximately 20%. We also saw similar effects against leukemia and lymphoma cell lines, with a decrease in viability to 66% with the *L. edodes* extract against THP1 leukemia cells, and to 70.6% with the *P. ostreatus* extract against CCRF-CEM leukemia cells. Aqueous mycelial and fruit body extracts of *L. edodes* were previously reported to exert anti-proliferative and apoptotic actions on MCF-7 breast cancer cells [70]. The ethyl acetate fraction and β-glucan of *L. edodes* were mostly responsible for these actions [71,72]. In the same context, the *P. ostreatus* extract was able to show anti-proliferative activity toward MCF-7 and MDA-MB-231 breast adenocarcinoma cells [73], and the 6-linked glucans of the extract potentiated the natural killer cytotoxicity against breast and lung cancer cells [74]. Recently, Jakopovic et al. found that medicinal mushroom preparations consisting of 6 and 10 mushrooms including *L. edodes* and *P. ostreatus* exhibited significant anti-proliferative and pro-apoptotic effects on colorectal (HCT-116, SW620) tumor cell lines [75]. On the contrary, the authors noticed that the effect on a human fibroblast cell line (WI-38) was proliferative, showing the specificity of these mushroom preparations towards tumor cell lines. Similarly, we detected minimal cytotoxicity excreted by our mushrooms against normal human PBMCs. This finding also suggests the safe use of our mushrooms.

The cytotoxicity of mushrooms has been attributed to a wide range of molecules including α- and β-glucans, proteins, glycoproteins, fatty acids, nucleoside antagonists, terpenoids and phenolic compounds [76]. Abdalla et al. (2012) proposed that mushroom extracts suppressed breast cancer cell proliferation by inhibiting aromatase activity [77]. Imam et al. (2021) isolated an indole-3-lactic acid from *L. edodes* that inhibited the division of lung adenocarcinoma cells [78]. Yukawa et al. (2012) suggested that the direct apoptotic effect of *L. edodes* mycelia on HepG2 cells is through activation of the caspase-3 and -8 death receptor pathways [79]. Wu et al. (2011) studied the cytotoxic effect of the protein extract of *P. ostreatus* on human colorectal adenocarcinoma (SW480) cells and a human monocytic leukemia, THP-1 (cells), and reported generation of ROS, exhaustion of glutathione, alteration of mitochondrial membrane potential and disintegration of oligonucleosomal DNA, resulting in apoptosis of SW480 cells [80].

As antioxidants do not act through a single mechanism, evaluation of antioxidant capacity is usually conducted through more than one method [81]. In this study, three assessment methods were used, namely, DPPH, ABTS and ORAC assays. The SET (single electron transfer) antioxidant mechanism was evaluated through the DPPH assay, while the ABTS and ORAC assays were used for HAT (hydrogen atom transfer) reactions [81]. The ORAC assay provides more precise accurate estimates as it combines the inhibition time and the degree of inhibition in a single term [82]. While the phosphomolybdate assay is commonly used to express the total antioxidant capacity (TAC), all three methods used in this manuscript have been reported to evaluate the TAC. Munteanu and Apetrei (2021), in their review on the methods used in determining antioxidant activity, described the ABTS radical scavenging assay as a “simple and convenient method used to measure the total antioxidant capacity (TAC)” [83]. In their comparative study, Csepregi et al. (2016) compared four methods used for evaluation of the TAC, which were Trolox equivalent antioxidant capacity (TEAC), the ferric reducing antioxidant potential (FRAP), the 2,2-diphenyl-1-picrylhydrazyl (DPPH) radical scavenging assay and Folin–Ciocalteu reagent reactivity (FCR) [84]. In 2013, Ou et al. introduced ORAC as a novel method for evaluation of the TAC [85]. In the same vein, Rubio et al. (2016) classified the ORAC assay as being among the direct methods for the determination of the TAC, together with TEAC [86]. The results of the ABTS and ORAC assays show that the *P. ostreatus* and *L. edode* extracts have antioxidant capacities that are higher than those of the standard antioxidant, Trolox.

The antioxidant effect of the two mushrooms may be attributed to the presence of many bioactive components including flavonoids, phenolics, bioactive peptides and vitamin C [87]. Gaber et al. compared the ABTS, DPPH, ferric reducing antioxidant power (FRAP) and ORAC assays for assessing the antioxidant capacity of guava extracts and reported a positive correlation between antioxidant powers, as determined by the four methods, and the vitamin C content and TPN. On the other hand, they recorded a negative correlation with the carotenoid content [88]. DPPH (1,1-Diphenyl-2-picryl-hydrazyl) is a stable free radical which has an unpaired valence electron at one atom of the nitrogen bridge; thus, scavenging of the DPPH radical is the basis of the popular DPPH antioxidant assay [89]. The 2,2′-azino-bis (3-ethylbenzothiazoline-6-sulfonate) radical cation (ABTS) loses its blue color when reduced by an antioxidant, and the color alteration can be quantified spectrophotometrically [90]. The ORAC assay measures the oxidative degradation of the fluorescence probe by the free radicals which are interrupted by the antioxidant substance. Accordingly, the stronger the antioxidant capacity, the shorter the time needed to quench the fluorescence [91]. Additionally, since the tested extracts showed promising antioxidant effects when tested through the above-mentioned methods, it can be concluded that they act through free radical scavenging. However, the tested mushrooms contained considerable total flavonoid contents, and as flavonoids have been linked to the activation of the transcriptional factor Nrf2 (2017), this can be added as a putative mechanism [92].

## 4. Materials and Methods

### 4.1. Preparation of Mushroom Species and Sub-Culturing

Previously identified *P. ostreatus* and *L. edodes* mushrooms were obtained, as spawns and/or fruiting bodies, from the culture collection of the Agriculture Research Center, Cairo, Egypt [93,94]. Surgical blades were utilized to cut the fruit aseptically for sub-culturing from mushroom fruiting bodies. Inner mycelia were harvested using sterile needles and sub-cultured in sterile Potato Dextrose Agar (PDA) plates (Merck, Darmstadt, Germany) [95]. To sub-cultivate fungal spawns, sterile forceps were used to pick the seeds and aseptically transfer them to sterile PDA plates, which were then incubated at 28 °C for 7 days, and the fruiting bodies of each strain were washed separately. Each isolate (500 g) was left for 48 h at room temperature to air dry, ground manually in a kitchen grinder and stored at room temperature in a well-aerated area for future use.

### 4.2. Aqueous Extraction of Pleurotus Ostreatus and Lentinus Edodes

Each isolate’s dried fruit was ground into a 500 g powder using a domestic blender, then macerated three times in 7 L of distilled water at room temperature for three days, ultra-sonicated (ultrasonic cleaner, England, UK) and then filtered. The process of maceration and filtration was repeated until the liquid was exhausted. Ethanol was used to store the yield. After ethanol evaporation at 45 °C, a total of 500 mL of each isolate was freeze dried to yield approximately 20 g dry residue of each extract [96].

### 4.3. Proteomic Analysis

Proteomic analysis was conducted in the proteomics and metabolomics unit at Children’s Cancer Hospital Egypt 57,357 (CCHE), Cairo, Egypt. About 600 μL of 8 M urea in 500 mM Tris (pH 8.5) and 60 μL complete ultra-proteases (Roche, Mannheim, Germany) were added to each sample, and then samples were shaken vigorously and centrifuged at 10,000 RPM for 30 min. Supernatants were collected, and fractions (1 μg/10 μL) were injected using the NanoLC system. The mass spectrometry triple TOF system (Sciex TripleTOF 5600+, AB SCIEX, Concord) was coupled with liquid chromatography (LC) (3 um, ChromXP C18 CL, 120 A, 150 × 0.3 mm), consisting of an Eksigent nanoLC 400 autosampler attached to an Ekspert nanoLC425 pump, with a flow rate of 10 µL/min for 55 min, for each sample [97]. Data analysis was performed by ProteinPilot (version 5.0.1.0, 4895) and the Paragon Algorithm (version 5.0.1.0, 4874). Protein sequences were aligned against sequences in Swiss-Prot and the TrEMBL database (*Pleurotus* sp. containing 14,792 entries for *P. ostreatus*, and *Lentinula sp.* containing 12,603 entries for *L. edodes*) [98]. Commonly identified proteins between the mushroom species were illustrated using Venny2.1.0. BioinfoGP software [99] (Kingston upon Hull, England, UK).

### 4.4. Characterization of Bioactive Compounds

As previously reported, total soluble carbohydrate content was determined using the phenol sulfuric acid technique [100]. Data are presented as means ± standard deviation (SD), and experiments were performed in triplicate.

Total phenolic content (TPC) and total flavonoid content (TFC) were determined as reported by Ryan et al. [101]. Samples were prepared at a concentration of 20 mg/mL in water. Gallic acid (1 mg/mL stock solution) was prepared in methanol. Rutin (1 mg/mL stock solution) was prepared in methanol. Gallic acid standards and samples were pipetted in the wells of the plate in six replicates and measured at 630 nm. Each of the 10 rutin standards and samples in six replicates were measured at 420 nm.

HPLC analysis of total phenolics and flavonoids was conducted according to the method of Singh et al. [102]. Each of the samples and 10 different standard solutions were dissolved in methanol and filtered using 0.22 µm syringe filters, and then 100 µL of each sample and 10 µL of each standard were injected using an HPLC column, Waters 2690 Alliance HPLC system equipped with a Waters 996 photodiode array detector. Column C18 Inertsil ODS 3: 4.6 × 250 mm, 5 µm; mobile phase: buffer (0.1% phosphoric acid in water); methanol mode of elution was gradient with a flow rate of 1 mL/min at a wavelength of 280 nm.

For water-soluble vitamin analysis, the tested mushroom extracts (50 mg/mL), reference standards (10 mg in 10 mL 0.05 M sodium hydroxide) of each of the seven water-soluble vitamins (thiamine HCl, ascorbic acid, riboflavin, nicotinic acid, nicotinamide, pyridoxine HCl and folic acid) [103] and of the fat-soluble vitamins, solutions (50 mg/mL) of the three mushroom extracts and a standard solution in methanol of three fat vitamins: E, D3 and A, at 806.2, 114 and 400 IU/mL, respectively, were prepared. Solutions were then diluted to 100 µg/mL, filtered using a 0.22 µm syringe filter and 100 µL was injected onto an HPLC column, Waters 2690 Alliance HPLC system (Milford, CT, USA) equipped with a Waters 996 photodiode array detector. Column Inertsil ODS 3: 4.6 × 250 mm, 5 µm; mobile phase: buffer (0.85 g hexane sulphonic acid sodium salt in 1000 mL water and pH was adjusted to 3 with orthophosphoric acid); methanol elution was gradient at a flow rate of 1 mL/min at a wavelength of 210 nm [104].

### 4.5. Protein and Amino Acid Analysis

The composition of amino acids of the mushroom extracts was assessed using a Sykam Amino Acid Analyzer (Sykam GmbH, Germany) equipped with a solvent delivery system, S 2100 (quaternary pump with flow range 0.01 to 10.00 mL/min and maximum pressure up to 400 bar), an autosampler, S 5200, an amino acid reaction module, S4300 (with built-in dual-filter photometer at 570 nm), and a refrigerated reagent organizer, S4130. One gram of each mushroom extract was mixed with 5 mL hexane and allowed to macerate for 24 h, and then the mixture was filtered using Whatman no. 1 filter paper. The residue was placed in a test tube containing 10 mL 6N HCl and incubated in an oven at 110 °C for 24 h. After the incubation, filtration was conducted using Whatman no. 1 filter paper, followed by evaporation in a rotary evaporator. The residue was dissolved completely in 2 mL of dilution buffer (tri-sodium citrate dehydrate 0.06 M, citric acid 0.03 M, phenol 0.02 M, thiodiglycol 1.4%, HCL 32%), diluted 1000-fold in the same buffer and then loaded onto an ammonia filter column (LCA, K04/Na, 4.6 × 100 mm, Sykam GmbH, Eresing, Germany) equipped with an automatic amino acid analyzer (Sykam) [105].

### 4.6. Cell Cultures

Cervical cancer (HeLa), acute monocytic leukemia (Thp1), colorectal carcinoma (Colo-205) and cecum carcinoma (LS-513) cells were maintained in Roswell Park Memorial Institute (RPMI) medium (Lonza, Cologne, Germany) supplemented with 100 mg/mL of streptomycin, 100 units/mL of penicillin and 10% of heat-inactivated fetal bovine serum. Other cell lines: human leukemia (CCRF-CEM); acute promyelocytic leukemia (NB-4); human lymphoma (U937); prostate cancer (DU-145 and PC3); hepatocellular carcinoma (HepG2); breast adenocarcinoma (MCF-7 and MDA-MB-231); Vero; and Hep-2 cells, were sub-cultured on Dulbecco’s Modified Eagle Medium (DMEM; Invitrogen, California, USA), and incubation was conducted at 37 °C in a humidified atmosphere with 5% CO_2_. The cell lines were obtained from Nawah Scientific Inc. (Mokatam, Cairo, Egypt), while primary peripheral blood mononuclear cells (PBMCs) and normal human (ATCC^®^ PCS-800-011™) cells were purchased from Vascera, Cairo, Egypt. This work was approved by the Ethical Research Committee of the Faculty of Pharmacy, Ain Shams University (Approval No. 294).

### 4.7. Antiviral Activity

Antiviral activity was tested against *adenovirus type 7* (Ad7) and *herpes simplex virus type-II* (HSV-2) (Nawah Scientific, Mokattam, Egypt). Antiviral activity was assessed as previously described [106]. Vero and Hep-2 cells were seeded into a 96-well culture plate at a density of 2 × 10^4^ cells/well one day before infection. The cell culture medium (Section 4.6) was removed the next day, and phosphate-buffered saline (PBS) was used to wash the cells. The infectivity of human *adenovirus* and *herpes simplex virus type-II* was determined using the sulforhodamine B (SRB) method, which monitored cytopathic effects (CPE) and calculated the percentage of cell viability [107]. The antiviral activity was calculated based on the mushroom extracts’ ability to inhibit the viral CPEs, where the 50% cytotoxic concentration (CC_50_) and the 50% inhibitory concentration (IC_50_) were determined using GraphPad PRISM software (Graph-Pad Software, San Diego, CA, USA). The selectivity index (SI) was calculated as stated by Doğan et al. [108] according to the equation

Selectivity Index (SI) = CC_50_/IC_50_ (1)

### 4.8. Cytotoxicity against PBMCs Using MTT Assay

Cytotoxicity was assessed using the MTT assay [109]. Aliquots (100 µL) of cell suspensions (cell density 1.2–1.8 × 10^4^ cells/well) were placed in 96-well plates, complete medium was added and cells were incubated for 24 h. Then, cells were treated with serial dilutions of mushroom extracts for 48 h. MTT solution (5 mg/mL) in PBS was added to wells and incubated for 4 h, and absorbance at 570 nm was read using a microplate reader. Untreated cells were used as negative controls, and cells treated with doxorubicin (Sigma-Aldrich, Darmstadt, Germany) served as positive control.

### 4.9. Evaluation of Cytotoxic Activity Using SRB against Cancer Cell Lines

Cytotoxicity against prostate cancer (DU-145 and PC3); hepatocellular carcinoma (HepG2); colorectal carcinoma (Colo-205); cecum carcinoma (LS-513); cervical cancer (HeLa); and breast adenocarcinoma (MDA-MB-231 and MCF-7) cell lines was evaluated using sulforhodamine B (SRB) colorimetric assay [110]. Cell suspensions in complete media (4.6) were incubated in 96-well plates for 24 h. Then, various concentrations (0 to 100 μg/mL in complete media) of mushroom extracts were added and incubated for 72 h. Untreated cells were used as negative controls, and cells treated with doxorubicin served as positive control.

### 4.10. The Viability of Leukemia and Lymphoma Cell Lines Using WST-1 Assay

Cytotoxicity against human leukemia (CCRF-CEM), acute promyelocytic leukemia (NB4), acute monocytic leukemia (THP1) and human lymphoma (U937) was determined by water-soluble tetrazolium salt (WST-1) assay using Abcam^®^ kit (ab155902 WST-1 Cell Proliferation Reagent, United Kingdom). Cell suspensions were incubated for 24 h and then treated with different concentrations (0 to 100 μg/mL in complete media) of mushroom extracts for 48 h. Cells were then treated with 10 μL WST-1 reagent for 1 h, and A_450_ was read using a microplate reader [111]. Untreated cells were used as negative controls and cells treated with doxorubicin (Sigma-Aldrich, Germany) served as positive control.

### 4.11. Antioxidant Activity

#### 4.11.1. DPPH Radical Scavenging Activity

DPPH radical scavenging assay was carried out according to the method of Lu et al. [112]. The resulting reduction in DPPH color was measured at 540 nm. Antioxidant activity was expressed as the inhibition percentage with reference to the calibration curve (R^2^ = 0.9903). Data are represented as means ± SD according to Equation (2):

(Antioxidant activity% = [(abs-blank–abs-sample)/abs-blank] × 100)(2)

where abs-blank and abs-sample refer to the absorbance of the blank and sample, respectively. The sample concentration required to inhibit half of the free radicals (IC50) was estimated.

#### 4.11.2. ABTS (2,2′-Azinobis-(3-ethylbenzthiazolin-6-sulfonic Acid)) Radical Cation Scavenging Activity

The assay was carried out as previously described [113]. Mushroom extracts (10 µL of 0 to 100 μg/mL solutions) were mixed with 190 µL of freshly prepared ABTS (Sigma-Aldrich, Germany) in 96-well plates and incubated in the dark at room temperature for 2 h. Each sample was tested in four replicates, and absorbance was measured at 734 nm. Antioxidant activity was expressed as percentage of inhibition (Equation (1)) with reference to the calibration curve (R^2^ = 0.9948).

#### 4.11.3. Oxygen Radical Absorbance Capacity (ORAC) Assay

The analysis was carried out as previously determined [62], with minor modifications. Briefly, 12.5 µL of the mushroom extracts, in triplicate, were incubated with 75 µL of fluorescein (10 nm) for 30 min at 37 °C. Fluorescence measurement (485 EX, 520 EM, nm) was carried out for three cycles (cycle time = 90 s). Afterwards, 12.5 µL of freshly prepared 240 mm solution of 2,2′-Azobis 2-amidinopropane dihydrochloride (AAPH) (Abcam, Cambridge, UK) was added to each well immediately and fluorescence measurement was continued for 2.5 h (85 cycles, each 90 s). The assay measures the loss of fluorescein fluorescence over time as a result of peroxyl-radical formation by the breakdown of AAPH. Trolox (6-hydroxy-2,5,7,8-tetramethylchroman-2-carboxylic acid), a water-soluble vitamin E analog, served as a positive control inhibiting fluorescein decay in a dose-dependent manner. Antioxidant activity was expressed as percentage of inhibition (Equation (1)) with reference to the calibration curve (R^2^ = 0.9957).

### 4.12. Statistical Analysis

Triplicate chemical assays were conducted, and the results were expressed as mean ± standard deviations. Statistical analyses were performed by one-way ANOVA. The significance of differences between means was evaluated with the Tukey–Kramer multiple comparisons test. A *p*-value of ≤0.05 was considered statistically significant. Statistical evaluation was determined using GraphPad PRISM software (Graph-Pad Software, San Diego, CA, USA).

## 5. Conclusions

The mushrooms *P. ostreatus* and *L. edodes* contain a wide range of bioactive compounds. Proteome analysis showed the expression of multiple bioactive molecules with numerous biological activities. Aqueous extracts of the mushrooms have promising antiviral activities against *adenovirus type 7* and *herpes simplex virus type-II*. CPEs were detected against cancer cell lines but not against normal human PBMCs. The present study encourages the use of these mushrooms for pharmacological purposes in the light of their low cytotoxicity on normal PBMCs, in addition to their antiviral, antitumor and antioxidant capabilities.

## Figures and Tables

**Table 1:** Total bioactive compounds in *Pleurotus ostreatus* and *Lentinula edodes*.

Mushroom	Total Carbohydrate Content (mg/g Extract)	Total Phenolic Content (mg/g Extract)	Total Flavonoid Content (mg/g Extract)	Total Protein Content (mg/g Extract)
*Pleurotus ostreatus*	76.56 ± 2.52	19.37 ± 0.39	2.71 ± 0.06	0.1 ± 0.005
*Lentinula edodes*	26.51 ± 1.26	24.14 ± 1.01	5.79 ± 0.18	0.04 ± 0.008

All results are expressed as mean ± SD (*n* = 6).

**Table 2:** Phenolic and flavonoid content molecules in *Pleurotus ostreatus* and *Lentinula edodes* extracts.

	Gallic Acid	Catechin	Chlorogenic Acid	Caffeic Acid	Rutin	Ellagic Acid	Hesperidin	Quercetin	Kaempferol	Apigenin
*Pleurotus ostreatus*	ND *	Detected	ND *	ND *	ND *	ND *	ND *	ND *	Detected	Detected
*Lentinula edodes*	ND *	Detected	ND *	ND *	ND *	ND *	ND *	Detected	ND *	ND *

ND *: not detected.

**Table 3:** Water-and fat-soluble vitamin contents of *Pleurotus ostreatus* and *Lentinula edodes* extracts.

	Ascorbic Acid (Vit. C) (mg/100 g)	Nicotinic Acid (µg/100 g)	Nicotinamide (µg/100 g)	Pyridoxine (Vit. B6) (µg/100 g)	Folic Acid (Vit. B9) (µg/100 g)	Thiamine (Vit. B1) (µg/100 g)	Riboflavin (Vit. B2) (µg/100 g)
*Pleurotus ostreatus*	2.40 ± 0.29	0.31 ± 0.02	0.04 ± 0.03	0.25 ± 0.17	ND *	ND *	ND *
*Lentinula edodes*	1.95 ± 0.29	0.20 ± 0.03	0.02 ± 0.01	0.27 ± 0.22	ND *	ND *	ND *
		**Retinol (Vit. A) (µg/100 g)**	**Cholecalciferol (Vit. D) (µg/100 g)**		**Tocopherol (Vit. E) (mg/100 g)**		
*Pleurotus ostreatus*		ND *	0.06 ± 0.02		ND *		
*Lentinula edodes*		ND *	0.03 ± 0.01		ND *		

Each value is presented as the mean ± standard deviation (*n* = 3). ND *: not detected.

**Table 4:** Amino acid composition of *Pleurotus ostreatus* and *Lentinula edodes* extracts.

Amino Acid Content (g/ 100 g Protein)		
Amino Acids	*Pleurotus ostreatus*	*Lentinula edodes*
Aspartic acid	ND *	ND *
Threonine	0.278	0.279
Serine	0.387	0.369
Glutamic acid	1.178	1.071
Proline	0.568	ND *
Glycine	0.35	0.298
Alanine	0.462	0.399
Cystine	0.208	0.196
Valine	0.239	0.223
Methionine	0.281	0.263
Isoleucine	0.128	0.109
Leucine	0.376	0.332
Tyrosine	0.168	0.136
Phenylalanine	0.195	0.173
Histidine	ND *	ND *
Lysine	0.317	0.283
Arginine	0.276	0.216
Total	5.41	4.35

Each value is presented as the mean ± standard deviation (*n* = 3). ND *: not detected.

**Table 5:** Antiviral effect of *Pleurotus ostreatus* and *Lentinula edodes* extracts.

Mushroom	Virus	CC_50_ (µg/mL)	IC_50_ (µg/mL)	SI
*Pleurotus ostreatus*	*Adenovirus*	63.45	13.80	4.5
	*Herpes simplex virus-II*	23.69	11.81	2.0
*Lentinula edodes*	*Adenovirus*	34.83	12.82	2.7
	*Herpes simplex virus-II*	27.32	11.06	2.5

CC50, half-maximal cytotoxic concentration; IC50, half-maximal inhibitory concentration; SI, selectivity index = CC50/IC50.

**Table 6:** IC50 values of *Pleurotus ostreatus* and *Lentinula edodes* in the radical scavenging, ABTS radical cation scavenging and ORAC assays.

	IC_50_ (µg/mL)		
Mushroom	DPPH Radical Scavenging	ABTS Radical Cation Scavenging	ORAC Assay
*Pleurotus ostreatus*	39.46 ± 1.27 ^#^	11.22 ± 1.81 ^#^	21.40 ± 2.20 ^#^
*Lentinula edodes*	48.30 ± 1.85 ^#^	15.92 ± 1.30 ^#^	30.10 ± 2.11 ^#^
*Trolox*	24.00 ± 0.87	40.00 ± 0.03	55.51 ± 0.06

All results are expressed as mean ± SD (*n* = 6). ^#^ Significantly different from Trolox at (*p* ≤ 0.05).

## Data Availability

All data are included within the manuscript and supplementary file.

## Supplementary Materials

The following are available online. Figure S1: HPLC chromatogram (λ 280 nm) of the elution profile of the mushroom extracts of (A) *Pleurotus ostreatus* and (B) *Lentinula edodes* and pure phenolic and flavonoid standards: (a) mixture (gallic acid, catechin, chlorogenic acid, caffeic acid, hesperidin, rutin, ellagic acid, quercetin, kaempferol and apigenin), Figure S2: HPLC chromatogram of water-soluble vitamins of the standard mixture and the mushroom isolates of (A) *Pleurotus ostreatus* and (B) *Lentinula edodes,* Figure S3: HPLC chromatogram of fat-soluble vitamins of the standard mixture and the two mushroom isolates of (A) *Pleurotus ostreatus* and (B) *Lentinula edodes,* Figure S4: Cytotoxicity concentration (CC50) and 50% inhibitory concentration (IC50) on hep 2 cells and Adv7, Figure S5: Cytotoxicity concentration (CC50) and 50% inhibitory concentration (IC50) on Vero cells and HSV 2, Figure S6: Antioxidant effect of Trolox on the decay of fluorescein in ORAC assay. (A) Blank-corrected linear regression curve of Trolox. (B) Signal curves of different Trolox concentrations and blank indicating the decay of fluorescein with different concentrations of Trolox, Table S1: Protein identification of *Pleurotus ostreatus* by Uniprot database, Table S2: Protein identification of *Lentinula edodes* by Uniprot database.

## Sample Availability

Samples of the compounds are not available from the authors.
