# Supplementary material for: Proteome Analysis and In Vitro Antiviral, Anticancer and Antioxidant Capacities of the Aqueous Extracts of Lentinula edodes and Pleurotus ostreatus Edible Mushrooms
Source: Molecules. 2021 Jul 30;26(15):4623. doi: 10.3390/molecules26154623 (PMC8348442; doi:10.3390/molecules26154623)
Supplement: Supplementary file 1 [file molecules-26-04623-s001.zip › molecules-1301066-supplementary.pdf]

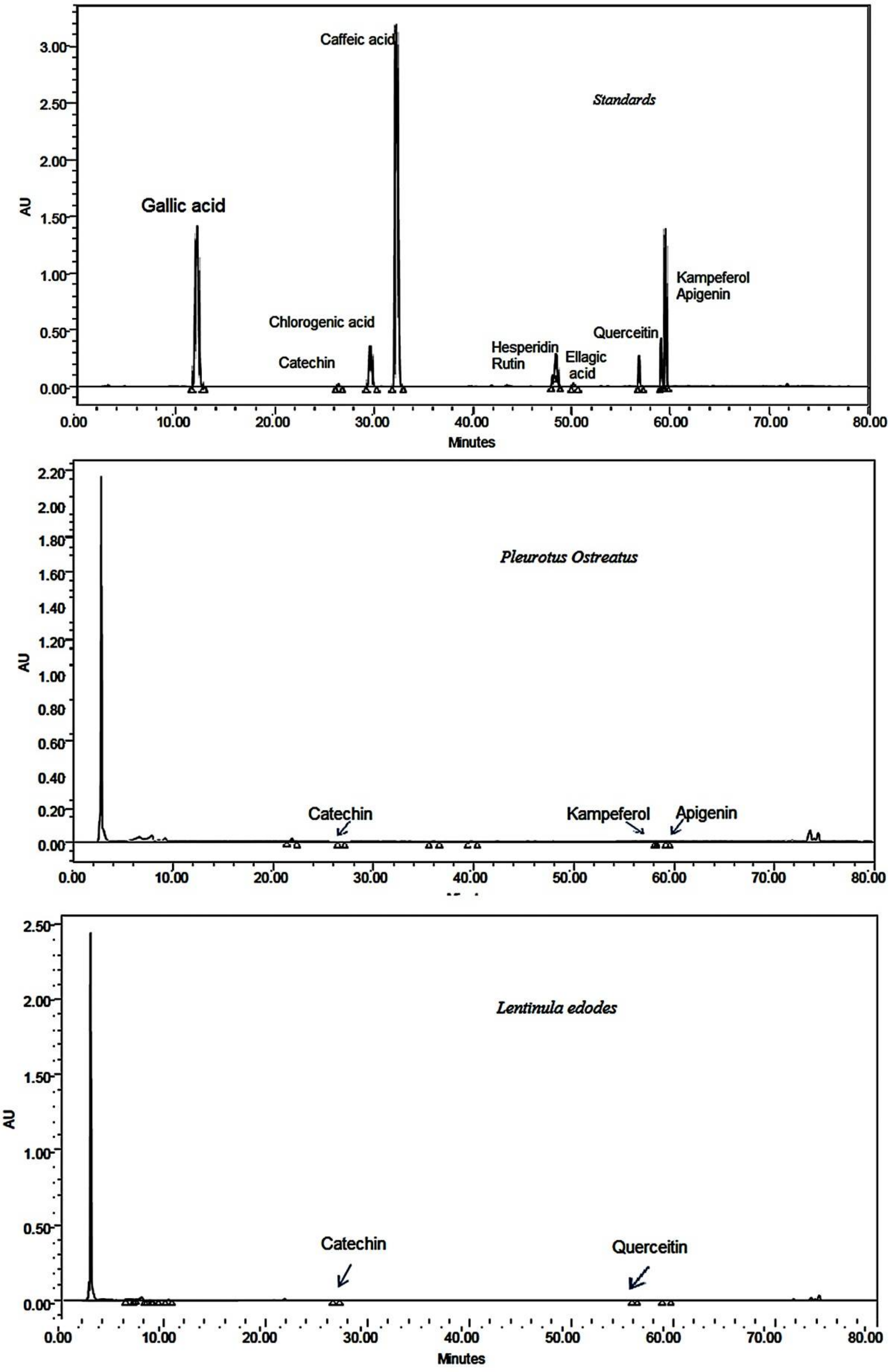
Caffeic acid
Standards
Gallic acid
Chlorogenic acid
Catechin
Hesperidin
Rutin
Ellagic acid
Quercetin
Kampeferol
Apigenin
AU
Minutes
Pleurotus Ostreatus
Catechin
Kampeferol
Apigenin
AU
Lentinula edodes
Catechin
Querceitin
AU
Minutes

Figure S1: HPLC chromatogram (λ 280nm)elution profile of the mushroom extracts *A) Pleurotus ostreatus and (B) Lentinula edodes* and pure phenolic and flavonoids standards (a) mixture (Gallic acid, Catechin, Chlorogenic acid, Caffeic acid , Hespiridin, Rutin, Ellagic acid, Quercetin, Kampeferol and Apigenin)

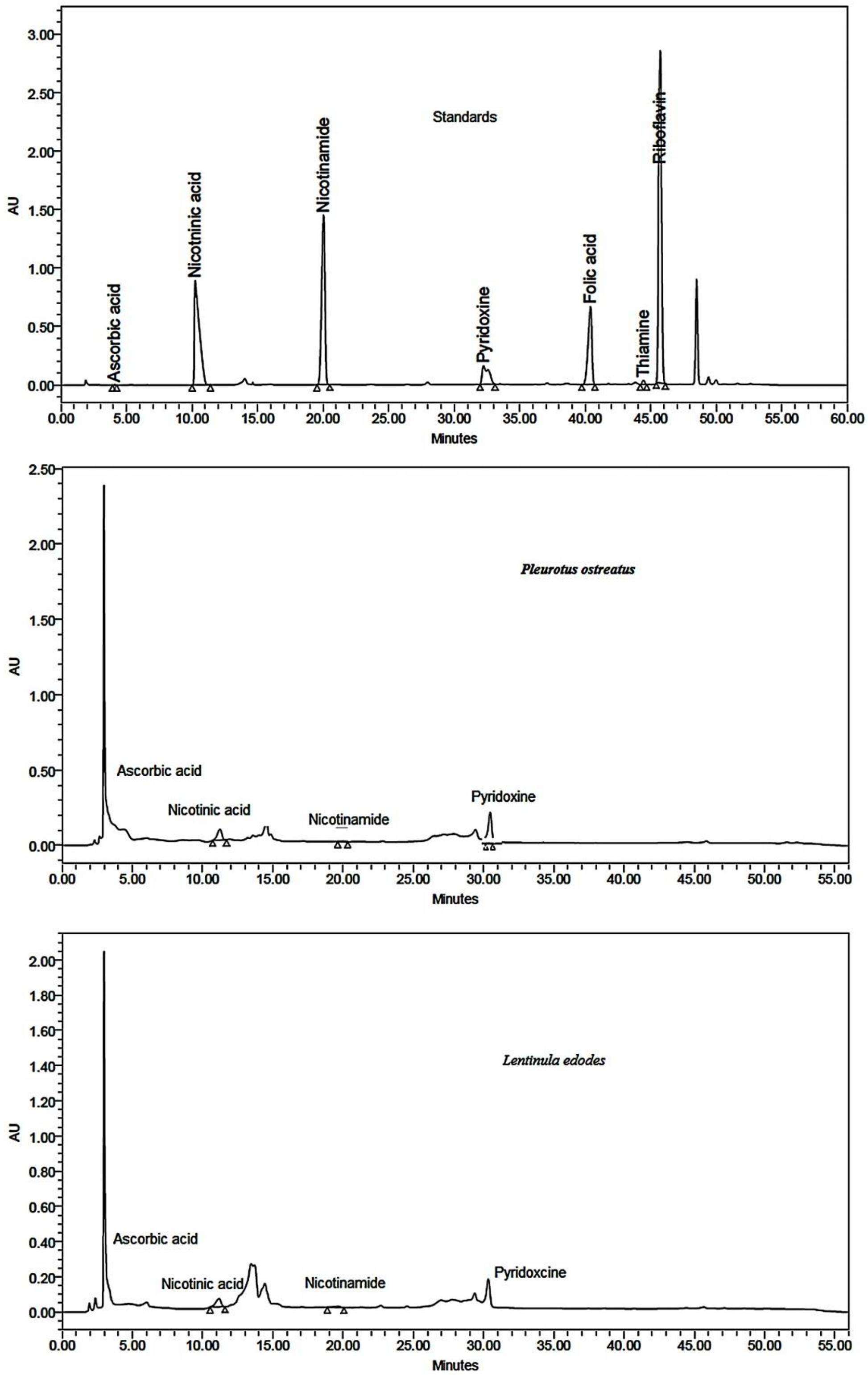
Standards
AU
Minutes
Ascorbic acid
Nicotninic acid
Nicotinamide
Pyridoxine
Folic acid
Thiamine
Riboflavin
0.00
0.50
1.00
1.50
2.00
2.50
3.00
0.00 5.00 10.00 15.00 20.00 25.00 30.00 35.00 40.00 45.00 50.00 55.00 60.00
Pleurotus ostreatus
Ascorbic acid
Nicotinic acid
Nicotinamide
Pyridoxine
AU
Minutes
0.00
0.50
1.00
1.50
2.00
2.50
0.00 5.00 10.00 15.00 20.00 25.00 30.00 35.00 40.00 45.00 50.00 55.00
Lentinula edodes
Ascorbic acid
Nicotinic acid
Nicotinamide
Pyridoxcine
AU
Minutes
0.00
0.20
0.40
0.60
0.80
1.00
1.20
1.40
1.60
1.80
2.00
0.00 5.00 10.00 15.00 20.00 25.00 30.00 35.00 40.00 45.00 50.00 55.00

Figure S2: HPLC chromatogram for water-soluble vitamins of the standard mixture and the mushroom isolates *A) Pleurotus ostreatus and (B) Lentinula edodes*

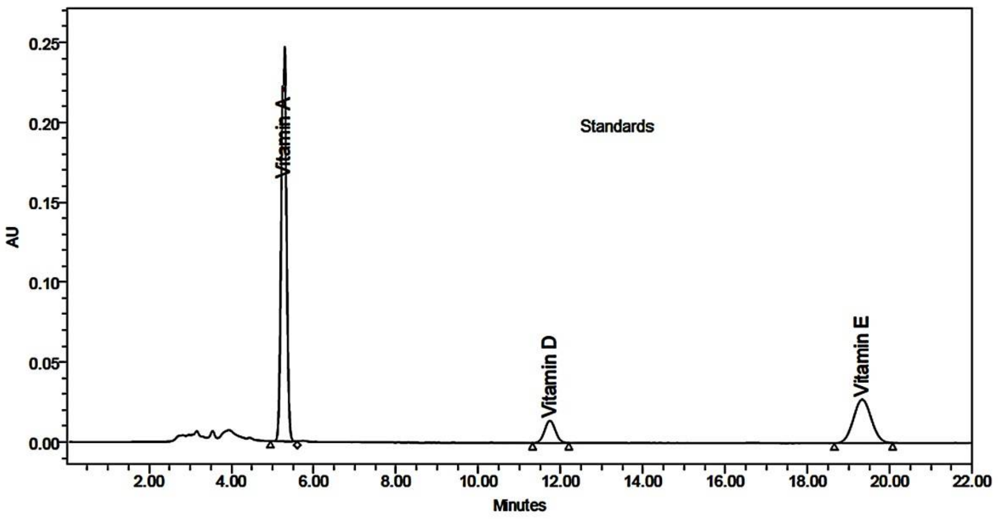
Standards
Vitamin A
Vitamin D
Vitamin E
AU
Minutes

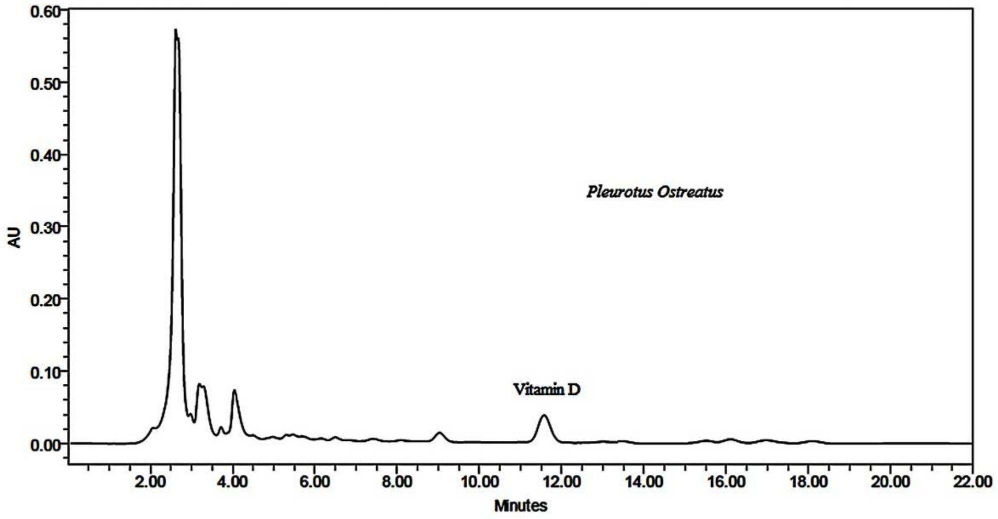
Pleurotus Ostreatus
Vitamin D
AU
Minutes

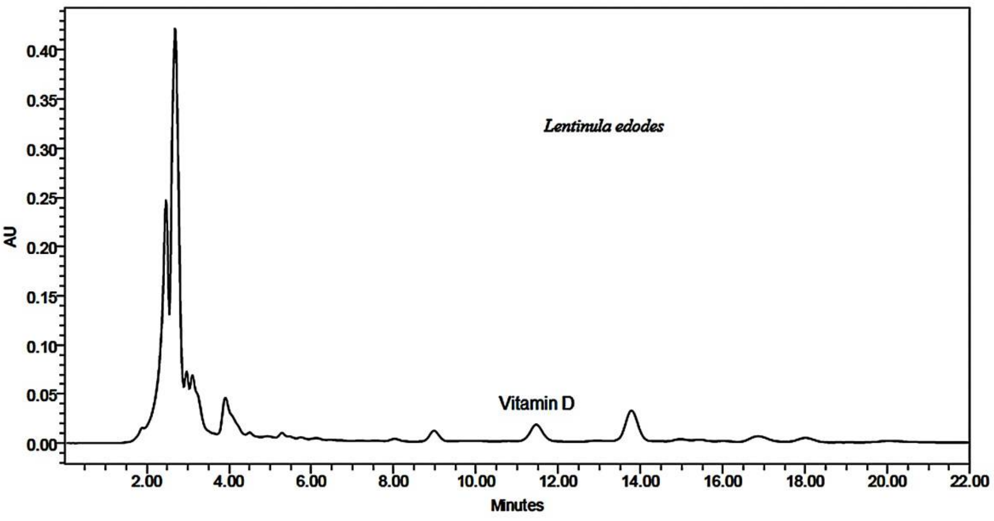
Lentinula edodes
Vitamin D
AU
Minutes

Figure S3: HPLC chromatogram of fat-soluble vitamins of the standard mixture, and the two mushroom isolates A) *Pleurotus ostreatus* and (B) *Lentinula edodes*

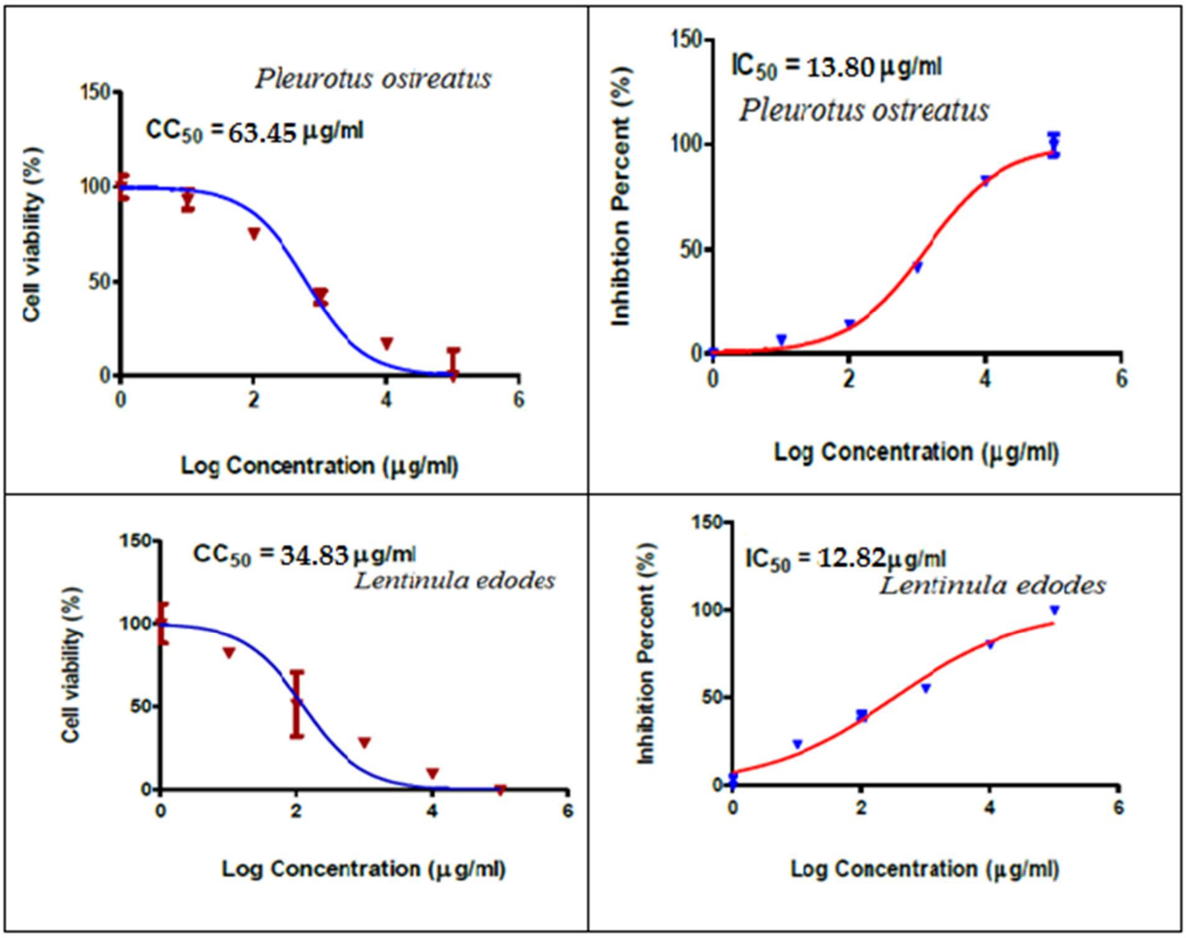

Figure S4: Cytotoxicity concentration (CC50) and 50% inhibitory concentration (IC50) on hep 2 cells and Adv7

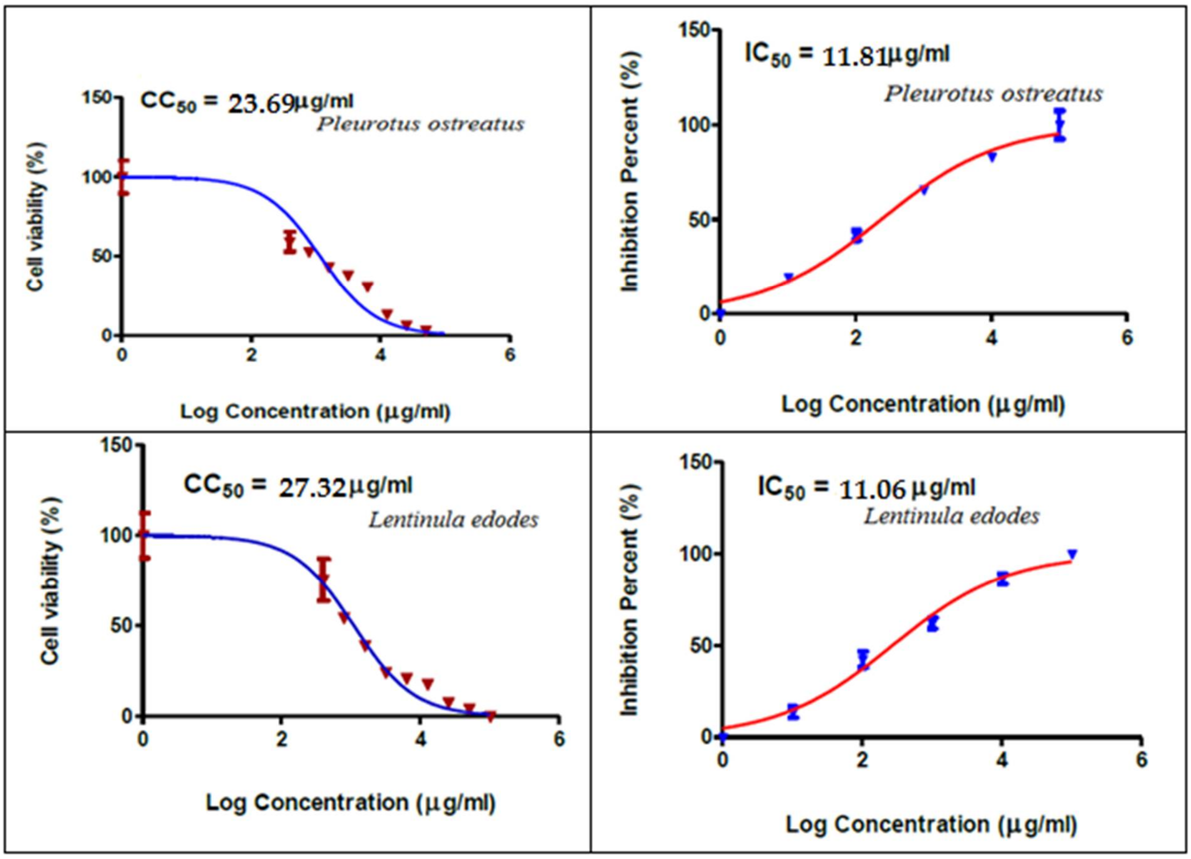

Figure S5: Cytotoxicity concentration (CC50) and 50% inhibitory concentration (IC50) on Vero cells and HSV 2

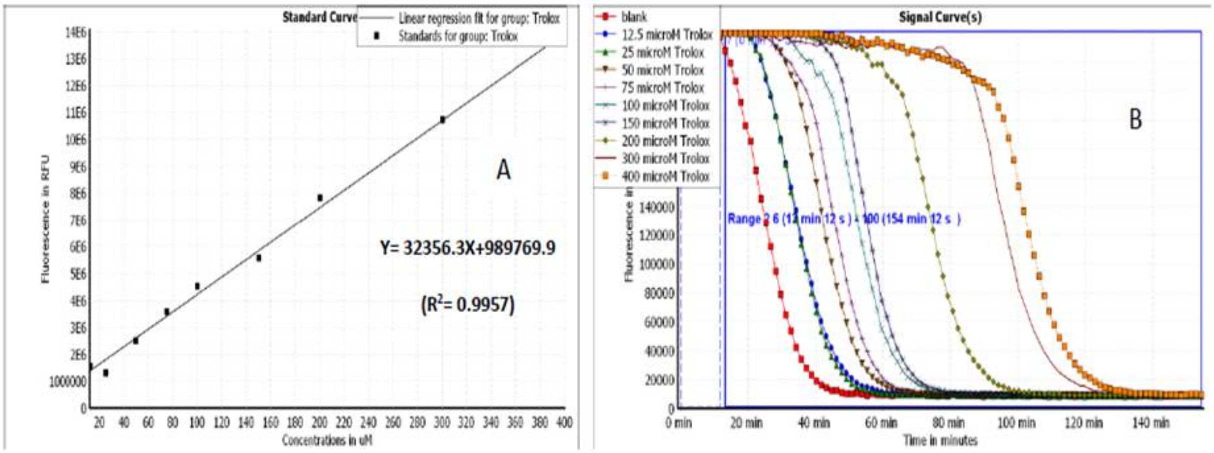

Figure S6: Antioxidant effect of Trolox on the decay of fluorescein in ORAC assay. (A) Blank corrected linear regression curve of Trolox. (B) Signal curves of different Trolox concentrations and blank indicating the decay of fluorescein with different concentrations of Trolox

Table S1: Protein identification of *Pleurotus ostreatus* by Uniprot database

N	Accession	Protein Name/Species	Peptides
1	tr|A0A067NWS5|A0A067NWS5_PLEOS	Uncharacterized protein OS=Pleurotus ostreatus PC15 OX=1137138 GN=PLEOSDRAFT_1082642 PE=4 SV=1	87
2	tr|A0A067NHC4|A0A067NHC4_PLEOS	Uncharacterized protein OS=Pleurotus ostreatus PC15 OX=1137138 GN=PLEOSDRAFT_1055831 PE=4 SV=1	45
3	tr|A0A067N8H6|A0A067N8H6_PLEOS	Uncharacterized protein OS=Pleurotus ostreatus PC15 OX=1137138 GN=PLEOSDRAFT_1090690 PE=4 SV=1	35
4	tr|A0A067NUP1|A0A067NUP1_PLEOS	Elongation factor 1-alpha OS=Pleurotus ostreatus PC15 OX=1137138 GN=PLEOSDRAFT_1070256 PE=3 SV=1	60
5	tr|A0A067NW08|A0A067NW08_PLEOS	Elongation factor 2 OS=Pleurotus ostreatus PC15 OX=1137138 GN=PLEOSDRAFT_1088686 PE=3 SV=1	46
6	tr|A0A067P9U4|A0A067P9U4_PLEOS	HATPase_c domain-containing protein OS=Pleurotus ostreatus PC15 OX=1137138 GN=PLEOSDRAFT_1052040 PE=3 SV=1	32
7	tr|A0A067NYT5|A0A067NYT5_PLEOS	Uncharacterized protein OS=Pleurotus ostreatus PC15 OX=1137138 GN=PLEOSDRAFT_1087719 PE=3 SV=1	35
8	tr|A0A067P0J4|A0A067P0J4_PLEOS	5-methyltetrahydropteroyltriglutamate--homocysteine S-methyltransferase OS=Pleurotus ostreatus PC15 OX=1137138 GN=PLEOSDRAFT_1064157 PE=3 SV=1	42
9	tr|A0A067P303|A0A067P303_PLEOS	6-phosphogluconate dehydrogenase, decarboxylating OS=Pleurotus ostreatus PC15 OX=1137138 GN=PLEOSDRAFT_1095873 PE=3 SV=1	32
10	tr|A0A067NLR3|A0A067NLR3_PLEOS	ATP-citrate synthase OS=Pleurotus ostreatus PC15 OX=1137138 GN=PLEOSDRAFT_1058547 PE=3 SV=1	20

11	tr|A0A067P9S7|A0A067P9S7_PLEOS	Glycosyltransferase family 4 protein OS=Pleurotus ostreatus PC15 OX=1137138 GN=PLEOSDRAFT_1060842 PE=4 SV=1	30
12	tr|A0A482GPW1|A0A482GPW1_PLEOS	Catalase OS=Pleurotus ostreatus OX=5322 PE=2 SV=1	26
12	tr|A0A2H4UZK7|A0A2H4UZK7_PLEOS	Catalase (Fragment) OS=Pleurotus ostreatus OX=5322 GN=cat2 PE=3 SV=1	26
12	tr|A0A067NHY5|A0A067NHY5_PLEOS	Catalase OS=Pleurotus ostreatus PC15 OX=1137138 GN=PLEOSDRAFT_1090819 PE=3 SV=1	26
13	tr|A0A067P0E7|A0A067P0E7_PLEOS	Uncharacterized protein OS=Pleurotus ostreatus PC15 OX=1137138 GN=PLEOSDRAFT_1087906 PE=3 SV=1	32
14	tr|A0A067P4U9|A0A067P4U9_PLEOS	Phosphopyruvate hydratase OS=Pleurotus ostreatus PC15 OX=1137138 GN=PLEOSDRAFT_1063312 PE=3 SV=1	37
15	tr|A0A067NRP1|A0A067NRP1_PLEOS	14_3_3 domain-containing protein OS=Pleurotus ostreatus PC15 OX=1137138 GN=PLEOSDRAFT_1093096 PE=3 SV=1	32
16	tr|A0A067NEB2|A0A067NEB2_PLEOS	Aldo_ket_red domain-containing protein OS=Pleurotus ostreatus PC15 OX=1137138 GN=PLEOSDRAFT_1090974 PE=4 SV=1	42
17	tr|A0A067N808|A0A067N808_PLEOS	Uncharacterized protein OS=Pleurotus ostreatus PC15 OX=1137138 GN=PLEOSDRAFT_1067979 PE=4 SV=1	24
18	tr|A0A067PCE4|A0A067PCE4_PLEOS	Uncharacterized protein OS=Pleurotus ostreatus PC15 OX=1137138 GN=PLEOSDRAFT_1052407 PE=3 SV=1	27
19	tr|A0A067N302|A0A067N302_PLEOS	Adenosylhomocysteinase OS=Pleurotus ostreatus PC15 OX=1137138 GN=PLEOSDRAFT_47087 PE=3 SV=1	20
20	tr|A0A067NS53|A0A067NS53_PLEOS	Pyruvate carboxylase OS=Pleurotus ostreatus PC15 OX=1137138	19

		GN=PLEOSDRAFT_1097340 PE=4 SV=1	
21	tr|A0A067N9N5|A0A067N9N5_PLEOS	Alpha-1,4 glucan phosphorylase OS=Pleurotus ostreatus PC15 OX=1137138 GN=PLEOSDRAFT_1058949 PE=3 SV=1	27
22	tr|A0A067NWG0|A0A067NWG0_PLEOS	Uncharacterized protein OS=Pleurotus ostreatus PC15 OX=1137138 GN=PLEOSDRAFT_185948 PE=4 SV=1	28
23	tr|A0A067NVZ7|A0A067NVZ7_PLEOS	D-fructose-6-phosphate amidotransferase OS=Pleurotus ostreatus PC15 OX=1137138 GN=PLEOSDRAFT_1062168 PE=4 SV=1	19
24	tr|A0A067NHM1|A0A067NHM1_PLEOS	Uncharacterized protein OS=Pleurotus ostreatus PC15 OX=1137138 GN=PLEOSDRAFT_1065831 PE=4 SV=1	18
25	tr|A0A067NV82|A0A067NV82_PLEOS	Pyruvate kinase OS=Pleurotus ostreatus PC15 OX=1137138 GN=PLEOSDRAFT_1088585 PE=3 SV=1	22
26	tr|A0A067NP23|A0A067NP23_PLEOS	Pleurotolysin B OS=Pleurotus ostreatus PC15 OX=1137138 GN=plyB PE=4 SV=1	18
27	tr|A0A067N6V2|A0A067N6V2_PLEOS	Uncharacterized protein OS=Pleurotus ostreatus PC15 OX=1137138 GN=PLEOSDRAFT_1072479 PE=4 SV=1	25
28	tr|W8SJ43|W8SJ43_PLEER	Glutamine synthetase OS=Pleurotus eryngii OX=5323 GN=PE-GS PE=3 SV=1	23
28	tr|A0A2R8G1D5|A0A2R8G1D5_PLEOS	Glutamine synthetase OS=Pleurotus ostreatus OX=5322 GN=GS PE=2 SV=1	23
28	tr|A0A067NQW2|A0A067NQW2_PLEOS	Glutamine synthetase OS=Pleurotus ostreatus PC15 OX=1137138 GN=PLEOSDRAFT_1089099 PE=3 SV=1	23
29	tr|A0A067NZL3|A0A067NZL3_PLEOS	Transaldolase OS=Pleurotus ostreatus PC15 OX=1137138 GN=PLEOSDRAFT_1061171 PE=3 SV=1	21
30	tr|A0A067NYL0|A0A067NYL0_PLEOS	UDP-glucose 6-dehydrogenase OS=Pleurotus ostreatus PC15 OX=1137138 GN=PLEOSDRAFT_1098969 PE=3 SV=1	17

31	tr|Q9UWF3|Q9UWF3_PLESA	Tubulin beta chain OS=Pleurotus sajor-caju OX=50053 PE=3 SV=1	22
31	tr|A0A067N725|A0A067N725_PLEOS	Tubulin beta chain OS=Pleurotus ostreatus PC15 OX=1137138 GN=PLEOSDRAFT_1091050 PE=3 SV=1	22
32	tr|A0A067NTI0|A0A067NTI0_PLEOS	ATP synthase subunit alpha OS=Pleurotus ostreatus PC15 OX=1137138 GN=ATP1 PE=3 SV=1	16
33	tr|A0A067NGZ1|A0A067NGZ1_PLEOS	Uncharacterized protein OS=Pleurotus ostreatus PC15 OX=1137138 GN=PLEOSDRAFT_1059525 PE=4 SV=1	14
34	tr|A0A067NJB6|A0A067NJB6_PLEOS	Carbohydrate-binding module family 12 protein OS=Pleurotus ostreatus PC15 OX=1137138 GN=PLEOSDRAFT_1089518 PE=4 SV=1	19
35	tr|A0A067NEK4|A0A067NEK4_PLEOS	Peptidyl-prolyl cis-trans isomerase OS=Pleurotus ostreatus PC15 OX=1137138 GN=PLEOSDRAFT_186045 PE=3 SV=1	26
36	tr|A0A067NGR8|A0A067NGR8_PLEOS	Ribos_L4_asso_C domain-containing protein OS=Pleurotus ostreatus PC15 OX=1137138 GN=PLEOSDRAFT_1044894 PE=3 SV=1	18
37	tr|A0A067NYU4|A0A067NYU4_PLEOS	Uncharacterized protein OS=Pleurotus ostreatus PC15 OX=1137138 GN=PLEOSDRAFT_1092681 PE=4 SV=1	42
38	tr|A0A067NQL5|A0A067NQL5_PLEOS	Uncharacterized protein OS=Pleurotus ostreatus PC15 OX=1137138 GN=PLEOSDRAFT_1094158 PE=4 SV=1	15
39	tr|A0A067NCW7|A0A067NCW7_PLEOS	Ostreolysin-like protein OS=Pleurotus ostreatus PC15 OX=1137138 GN=plyA PE=3 SV=1	32
39	sp|P83467|OLYA6_PLEOS	Ostreolysin A6 OS=Pleurotus ostreatus OX=5322 GN=OlyA6 PE=1 SV=2	32
40	tr|D2JY80|D2JY80_PLEOS	RRM domain-containing protein OS=Pleurotus ostreatus OX=5322 PE=2 SV=1	16

41	tr|A0A067NHY6|A0A067NHY6_PLEOS	Uncharacterized protein OS=Pleurotus ostreatus PC15 OX=1137138 GN=PLEOSDRAFT_51607 PE=3 SV=1	15
42	tr|A0A067NII7|A0A067NII7_PLEOS	Formate dehydrogenase OS=Pleurotus ostreatus PC15 OX=1137138 GN=PLEOSDRAFT_1071241 PE=3 SV=1	15
43	tr|A0A067N792|A0A067N792_PLEOS	Endoplasmic reticulum chaperone BiP OS=Pleurotus ostreatus PC15 OX=1137138 GN=PLEOSDRAFT_62237 PE=3 SV=1	16
44	tr|A0A067PC30|A0A067PC30_PLEOS	Uncharacterized protein OS=Pleurotus ostreatus PC15 OX=1137138 GN=PLEOSDRAFT_1087818 PE=3 SV=1	14
45	tr|A0A067N870|A0A067N870_PLEOS	Uncharacterized protein OS=Pleurotus ostreatus PC15 OX=1137138 GN=PLEOSDRAFT_1068034 PE=4 SV=1	12
46	tr|A0A067NI01|A0A067NI01_PLEOS	UTP--glucose-1-phosphate uridylyltransferase OS=Pleurotus ostreatus PC15 OX=1137138 GN=PLEOSDRAFT_1056178 PE=3 SV=1	16
47	tr|A0A067NXQ7|A0A067NXQ7_PLEOS	Uncharacterized protein OS=Pleurotus ostreatus PC15 OX=1137138 GN=PLEOSDRAFT_1074264 PE=3 SV=1	23
48	tr|A0A067NHG1|A0A067NHG1_PLEOS	Aldedh domain-containing protein OS=Pleurotus ostreatus PC15 OX=1137138 GN=PLEOSDRAFT_1090768 PE=3 SV=1	17
49	tr|A0A067P388|A0A067P388_PLEOS	Uncharacterized protein OS=Pleurotus ostreatus PC15 OX=1137138 GN=PLEOSDRAFT_1062827 PE=3 SV=1	11
50	tr|A0A067P0R2|A0A067P0R2_PLEOS	Tubulin alpha chain OS=Pleurotus ostreatus PC15 OX=1137138 GN=PLEOSDRAFT_1064256 PE=3 SV=1	18
51	tr|A0A067NSQ2|A0A067NSQ2_PLEOS	Inorganic diphosphatase OS=Pleurotus ostreatus PC15 OX=1137138 GN=PLEOSDRAFT_1075285 PE=3 SV=1	13
52	tr|A0A067NZG5|A0A067NZG5_PLEOS	40S ribosomal protein S7 OS=Pleurotus ostreatus PC15 OX=1137138	12

		GN=PLEOSDRAFT_1063761 PE=3 SV=1	
53	tr|A0A067NHP6|A0A067NHP6_PLEOS	40S ribosomal protein S0 OS=Pleurotus ostreatus PC15 OX=1137138 GN=RPS0 PE=3 SV=1	11
54	tr|A0A067NBU8|A0A067NBU8_PLEOS	Uncharacterized protein OS=Pleurotus ostreatus PC15 OX=1137138 GN=PLEOSDRAFT_1067243 PE=3 SV=1	12
55	tr|A0A067N3G4|A0A067N3G4_PLEOS	Uncharacterized protein OS=Pleurotus ostreatus PC15 OX=1137138 GN=PLEOSDRAFT_1068729 PE=3 SV=1	12
56	tr|A0A067NS51|A0A067NS51_PLEOS	KH type-2 domain-containing protein OS=Pleurotus ostreatus PC15 OX=1137138 GN=PLEOSDRAFT_1062741 PE=3 SV=1	9
57	tr|A0A067N8J8|A0A067N8J8_PLEOS	Uncharacterized protein OS=Pleurotus ostreatus PC15 OX=1137138 GN=PLEOSDRAFT_1090792 PE=4 SV=1	9
57	sp|O94739|CALM_PLEOS	Calmodulin OS=Pleurotus ostreatus OX=5322 GN=CMD1 PE=2 SV=3	9
57	sp|P11120|CALM_PLECO	Calmodulin OS=Pleurotus cornucopiae OX=5321 GN=CMD1 PE=1 SV=2	8
58	tr|A0A067N5V4|A0A067N5V4_PLEOS	Hemerythrin domain-containing protein OS=Pleurotus ostreatus PC15 OX=1137138 GN=PLEOSDRAFT_1059601 PE=4 SV=1	13
59	tr|A0A067NWH0|A0A067NWH0_PLEOS	Uncharacterized protein OS=Pleurotus ostreatus PC15 OX=1137138 GN=PLEOSDRAFT_1054419 PE=3 SV=1	10
60	tr|A0A067NP59|A0A067NP59_PLEOS	Multifunctional fusion protein OS=Pleurotus ostreatus PC15 OX=1137138 GN=PLEOSDRAFT_61563 PE=3 SV=1	7
61	tr|A0A067NDH7|A0A067NDH7_PLEOS	Uncharacterized protein OS=Pleurotus ostreatus PC15 OX=1137138 GN=PLEOSDRAFT_1090179 PE=4 SV=1	13
62	tr|A0A067NYA3|A0A067NYA3_PLEOS	60S ribosomal protein L20 OS=Pleurotus ostreatus PC15 OX=1137138	8

		GN=PLEOSDRAFT_1051744 PE=3 SV=1	
63	tr|A0A067P4S5|A0A067P4S5_PLEOS	60S ribosomal protein L13 OS=Pleurotus ostreatus PC15 OX=1137138 GN=PLEOSDRAFT_1088565 PE=3 SV=1	9
64	tr|A0A067PB97|A0A067PB97_PLEOS	Phosphoglycerate kinase OS=Pleurotus ostreatus PC15 OX=1137138 GN=PLEOSDRAFT_1087915 PE=3 SV=1	10
65	tr|A0A067NJ61|A0A067NJ61_PLEOS	Uncharacterized protein OS=Pleurotus ostreatus PC15 OX=1137138 GN=PLEOSDRAFT_1089494 PE=3 SV=1	7
66	tr|A0A067NQG0|A0A067NQG0_PLEOS	40S ribosomal protein S1 OS=Pleurotus ostreatus PC15 OX=1137138 GN=RPS1 PE=3 SV=1	10
67	tr|A0A067NT84|A0A067NT84_PLEOS	Sugar phosphate phosphatase OS=Pleurotus ostreatus PC15 OX=1137138 GN=PLEOSDRAFT_1036072 PE=3 SV=1	11
68	tr|A0A067NSE2|A0A067NSE2_PLEOS	Uncharacterized protein (Fragment) OS=Pleurotus ostreatus PC15 OX=1137138 GN=PLEOSDRAFT_1038239 PE=3 SV=1	7
69	tr|A0A067NXS6|A0A067NXS6_PLEOS	Glucose-6-phosphate 1-dehydrogenase OS=Pleurotus ostreatus PC15 OX=1137138 GN=PLEOSDRAFT_1088946 PE=3 SV=1	9
70	tr|A0A067NMS1|A0A067NMS1_PLEOS	Uncharacterized protein OS=Pleurotus ostreatus PC15 OX=1137138 GN=PLEOSDRAFT_1055325 PE=3 SV=1	8
71	tr|A0A067N4M3|A0A067N4M3_PLEOS	Thioredoxin domain-containing protein OS=Pleurotus ostreatus PC15 OX=1137138 GN=PLEOSDRAFT_1060176 PE=3 SV=1	11
71	tr|A0A067NGA1|A0A067NGA1_PLEOS	Thioredoxin domain-containing protein OS=Pleurotus ostreatus PC15 OX=1137138 GN=PLEOSDRAFT_1060173 PE=3 SV=1	10
71	tr|A0A067N4L6|A0A067N4L6_PLEOS	Thioredoxin domain-containing protein OS=Pleurotus ostreatus PC15	10

		OX=1137138 GN=PLEOSDRAFT_1050144 PE=3 SV=1	
72	tr|A0A067N544|A0A067N544_PLEOS	PKS_ER domain-containing protein OS=Pleurotus ostreatus PC15 OX=1137138 GN=PLEOSDRAFT_1080042 PE=4 SV=1	12
73	tr|A0A067NQ24|A0A067NQ24_PLEOS	Uncharacterized protein OS=Pleurotus ostreatus PC15 OX=1137138 GN=PLEOSDRAFT_1092017 PE=4 SV=1	6
74	tr|A0A067N9I3|A0A067N9I3_PLEOS	Rieske domain-containing protein OS=Pleurotus ostreatus PC15 OX=1137138 GN=PLEOSDRAFT_1113817 PE=4 SV=1	9
75	tr|A0A067NUA5|A0A067NUA5_PLEOS	S4 RNA-binding domain-containing protein OS=Pleurotus ostreatus PC15 OX=1137138 GN=PLEOSDRAFT_1054562 PE=3 SV=1	7
76	tr|A0A067NB23|A0A067NB23_PLEOS	Extracellular metalloproteinase OS=Pleurotus ostreatus PC15 OX=1137138 GN=PLEOSDRAFT_62198 PE=3 SV=1	14
77	tr|A0A067NS47|A0A067NS47_PLEOS	Septin-type G domain-containing protein OS=Pleurotus ostreatus PC15 OX=1137138 GN=PLEOSDRAFT_1088576 PE=3 SV=1	7
78	tr|A0A067NS76|A0A067NS76_PLEOS	Uncharacterized protein OS=Pleurotus ostreatus PC15 OX=1137138 GN=PLEOSDRAFT_1062759 PE=4 SV=1	7
79	tr|A0A067NL37|A0A067NL37_PLEOS	Aconitate hydratase, mitochondrial OS=Pleurotus ostreatus PC15 OX=1137138 GN=PLEOSDRAFT_1075708 PE=3 SV=1	7
80	tr|A0A067NIF5|A0A067NIF5_PLEOS	Ribosomal_S7 domain-containing protein OS=Pleurotus ostreatus PC15 OX=1137138 GN=PLEOSDRAFT_1064969 PE=3 SV=1	9
81	tr|A0A067NHZ5|A0A067NHZ5_PLEOS	40S ribosomal protein S8 OS=Pleurotus ostreatus PC15 OX=1137138	7

		GN=PLEOSDRAFT_1089421 PE=3 SV=1	
82	tr|A0A067NI86|A0A067NI86_PLEOS	Carbohydrate-binding module family 13 protein OS=Pleurotus ostreatus PC15 OX=1137138 GN=PLEOSDRAFT_1089845 PE=4 SV=1	13
83	tr|A0A067NR11|A0A067NR11_PLEOS	Protein disulfide-isomerase OS=Pleurotus ostreatus PC15 OX=1137138 GN=PLEOSDRAFT_1064231 PE=3 SV=1	8
84	tr|A0A067NIX1|A0A067NIX1_PLEOS	Malate dehydrogenase OS=Pleurotus ostreatus PC15 OX=1137138 GN=PLEOSDRAFT_1090652 PE=3 SV=1	12
85	tr|C4PFY6|C4PFY6_PLEOS	Peptidase 1 OS=Pleurotus ostreatus OX=5322 GN=POP1 PE=2 SV=1	9
85	tr|A0A067NRZ7|A0A067NRZ7_PLEOS	Uncharacterized protein OS=Pleurotus ostreatus PC15 OX=1137138 GN=PLEOSDRAFT_1088548 PE=3 SV=1	9
86	tr|A0A067NPJ8|A0A067NPJ8_PLEOS	Alpha-amylase OS=Pleurotus ostreatus PC15 OX=1137138 GN=PLEOSDRAFT_1095839 PE=3 SV=1	7
87	tr|A0A067NEY2|A0A067NEY2_PLEOS	60S ribosomal protein L8 (Fragment) OS=Pleurotus ostreatus PC15 OX=1137138 GN=PLEOSDRAFT_30562 PE=3 SV=1	7
88	tr|A0A067P101|A0A067P101_PLEOS	Ribosomal_L2_C domain-containing protein OS=Pleurotus ostreatus PC15 OX=1137138 GN=PLEOSDRAFT_1064355 PE=4 SV=1	7
89	tr|A0A067P6E3|A0A067P6E3_PLEOS	Histone H2B OS=Pleurotus ostreatus PC15 OX=1137138 GN=PLEOSDRAFT_1088177 PE=3 SV=1	8
89	tr|A0A067NZ73|A0A067NZ73_PLEOS	Histone H2B OS=Pleurotus ostreatus PC15 OX=1137138 GN=PLEOSDRAFT_42664 PE=3 SV=1	8
89	tr|A0A067NVC3|A0A067NVC3_PLEOS	Histone H2B OS=Pleurotus ostreatus PC15 OX=1137138 GN=PLEOSDRAFT_1088190 PE=3 SV=1	8
90	tr|G9MD63|G9MD63_PLEOS	Glyceraldehyde-3-phosphate	14

		dehydrogenase OS=Pleurotus ostreatus OX=5322 GN=gpd PE=2 SV=1	
90	tr|A0A067NKG0|A0A067NKG0_PLEOS	Glyceraldehyde-3-phosphate dehydrogenase OS=Pleurotus ostreatus PC15 OX=1137138 GN=PLEOSDRAFT_1090663 PE=3 SV=1	14
91	tr|A0A067NRB3|A0A067NRB3_PLEOS	Uncharacterized protein OS=Pleurotus ostreatus PC15 OX=1137138 GN=PLEOSDRAFT_1085408 PE=3 SV=1	18
92	tr|A0A067NL73|A0A067NL73_PLEOS	Malate dehydrogenase OS=Pleurotus ostreatus PC15 OX=1137138 GN=PLEOSDRAFT_1088858 PE=3 SV=1	18
93	tr|A0A067NZS0|A0A067NZS0_PLEOS	ATP synthase subunit beta OS=Pleurotus ostreatus PC15 OX=1137138 GN=ATP2 PE=3 SV=1	12
94	tr|A0A067PAP5|A0A067PAP5_PLEOS	GTP-binding nuclear protein OS=Pleurotus ostreatus PC15 OX=1137138 GN=PLEOSDRAFT_1061152 PE=3 SV=1	6
95	tr|A0A067N7U6|A0A067N7U6_PLEOS	Uncharacterized protein OS=Pleurotus ostreatus PC15 OX=1137138 GN=PLEOSDRAFT_1072574 PE=4 SV=1	10
96	tr|A0A067PA05|A0A067PA05_PLEOS	Succinate dehydrogenase [ubiquinone] flavoprotein subunit, mitochondrial OS=Pleurotus ostreatus PC15 OX=1137138 GN=SDH1 PE=3 SV=1	5
97	tr|A0A067P0D8|A0A067P0D8_PLEOS	H(+)-transporting two-sector ATPase OS=Pleurotus ostreatus PC15 OX=1137138 GN=PLEOSDRAFT_1061285 PE=3 SV=1	6
98	tr|A0A067P301|A0A067P301_PLEOS	Ribosomal protein L15 OS=Pleurotus ostreatus PC15 OX=1137138 GN=PLEOSDRAFT_1088526 PE=3 SV=1	9
98	tr|D2JY79|D2JY79_PLEOS	Ribosomal protein L15 OS=Pleurotus ostreatus OX=5322 PE=2 SV=1	9
99	tr|A0A067NUF2|A0A067NUF2_PLEOS	Ribosomal_S13_N domain-containing protein OS=Pleurotus ostreatus PC15 OX=1137138 GN=PLEOSDRAFT_1091806	6

		PE=3 SV=1	
100	tr|A0A067NHE8|A0A067NHE8_PLEOS	Uncharacterized protein OS=Pleurotus ostreatus PC15 OX=1137138 GN=PLEOSDRAFT_1077368 PE=4 SV=1	12
101	tr|A0A067NW47|A0A067NW47_PLEOS	S5 DRBM domain-containing protein OS=Pleurotus ostreatus PC15 OX=1137138 GN=PLEOSDRAFT_1089478 PE=3 SV=1	9
102	tr|A0A067N762|A0A067N762_PLEOS	Uncharacterized protein (Fragment) OS=Pleurotus ostreatus PC15 OX=1137138 GN=PLEOSDRAFT_1090573 PE=4 SV=1	8
103	tr|A0A067P4P1|A0A067P4P1_PLEOS	Ribosomal_S17_N domain-containing protein OS=Pleurotus ostreatus PC15 OX=1137138 GN=PLEOSDRAFT_1053977 PE=3 SV=1	5
104	tr|A0A387LBC4|A0A387LBC4_PLEOS	Manganese lipoxygenase OS=Pleurotus ostreatus OX=5322 GN=lox1 PE=2 SV=1	6
104	tr|A0A067N2A8|A0A067N2A8_PLEOS	Manganese lipoxygenase OS=Pleurotus ostreatus PC15 OX=1137138 GN=PLEOSDRAFT_1068582 PE=4 SV=1	6
105	tr|A0A067NMW5|A0A067NMW5_PLEOS	Peptidase A1 domain-containing protein (Fragment) OS=Pleurotus ostreatus PC15 OX=1137138 GN=PLEOSDRAFT_1055405 PE=3 SV=1	5
105	tr|U6A6W7|U6A6W7_PLEOS	Aspartic protease OS=Pleurotus ostreatus OX=5322 PE=2 SV=1	4
106	tr|A0A067NYR0|A0A067NYR0_PLEOS	Uncharacterized protein OS=Pleurotus ostreatus PC15 OX=1137138 GN=PLEOSDRAFT_172505 PE=4 SV=1	5
107	tr|A0A067PBA8|A0A067PBA8_PLEOS	Nucleoside diphosphate kinase OS=Pleurotus ostreatus PC15 OX=1137138 GN=PLEOSDRAFT_1052542 PE=3 SV=1	10
108	tr|A0A067P305|A0A067P305_PLEOS	Transketolase OS=Pleurotus ostreatus PC15 OX=1137138 GN=PLEOSDRAFT_172379 PE=3 SV=1	7

109	tr|A0A067NJU2|A0A067NJU2_PLEOS	Uncharacterized protein OS=Pleurotus ostreatus PC15 OX=1137138 GN=PLEOSDRAFT_1113759 PE=4 SV=1	9
110	tr|A0A067NHX4|A0A067NHX4_PLEOS	Malate synthase OS=Pleurotus ostreatus PC15 OX=1137138 GN=PLEOSDRAFT_1089338 PE=3 SV=1	5
111	tr|A0A067P2E6|A0A067P2E6_PLEOS	Septin-type G domain-containing protein OS=Pleurotus ostreatus PC15 OX=1137138 GN=PLEOSDRAFT_1088443 PE=3 SV=1	7
112	tr|A0A067NV87|A0A067NV87_PLEOS	Glucose-6-phosphate isomerase OS=Pleurotus ostreatus PC15 OX=1137138 GN=PLEOSDRAFT_1064869 PE=3 SV=1	7
113	tr|A0A067PC39|A0A067PC39_PLEOS	Uncharacterized protein OS=Pleurotus ostreatus PC15 OX=1137138 GN=PLEOSDRAFT_1069512 PE=4 SV=1	16
114	tr|A0A067NFT1|A0A067NFT1_PLEOS	40S ribosomal protein S4 OS=Pleurotus ostreatus PC15 OX=1137138 GN=PLEOSDRAFT_1066177 PE=3 SV=1	8
115	tr|A0A067N3K5|A0A067N3K5_PLEOS	NmrA domain-containing protein OS=Pleurotus ostreatus PC15 OX=1137138 GN=PLEOSDRAFT_174947 PE=4 SV=1	5
115	tr|A0A067NFQ9|A0A067NFQ9_PLEOS	NmrA domain-containing protein OS=Pleurotus ostreatus PC15 OX=1137138 GN=PLEOSDRAFT_1049893 PE=4 SV=1	4
116	tr|A0A067NUL0|A0A067NUL0_PLEOS	Pyrid_ox_like domain-containing protein OS=Pleurotus ostreatus PC15 OX=1137138 GN=PLEOSDRAFT_1056316 PE=4 SV=1	6
117	tr|A0A067NG66|A0A067NG66_PLEOS	Uncharacterized protein OS=Pleurotus ostreatus PC15 OX=1137138 GN=PLEOSDRAFT_1085281 PE=4 SV=1	6
118	tr|A0A067NLX2|A0A067NLX2_PLEOS	Cytochrome b5 heme-binding domain-containing protein OS=Pleurotus ostreatus PC15 OX=1137138	6

		GN=PLEOSDRAFT_1065325 PE=4 SV=1	
119	tr|A0A067NYZ2|A0A067NYZ2_PLEOS	Polyadenylate-binding protein OS=Pleurotus ostreatus PC15 OX=1137138 GN=PLEOSDRAFT_172520 PE=3 SV=1	5
120	tr|A0A067NMU9|A0A067NMU9_PLEOS	60S ribosomal protein L36 OS=Pleurotus ostreatus PC15 OX=1137138 GN=PLEOSDRAFT_1070894 PE=3 SV=1	5
121	tr|A0A067NAD6|A0A067NAD6_PLEOS	Uncharacterized protein OS=Pleurotus ostreatus PC15 OX=1137138 GN=PLEOSDRAFT_1067950 PE=3 SV=1	6
122	tr|A0A067P0M5|A0A067P0M5_PLEOS	Ribosomal_L18_c domain-containing protein (Fragment) OS=Pleurotus ostreatus PC15 OX=1137138 GN=PLEOSDRAFT_1055401 PE=3 SV=1	5
123	tr|A0A067N8D2|A0A067N8D2_PLEOS	RRM domain-containing protein OS=Pleurotus ostreatus PC15 OX=1137138 GN=PLEOSDRAFT_1090676 PE=4 SV=1	15
124	tr|A0A067NY35|A0A067NY35_PLEOS	M20_dimer domain-containing protein OS=Pleurotus ostreatus PC15 OX=1137138 GN=PLEOSDRAFT_1087624 PE=3 SV=1	4
125	tr|A0A067NSA9|A0A067NSA9_PLEOS	Rab GDP dissociation inhibitor OS=Pleurotus ostreatus PC15 OX=1137138 GN=PLEOSDRAFT_1054110 PE=3 SV=1	7
126	sp|P81055|PLMP_PLEOS	Peptidyl-Lys metalloendopeptidase OS=Pleurotus ostreatus OX=5322 GN=MEP PE=1 SV=1	12
126	tr|A0A067NSY7|A0A067NSY7_PLEOS	Aspzincin_M35 domain-containing protein OS=Pleurotus ostreatus PC15 OX=1137138 GN=PLEOSDRAFT_1037634 PE=3 SV=1	11
127	tr|A0A067NI26|A0A067NI26_PLEOS	HTH cro/C1-type domain-containing protein OS=Pleurotus ostreatus PC15 OX=1137138 GN=PLEOSDRAFT_1114418 PE=3 SV=1	6

128	tr|A0A067NWY2|A0A067NWY2_PLEOS	Uncharacterized protein OS=Pleurotus ostreatus PC15 OX=1137138 GN=PLEOSDRAFT_24760 PE=4 SV=1	6
129	tr|A0A067NBU9|A0A067NBU9_PLEOS	NAD-specific glutamate dehydrogenase OS=Pleurotus ostreatus PC15 OX=1137138 GN=PLEOSDRAFT_40761 PE=3 SV=1	4
130	tr|A0A067NG95|A0A067NG95_PLEOS	Dipeptidyl-peptidase V OS=Pleurotus ostreatus PC15 OX=1137138 GN=PLEOSDRAFT_1087304 PE=3 SV=1	7
131	tr|A0A067NFV5|A0A067NFV5_PLEOS	Uncharacterized protein OS=Pleurotus ostreatus PC15 OX=1137138 GN=PLEOSDRAFT_1113268 PE=4 SV=1	7
132	tr|A0A067P657|A0A067P657_PLEOS	Aldo_ket_red domain-containing protein OS=Pleurotus ostreatus PC15 OX=1137138 GN=PLEOSDRAFT_1053140 PE=4 SV=1	4
133	tr|D2JY73|D2JY73_PLEOS	Ribosomal_L16 domain-containing protein OS=Pleurotus ostreatus OX=5322 PE=2 SV=1	8
133	tr|A0A067NTH6|A0A067NTH6_PLEOS	Ribosomal_L16 domain-containing protein OS=Pleurotus ostreatus PC15 OX=1137138 GN=PLEOSDRAFT_1089909 PE=4 SV=1	8
134	tr|A0A067P3U3|A0A067P3U3_PLEOS	Aldo_ket_red domain-containing protein OS=Pleurotus ostreatus PC15 OX=1137138 GN=PLEOSDRAFT_1102061 PE=4 SV=1	5
135	tr|A0A067PAP3|A0A067PAP3_PLEOS	PKS_ER domain-containing protein OS=Pleurotus ostreatus PC15 OX=1137138 GN=PLEOSDRAFT_1098934 PE=3 SV=1	6
136	tr|A0A067NM16|A0A067NM16_PLEOS	60S acidic ribosomal protein P0 OS=Pleurotus ostreatus PC15 OX=1137138 GN=PLEOSDRAFT_1090404 PE=3 SV=1	5
137	tr|A0A067NJH3|A0A067NJH3_PLEOS	Uncharacterized protein OS=Pleurotus ostreatus PC15 OX=1137138	4

		GN=PLEOSDRAFT_1093456 PE=4 SV=1	
138	tr|A0A067NL23|A0A067NL23_PLEOS	Uncharacterized protein OS=Pleurotus ostreatus PC15 OX=1137138 GN=PLEOSDRAFT_1088887 PE=4 SV=1	5
139	tr|A0A067NED1|A0A067NED1_PLEOS	Coronin OS=Pleurotus ostreatus PC15 OX=1137138 GN=PLEOSDRAFT_1056973 PE=3 SV=1	5
140	tr|A0A067N8V5|A0A067N8V5_PLEOS	Peptidase_M24 domain-containing protein OS=Pleurotus ostreatus PC15 OX=1137138 GN=PLEOSDRAFT_1067285 PE=3 SV=1	4
141	tr|A0A067NZD8|A0A067NZD8_PLEOS	Small COPII coat GTPase SAR1 OS=Pleurotus ostreatus PC15 OX=1137138 GN=PLEOSDRAFT_1052294 PE=3 SV=1	4
142	tr|A0A067NKF5|A0A067NKF5_PLEOS	Uncharacterized protein OS=Pleurotus ostreatus PC15 OX=1137138 GN=PLEOSDRAFT_1071507 PE=4 SV=1	4
143	tr|A0A067NT90|A0A067NT90_PLEOS	Citrulline--aspartate ligase OS=Pleurotus ostreatus PC15 OX=1137138 GN=PLEOSDRAFT_1113222 PE=3 SV=1	5
144	tr|A0A067NSK8|A0A067NSK8_PLEOS	Uncharacterized protein OS=Pleurotus ostreatus PC15 OX=1137138 GN=PLEOSDRAFT_1088636 PE=4 SV=1	5
145	tr|A0A067P077|A0A067P077_PLEOS	KOW domain-containing protein (Fragment) OS=Pleurotus ostreatus PC15 OX=1137138 GN=PLEOSDRAFT_1016358 PE=3 SV=1	8
146	tr|A0A067NJC3|A0A067NJC3_PLEOS	WD_REPEATS_REGION domain-containing protein OS=Pleurotus ostreatus PC15 OX=1137138 GN=PLEOSDRAFT_1065257 PE=4 SV=1	7
147	tr|A0A067NFC5|A0A067NFC5_PLEOS	Uncharacterized protein OS=Pleurotus ostreatus PC15 OX=1137138 GN=PLEOSDRAFT_1089796 PE=3 SV=1	6
148	tr|A0A067P2S5|A0A067P2S5_PLEOS	Uncharacterized protein OS=Pleurotus ostreatus PC15 OX=1137138	5

		GN=PLEOSDRAFT_1053876 PE=3 SV=1	
149	tr|A0A067P2R6|A0A067P2R6_PLEOS	Aminopeptidase OS=Pleurotus ostreatus PC15 OX=1137138 GN=PLEOSDRAFT_1061186 PE=3 SV=1	4
150	tr|A0A067NXY7|A0A067NXY7_PLEOS	60S ribosomal protein L27 OS=Pleurotus ostreatus PC15 OX=1137138 GN=PLEOSDRAFT_1087603 PE=3 SV=1	7
151	tr|Q96TW1|Q96TW1_PLEOS	Putative ubiquitin (Fragment) OS=Pleurotus ostreatus OX=5322 GN=ubq1 PE=2 SV=1	12
151	tr|A0A067P7C4|A0A067P7C4_PLEOS	Uncharacterized protein OS=Pleurotus ostreatus PC15 OX=1137138 GN=PLEOSDRAFT_1081506 PE=4 SV=1	14
151	tr|A0A067P413|A0A067P413_PLEOS	Uncharacterized protein OS=Pleurotus ostreatus PC15 OX=1137138 GN=PLEOSDRAFT_1088454 PE=4 SV=1	12
151	tr|A0A067NZF5|A0A067NZF5_PLEOS	Ubiquitin-like domain-containing protein OS=Pleurotus ostreatus PC15 OX=1137138 GN=PLEOSDRAFT_1074537 PE=4 SV=1	12
151	tr|A0A067NR75|A0A067NR75_PLEOS	Ubiquitin-like domain-containing protein OS=Pleurotus ostreatus PC15 OX=1137138 GN=PLEOSDRAFT_1113129 PE=3 SV=1	12
151	tr|A0A067N860|A0A067N860_PLEOS	Uncharacterized protein OS=Pleurotus ostreatus PC15 OX=1137138 GN=PLEOSDRAFT_1058300 PE=4 SV=1	12
151	tr|A0A067NWI7|A0A067NWI7_PLEOS	Uncharacterized protein OS=Pleurotus ostreatus PC15 OX=1137138 GN=PLEOSDRAFT_1082432 PE=4 SV=1	12
152	tr|A0A067NWP3|A0A067NWP3_PLEOS	Uncharacterized protein OS=Pleurotus ostreatus PC15 OX=1137138 GN=PLEOSDRAFT_1073059 PE=4 SV=1	6
153	tr|A0A067NYE7|A0A067NYE7_PLEOS	Peptide hydrolase OS=Pleurotus ostreatus PC15 OX=1137138 GN=PLEOSDRAFT_1089035 PE=3 SV=1	4
154	tr|A0A067NYE0|A0A067NYE0_PLEOS	Uncharacterized protein OS=Pleurotus	4

		ostreatus PC15 OX=1137138 GN=PLEOSDRAFT_1063949 PE=4 SV=1	
155	tr|A0A067NP91|A0A067NP91_PLEOS	Ribosomal protein OS=Pleurotus ostreatus PC15 OX=1137138 GN=PLEOSDRAFT_1055807 PE=3 SV=1	5
156	tr|A0A067NYA5|A0A067NYA5_PLEOS	Carbohydrate-binding module family 13 protein OS=Pleurotus ostreatus PC15 OX=1137138 GN=PLEOSDRAFT_1034502 PE=4 SV=1	6
157	tr|A0A067PB21|A0A067PB21_PLEOS	Uncharacterized protein OS=Pleurotus ostreatus PC15 OX=1137138 GN=PLEOSDRAFT_1091325 PE=4 SV=1	7
158	tr|A0A067N510|A0A067N510_PLEOS	Uncharacterized protein OS=Pleurotus ostreatus PC15 OX=1137138 GN=PLEOSDRAFT_1110004 PE=3 SV=1	5
159	tr|A0A067NMW1|A0A067NMW1_PLEOS	S10_plectin domain-containing protein OS=Pleurotus ostreatus PC15 OX=1137138 GN=PLEOSDRAFT_1089113 PE=4 SV=1	7
160	tr|A0A067PBZ8|A0A067PBZ8_PLEOS	Dynamin-type G domain-containing protein OS=Pleurotus ostreatus PC15 OX=1137138 GN=PLEOSDRAFT_1110788 PE=3 SV=1	4
161	tr|A0A067P7Q0|A0A067P7Q0_PLEOS	Ribosomal_L18e/L15P domain-containing protein OS=Pleurotus ostreatus PC15 OX=1137138 GN=PLEOSDRAFT_1053176 PE=3 SV=1	6
162	tr|A0A067NTY2|A0A067NTY2_PLEOS	Uncharacterized protein OS=Pleurotus ostreatus PC15 OX=1137138 GN=PLEOSDRAFT_1111325 PE=4 SV=1	5
163	tr|A0A067NDM8|A0A067NDM8_PLEOS	Uncharacterized protein OS=Pleurotus ostreatus PC15 OX=1137138 GN=PLEOSDRAFT_1058122 PE=3 SV=1	5
164	tr|A0A067NS38|A0A067NS38_PLEOS	Uncharacterized protein OS=Pleurotus ostreatus PC15 OX=1137138 GN=PLEOSDRAFT_1101731 PE=4 SV=1	4
165	tr|A0A067NZ52|A0A067NZ52_PLEOS	Ribosomal_L18e/L15P domain-containing protein OS=Pleurotus ostreatus PC15	7

		OX=1137138 GN=PLEOSDRAFT_1063630 PE=3 SV=1	
166	tr|A0A067NV48|A0A067NV48_PLEOS	PKS_ER domain-containing protein OS=Pleurotus ostreatus PC15 OX=1137138 GN=PLEOSDRAFT_1091892 PE=4 SV=1	4
167	tr|A0A067P0D4|A0A067P0D4_PLEOS	Proteasome subunit alpha type OS=Pleurotus ostreatus PC15 OX=1137138 GN=PLEOSDRAFT_1052612 PE=3 SV=1	6
168	tr|A0A067NZZ6|A0A067NZZ6_PLEOS	Uncharacterized protein (Fragment) OS=Pleurotus ostreatus PC15 OX=1137138 GN=PLEOSDRAFT_1087899 PE=4 SV=1	5
169	tr|A0A067NLW0|A0A067NLW0_PLEOS	UBA_e1_C domain-containing protein OS=Pleurotus ostreatus PC15 OX=1137138 GN=PLEOSDRAFT_1063833 PE=3 SV=1	4
170	tr|A0A067NEV8|A0A067NEV8_PLEOS	Uncharacterized protein OS=Pleurotus ostreatus PC15 OX=1137138 GN=PLEOSDRAFT_1090064 PE=3 SV=1	7
171	tr|A0A067NXJ3|A0A067NXJ3_PLEOS	Uncharacterized protein OS=Pleurotus ostreatus PC15 OX=1137138 GN=PLEOSDRAFT_1054789 PE=3 SV=1	9
172	tr|A0A067NQV4|A0A067NQV4_PLEOS	Uncharacterized protein OS=Pleurotus ostreatus PC15 OX=1137138 GN=PLEOSDRAFT_1055341 PE=3 SV=1	6
173	tr|A0A067PC25|A0A067PC25_PLEOS	E3 ubiquitin ligase complex SCF subunit OS=Pleurotus ostreatus PC15 OX=1137138 GN=PLEOSDRAFT_1091493 PE=3 SV=1	5
174	tr|A0A067NZB3|A0A067NZB3_PLEOS	Uncharacterized protein OS=Pleurotus ostreatus PC15 OX=1137138 GN=PLEOSDRAFT_1112019 PE=3 SV=1	3
175	tr|A0A067NKQ1|A0A067NKQ1_PLEOS	MaoC-like domain-containing protein OS=Pleurotus ostreatus PC15 OX=1137138 GN=PLEOSDRAFT_41213 PE=4 SV=1	3

176	tr|A0A067N7V6|A0A067N7V6_PLEOS	Uncharacterized protein OS=Pleurotus ostreatus PC15 OX=1137138 GN=PLEOSDRAFT_1090683 PE=4 SV=1	5
177	tr|A0A067NER0|A0A067NER0_PLEOS	Tubulin alpha chain OS=Pleurotus ostreatus PC15 OX=1137138 GN=PLEOSDRAFT_1066547 PE=3 SV=1	16
178	tr|A0A067NKW8|A0A067NKW8_PLEOS	Fructose-bisphosphate aldolase OS=Pleurotus ostreatus PC15 OX=1137138 GN=PLEOSDRAFT_1088861 PE=3 SV=1	5
179	tr|A0A067NQG3|A0A067NQG3_PLEOS	Mitochondrial intermediate peptidase OS=Pleurotus ostreatus PC15 OX=1137138 GN=PLEOSDRAFT_160461 PE=3 SV=1	5
180	tr|A0A067NMV7|A0A067NMV7_PLEOS	Uncharacterized protein OS=Pleurotus ostreatus PC15 OX=1137138 GN=PLEOSDRAFT_1093998 PE=4 SV=1	5
181	tr|A0A067N929|A0A067N929_PLEOS	Ketol-acid reductoisomerase, mitochondrial OS=Pleurotus ostreatus PC15 OX=1137138 GN=PLEOSDRAFT_1090361 PE=3 SV=1	3
182	tr|A0A067NQW4|A0A067NQW4_PLEOS	Superoxide dismutase OS=Pleurotus ostreatus PC15 OX=1137138 GN=PLEOSDRAFT_1095940 PE=3 SV=1	4
183	tr|A0A067NPE8|A0A067NPE8_PLEOS	40S ribosomal protein S12 OS=Pleurotus ostreatus PC15 OX=1137138 GN=PLEOSDRAFT_1055766 PE=3 SV=1	9
184	tr|A0A067NSN4|A0A067NSN4_PLEOS	Methylmalonate-semialdehyde dehydrogenase (CoA acylating) OS=Pleurotus ostreatus PC15 OX=1137138 GN=PLEOSDRAFT_1101890 PE=4 SV=1	5
185	tr|A0A067NJY1|A0A067NJY1_PLEOS	Uncharacterized protein OS=Pleurotus ostreatus PC15 OX=1137138 GN=PLEOSDRAFT_1093556 PE=3 SV=1	4
186	tr|A0A067NJ47|A0A067NJ47_PLEOS	Uncharacterized protein OS=Pleurotus ostreatus PC15 OX=1137138 GN=PLEOSDRAFT_173733 PE=4 SV=1	4

187	tr|A0A067NHP3|A0A067NHP3_PLEOS	Uncharacterized protein OS=Pleurotus ostreatus PC15 OX=1137138 GN=PLEOSDRAFT_1090794 PE=4 SV=1	3
188	tr|A0A067NDZ6|A0A067NDZ6_PLEOS	Proteasome subunit beta OS=Pleurotus ostreatus PC15 OX=1137138 GN=PLEOSDRAFT_1072122 PE=3 SV=1	3
189	tr|A0A067NI38|A0A067NI38_PLEOS	Triosephosphate isomerase OS=Pleurotus ostreatus PC15 OX=1137138 GN=PLEOSDRAFT_1090849 PE=3 SV=1	5
190	tr|A0A067NX51|A0A067NX51_PLEOS	Uncharacterized protein OS=Pleurotus ostreatus PC15 OX=1137138 GN=PLEOSDRAFT_1060402 PE=3 SV=1	5
191	tr|A0A067NRV5|A0A067NRV5_PLEOS	Uncharacterized protein OS=Pleurotus ostreatus PC15 OX=1137138 GN=PLEOSDRAFT_1088487 PE=4 SV=1	3
191	tr|A0A067NBZ4|A0A067NBZ4_PLEOS	Uncharacterized protein OS=Pleurotus ostreatus PC15 OX=1137138 GN=PLEOSDRAFT_1113407 PE=3 SV=1	2
192	tr|A0A067NQJ2|A0A067NQJ2_PLEOS	NAD(P)-bd_dom domain-containing protein OS=Pleurotus ostreatus PC15 OX=1137138 GN=PLEOSDRAFT_1112199 PE=4 SV=1	4
193	tr|A0A067PAB9|A0A067PAB9_PLEOS	Uncharacterized protein OS=Pleurotus ostreatus PC15 OX=1137138 GN=PLEOSDRAFT_1073596 PE=3 SV=1	3
194	tr|A0A067P2V4|A0A067P2V4_PLEOS	Uncharacterized protein OS=Pleurotus ostreatus PC15 OX=1137138 GN=PLEOSDRAFT_1092036 PE=4 SV=1	3
194	tr|A0A067NT65|A0A067NT65_PLEOS	APH domain-containing protein OS=Pleurotus ostreatus PC15 OX=1137138 GN=PLEOSDRAFT_1111200 PE=4 SV=1	2
195	tr|A0A067NWN7|A0A067NWN7_PLEOS	Actin-related protein 2/3 complex subunit 4 OS=Pleurotus ostreatus PC15 OX=1137138 GN=PLEOSDRAFT_61651 PE=3 SV=1	3
196	tr|A0A067NQW1|A0A067NQW1_PLEOS	Trehalase OS=Pleurotus ostreatus PC15 OX=1137138 GN=PLEOSDRAFT_1094192	3

		PE=3 SV=1	
197	tr|A0A067NL04|A0A067NL04_PLEOS	Uncharacterized protein OS=Pleurotus ostreatus PC15 OX=1137138 GN=PLEOSDRAFT_1090448 PE=4 SV=1	3
198	tr|A0A067NBT8|A0A067NBT8_PLEOS	PKS_ER domain-containing protein OS=Pleurotus ostreatus PC15 OX=1137138 GN=PLEOSDRAFT_1106245 PE=4 SV=1	4
199	tr|A0A067P3F6|A0A067P3F6_PLEOS	Uncharacterized protein OS=Pleurotus ostreatus PC15 OX=1137138 GN=PLEOSDRAFT_1053605 PE=4 SV=1	3
200	tr|A0A067NG45|A0A067NG45_PLEOS	Glycosyltransferase family 69 protein OS=Pleurotus ostreatus PC15 OX=1137138 GN=PLEOSDRAFT_1114678 PE=3 SV=1	5
201	tr|A0A067NTZ1|A0A067NTZ1_PLEOS	Mitogen-activated protein kinase OS=Pleurotus ostreatus PC15 OX=1137138 GN=PLEOSDRAFT_38836 PE=3 SV=1	4
202	tr|A0A067P3W4|A0A067P3W4_PLEOS	Histone H4 OS=Pleurotus ostreatus PC15 OX=1137138 GN=PLEOSDRAFT_1088653 PE=3 SV=1	5
202	tr|A0A067NTA7|A0A067NTA7_PLEOS	Histone H4 OS=Pleurotus ostreatus PC15 OX=1137138 GN=PLEOSDRAFT_1089880 PE=3 SV=1	5
203	tr|A0A067NY71|A0A067NY71_PLEOS	Uncharacterized protein OS=Pleurotus ostreatus PC15 OX=1137138 GN=PLEOSDRAFT_1063873 PE=3 SV=1	3
204	tr|A0A067NZB1|A0A067NZB1_PLEOS	S-adenosylmethionine synthase OS=Pleurotus ostreatus PC15 OX=1137138 GN=PLEOSDRAFT_1052256 PE=3 SV=1	4
205	tr|A0A067NXN9|A0A067NXN9_PLEOS	Uncharacterized protein OS=Pleurotus ostreatus PC15 OX=1137138 GN=PLEOSDRAFT_1088928 PE=4 SV=1	3
206	tr|A0A067NFH4|A0A067NFH4_PLEOS	Uncharacterized protein OS=Pleurotus ostreatus PC15 OX=1137138	5

		GN=PLEOSDRAFT_1057271 PE=3 SV=1	
207	tr|A0A067NWF1|A0A067NWF1_PLEOS	Phosphoenolpyruvate carboxykinase (ATP) OS=Pleurotus ostreatus PC15 OX=1137138 GN=PLEOSDRAFT_1089535 PE=3 SV=1	3
208	tr|A0A067NTK9|A0A067NTK9_PLEOS	Uncharacterized protein OS=Pleurotus ostreatus PC15 OX=1137138 GN=PLEOSDRAFT_1088755 PE=3 SV=1	3
209	tr|A0A067NZJ1|A0A067NZJ1_PLEOS	Proteasome subunit alpha type OS=Pleurotus ostreatus PC15 OX=1137138 GN=PLEOSDRAFT_185889 PE=3 SV=1	3
210	tr|A0A067NEN6|A0A067NEN6_PLEOS	Core subunit of the ubiquinol-cytochrome c reductase complex, QCR1 OS=Pleurotus ostreatus PC15 OX=1137138 GN=QCR1 PE=3 SV=1	4
211	tr|A0A067NKH2|A0A067NKH2_PLEOS	Aldo_ket_red domain-containing protein OS=Pleurotus ostreatus PC15 OX=1137138 GN=PLEOSDRAFT_1089691 PE=4 SV=1	3
212	tr|A0A067NHH3|A0A067NHH3_PLEOS	Aldedh domain-containing protein OS=Pleurotus ostreatus PC15 OX=1137138 GN=PLEOSDRAFT_1114338 PE=3 SV=1	6
213	tr|A0A067NU43|A0A067NU43_PLEOS	Adenosine kinase OS=Pleurotus ostreatus PC15 OX=1137138 GN=PLEOSDRAFT_1088759 PE=3 SV=1	3
214	tr|A0A067NU68|A0A067NU68_PLEOS	Thioredoxin OS=Pleurotus ostreatus PC15 OX=1137138 GN=PLEOSDRAFT_1088775 PE=3 SV=1	4
215	tr|A0A067N7P1|A0A067N7P1_PLEOS	Uncharacterized protein OS=Pleurotus ostreatus PC15 OX=1137138 GN=PLEOSDRAFT_177083 PE=4 SV=1	3
216	tr|A0A067NF95|A0A067NF95_PLEOS	Septin-type G domain-containing protein OS=Pleurotus ostreatus PC15 OX=1137138 GN=PLEOSDRAFT_1060156 PE=3 SV=1	6

217	tr|A0A067NEZ0|A0A067NEZ0_PLEOS	Uncharacterized protein OS=Pleurotus ostreatus PC15 OX=1137138 GN=PLEOSDRAFT_1089818 PE=4 SV=1	4
218	tr|A0A067P1P5|A0A067P1P5_PLEOS	Uncharacterized protein OS=Pleurotus ostreatus PC15 OX=1137138 GN=PLEOSDRAFT_1110835 PE=3 SV=1	6
219	tr|A0A067NU35|A0A067NU35_PLEOS	T-complex protein 1 subunit gamma OS=Pleurotus ostreatus PC15 OX=1137138 GN=PLEOSDRAFT_1088756 PE=3 SV=1	3
220	tr|A0A067NJ32|A0A067NJ32_PLEOS	Peptidase_M43 domain-containing protein OS=Pleurotus ostreatus PC15 OX=1137138 GN=PLEOSDRAFT_1067850 PE=3 SV=1	4
220	tr|Q5Y972|Q5Y972_PLEOS	Metalloprotease OS=Pleurotus ostreatus OX=5322 PE=2 SV=1	3
221	tr|A0A067NTP4|A0A067NTP4_PLEOS	40S ribosomal protein S26 (Fragment) OS=Pleurotus ostreatus PC15 OX=1137138 GN=PLEOSDRAFT_4300 PE=3 SV=1	4
222	tr|A0A067P1A7|A0A067P1A7_PLEOS	SCP domain-containing protein OS=Pleurotus ostreatus PC15 OX=1137138 GN=PLEOSDRAFT_37051 PE=4 SV=1	3
222	tr|A0A067PCE7|A0A067PCE7_PLEOS	SCP domain-containing protein OS=Pleurotus ostreatus PC15 OX=1137138 GN=PLEOSDRAFT_37057 PE=4 SV=1	2
223	tr|A0A067NM35|A0A067NM35_PLEOS	Uncharacterized protein OS=Pleurotus ostreatus PC15 OX=1137138 GN=PLEOSDRAFT_1055097 PE=3 SV=1	4
224	tr|A0A067PBX8|A0A067PBX8_PLEOS	Uncharacterized protein OS=Pleurotus ostreatus PC15 OX=1137138 GN=PLEOSDRAFT_1087973 PE=4 SV=1	4
225	tr|A0A067NFS2|A0A067NFS2_PLEOS	Ribosomal protein L37 OS=Pleurotus ostreatus PC15 OX=1137138 GN=PLEOSDRAFT_1066156 PE=3 SV=1	4

226	tr|A0A067NG89|A0A067NG89_PLEOS	Extracellular metalloproteinase OS=Pleurotus ostreatus PC15 OX=1137138 GN=PLEOSDRAFT_1109874 PE=3 SV=1	3
227	tr|A0A067NLD9|A0A067NLD9_PLEOS	Transket_pyr domain-containing protein OS=Pleurotus ostreatus PC15 OX=1137138 GN=PLEOSDRAFT_1113999 PE=4 SV=1	3
228	tr|A0A067NJZ0|A0A067NJZ0_PLEOS	Uncharacterized protein OS=Pleurotus ostreatus PC15 OX=1137138 GN=PLEOSDRAFT_1089682 PE=3 SV=1	6
229	tr|A0A067NY20|A0A067NY20_PLEOS	S-(hydroxymethyl)glutathione dehydrogenase OS=Pleurotus ostreatus PC15 OX=1137138 GN=PLEOSDRAFT_1063808 PE=3 SV=1	3
230	tr|B1Q4S7|B1Q4S7_PLEOS	Ribonuclease T1 OS=Pleurotus ostreatus OX=5322 GN=RNPo1 PE=2 SV=1	5
230	tr|A0A067NSU8|A0A067NSU8_PLEOS	Uncharacterized protein OS=Pleurotus ostreatus PC15 OX=1137138 GN=PLEOSDRAFT_1088612 PE=4 SV=1	5
230	sp|P81762|RNPO_PLEOS	Guanyl-specific ribonuclease Po1 OS=Pleurotus ostreatus OX=5322 PE=1 SV=1	4
231	tr|A0A067NBS5|A0A067NBS5_PLEOS	ACB domain-containing protein OS=Pleurotus ostreatus PC15 OX=1137138 GN=PLEOSDRAFT_1090291 PE=4 SV=1	2
232	tr|A0A067NSH1|A0A067NSH1_PLEOS	DUF2235 domain-containing protein OS=Pleurotus ostreatus PC15 OX=1137138 GN=PLEOSDRAFT_1062816 PE=4 SV=1	3
233	tr|A0A067NCV3|A0A067NCV3_PLEOS	PPM-type phosphatase domain-containing protein OS=Pleurotus ostreatus PC15 OX=1137138 GN=PLEOSDRAFT_40905 PE=3 SV=1	3
234	tr|A0A067NX03|A0A067NX03_PLEOS	Ribosomal_L28e domain-containing protein OS=Pleurotus ostreatus PC15 OX=1137138 GN=PLEOSDRAFT_1096304	2

		PE=3 SV=1	
235	tr|A0A067NSE6|A0A067NSE6_PLEOS	Ribosomal protein L19 OS=Pleurotus ostreatus PC15 OX=1137138 GN=PLEOSDRAFT_1088261 PE=3 SV=1	4
236	tr|A0A067NWB7|A0A067NWB7_PLEOS	RRM domain-containing protein OS=Pleurotus ostreatus PC15 OX=1137138 GN=PLEOSDRAFT_1089506 PE=4 SV=1	12
237	tr|A0A067NF75|A0A067NF75_PLEOS	Redoxin domain-containing protein OS=Pleurotus ostreatus PC15 OX=1137138 GN=PLEOSDRAFT_1051082 PE=3 SV=1	6
238	tr|A0A067NH17|A0A067NH17_PLEOS	Uncharacterized protein OS=Pleurotus ostreatus PC15 OX=1137138 GN=PLEOSDRAFT_1068404 PE=3 SV=1	4
239	tr|A0A067NKI6|A0A067NKI6_PLEOS	Eukaryotic translation initiation factor 5A OS=Pleurotus ostreatus PC15 OX=1137138 GN=PLEOSDRAFT_1072569 PE=3 SV=1	5
240	tr|A0A067NAX7|A0A067NAX7_PLEOS	Inosine-5'-monophosphate dehydrogenase OS=Pleurotus ostreatus PC15 OX=1137138 GN=PLEOSDRAFT_1090677 PE=3 SV=1	3
241	tr|A0A067P203|A0A067P203_PLEOS	Uncharacterized protein OS=Pleurotus ostreatus PC15 OX=1137138 GN=PLEOSDRAFT_1061762 PE=4 SV=1	3
242	tr|D2JY85|D2JY85_PLEOS	Histone H2A OS=Pleurotus ostreatus OX=5322 PE=2 SV=1	6
242	tr|A0A067NVU4|A0A067NVU4_PLEOS	Histone H2A (Fragment) OS=Pleurotus ostreatus PC15 OX=1137138 GN=PLEOSDRAFT_1016318 PE=3 SV=1	6
242	tr|A0A067P7W0|A0A067P7W0_PLEOS	Histone H2A OS=Pleurotus ostreatus PC15 OX=1137138 GN=PLEOSDRAFT_1062070 PE=3 SV=1	6
242	tr|A0A067NY26|A0A067NY26_PLEOS	Histone H2A (Fragment) OS=Pleurotus ostreatus PC15 OX=1137138 GN=PLEOSDRAFT_354 PE=3 SV=1	6

242	tr|A0A067NG34|A0A067NG34_PLEOS	Histone H2A (Fragment) OS=Pleurotus ostreatus PC15 OX=1137138 GN=PLEOSDRAFT_1043832 PE=3 SV=1	6
243	tr|A0A067NEB9|A0A067NEB9_PLEOS	Uncharacterized protein OS=Pleurotus ostreatus PC15 OX=1137138 GN=PLEOSDRAFT_1093651 PE=4 SV=1	4
244	tr|A0A067NMF2|A0A067NMF2_PLEOS	Uncharacterized protein OS=Pleurotus ostreatus PC15 OX=1137138 GN=PLEOSDRAFT_185903 PE=3 SV=1	3
245	tr|A0A067N2Q3|A0A067N2Q3_PLEOS	Flavodoxin-like domain-containing protein OS=Pleurotus ostreatus PC15 OX=1137138 GN=PLEOSDRAFT_41896 PE=3 SV=1	3
245	tr|D2JY75|D2JY75_PLEOS	Flavodoxin-like domain-containing protein OS=Pleurotus ostreatus OX=5322 PE=2 SV=1	3
246	tr|A0A067PCH8|A0A067PCH8_PLEOS	Aldo_ket_red domain-containing protein OS=Pleurotus ostreatus PC15 OX=1137138 GN=PLEOSDRAFT_1061253 PE=4 SV=1	4
247	tr|D2JY67|D2JY67_PLEOS	Uncharacterized protein OS=Pleurotus ostreatus OX=5322 PE=2 SV=1	3
247	tr|A0A067NIW3|A0A067NIW3_PLEOS	Uncharacterized protein OS=Pleurotus ostreatus PC15 OX=1137138 GN=PLEOSDRAFT_1089545 PE=4 SV=1	3
248	tr|A0A067NCB2|A0A067NCB2_PLEOS	Ribosomal_S10 domain-containing protein OS=Pleurotus ostreatus PC15 OX=1137138 GN=PLEOSDRAFT_1113860 PE=3 SV=1	4
249	tr|A0A067NC11|A0A067NC11_PLEOS	Ribosomal_L23eN domain-containing protein (Fragment) OS=Pleurotus ostreatus PC15 OX=1137138 GN=PLEOSDRAFT_1025128 PE=3 SV=1	5
250	tr|A0A067NR30|A0A067NR30_PLEOS	Uncharacterized protein OS=Pleurotus ostreatus PC15 OX=1137138 GN=PLEOSDRAFT_1089130 PE=3 SV=1	3
251	tr|A0A067N8G3|A0A067N8G3_PLEOS	Uncharacterized protein OS=Pleurotus ostreatus PC15 OX=1137138	2

		GN=PLEOSDRAFT_1108891 PE=3 SV=1	
252	tr|A0A067NYX2|A0A067NYX2_PLEOS	Pyruvate dehydrogenase E1 component subunit alpha OS=Pleurotus ostreatus PC15 OX=1137138 GN=PLEOSDRAFT_1091334 PE=4 SV=1	2
253	tr|A0A067NAU1|A0A067NAU1_PLEOS	Uncharacterized protein OS=Pleurotus ostreatus PC15 OX=1137138 GN=PLEOSDRAFT_1114221 PE=4 SV=1	3
254	tr|A0A067NRJ6|A0A067NRJ6_PLEOS	Uncharacterized protein OS=Pleurotus ostreatus PC15 OX=1137138 GN=PLEOSDRAFT_1062678 PE=4 SV=1	3
255	tr|A0A067NC76|A0A067NC76_PLEOS	Obg-like ATPase 1 OS=Pleurotus ostreatus PC15 OX=1137138 GN=PLEOSDRAFT_1046650 PE=3 SV=1	3
256	tr|A0A067P3D8|A0A067P3D8_PLEOS	CS domain-containing protein OS=Pleurotus ostreatus PC15 OX=1137138 GN=PLEOSDRAFT_1088350 PE=4 SV=1	2
257	tr|A0A067NFS5|A0A067NFS5_PLEOS	Uncharacterized protein OS=Pleurotus ostreatus PC15 OX=1137138 GN=PLEOSDRAFT_1066159 PE=3 SV=1	2
258	tr|A0A067PDA2|A0A067PDA2_PLEOS	Uncharacterized protein OS=Pleurotus ostreatus PC15 OX=1137138 GN=PLEOSDRAFT_1087954 PE=4 SV=1	3
259	tr|A0A067NUN3|A0A067NUN3_PLEOS	Plasma membrane ATPase OS=Pleurotus ostreatus PC15 OX=1137138 GN=PLEOSDRAFT_1110948 PE=3 SV=1	3
260	tr|A0A067P007|A0A067P007_PLEOS	PABS domain-containing protein OS=Pleurotus ostreatus PC15 OX=1137138 GN=PLEOSDRAFT_1091586 PE=3 SV=1	2
261	tr|A0A067NCR3|A0A067NCR3_PLEOS	Alkaline phosphatase OS=Pleurotus ostreatus PC15 OX=1137138 GN=PLEOSDRAFT_46323 PE=3 SV=1	4
262	tr|A0A067NNA2|A0A067NNA2_PLEOS	PKS_ER domain-containing protein OS=Pleurotus ostreatus PC15 OX=1137138 GN=PLEOSDRAFT_1097157	2

		PE=4 SV=1	
263	tr|A0A067NKF3|A0A067NKF3_PLEOS	Citrate synthase OS=Pleurotus ostreatus PC15 OX=1137138 GN=PLEOSDRAFT_1067387 PE=3 SV=1	4
264	tr|A0A067NAM1|A0A067NAM1_PLEOS	Aldo_ket_red domain-containing protein OS=Pleurotus ostreatus PC15 OX=1137138 GN=PLEOSDRAFT_34588 PE=4 SV=1	2
265	tr|A0A067NX31|A0A067NX31_PLEOS	Uncharacterized protein OS=Pleurotus ostreatus PC15 OX=1137138 GN=PLEOSDRAFT_1051587 PE=3 SV=1	2
266	tr|A0A067PBJ0|A0A067PBJ0_PLEOS	Uncharacterized protein OS=Pleurotus ostreatus PC15 OX=1137138 GN=PLEOSDRAFT_1060916 PE=3 SV=1	4
267	tr|A0A067NI04|A0A067NI04_PLEOS	Uncharacterized protein OS=Pleurotus ostreatus PC15 OX=1137138 GN=PLEOSDRAFT_1068398 PE=4 SV=1	3
268	tr|A0A067NIL8|A0A067NIL8_PLEOS	Uncharacterized protein OS=Pleurotus ostreatus PC15 OX=1137138 GN=PLEOSDRAFT_61715 PE=3 SV=1	2
269	tr|A0A067N954|A0A067N954_PLEOS	Uncharacterized protein OS=Pleurotus ostreatus PC15 OX=1137138 GN=PLEOSDRAFT_1114460 PE=4 SV=1	2
270	tr|A0A067P1L4|A0A067P1L4_PLEOS	Uncharacterized protein OS=Pleurotus ostreatus PC15 OX=1137138 GN=PLEOSDRAFT_1110827 PE=3 SV=1	4
271	tr|A0A067NM95|A0A067NM95_PLEOS	Uncharacterized protein OS=Pleurotus ostreatus PC15 OX=1137138 GN=PLEOSDRAFT_1089609 PE=4 SV=1	3
272	tr|A0A067NUU3|A0A067NUU3_PLEOS	40S ribosomal protein S6 OS=Pleurotus ostreatus PC15 OX=1137138 GN=PLEOSDRAFT_1053014 PE=3 SV=1	2
273	tr|A0A067NAJ5|A0A067NAJ5_PLEOS	Uncharacterized protein OS=Pleurotus ostreatus PC15 OX=1137138 GN=PLEOSDRAFT_1094451 PE=4 SV=1	3
274	tr|A0A067N561|A0A067N561_PLEOS	Uncharacterized protein OS=Pleurotus ostreatus PC15 OX=1137138	2

		GN=PLEOSDRAFT_1090748 PE=4 SV=1	
275	tr|A0A067PDM5|A0A067PDM5_PLEOS	Uncharacterized protein OS=Pleurotus ostreatus PC15 OX=1137138 GN=PLEOSDRAFT_60644 PE=3 SV=1	2
276	tr|A0A067NUP7|A0A067NUP7_PLEOS	Aspartate aminotransferase OS=Pleurotus ostreatus PC15 OX=1137138 GN=PLEOSDRAFT_1088517 PE=3 SV=1	2
277	tr|Q96TW0|Q96TW0_PLEOS	Putative arginase (Fragment) OS=Pleurotus ostreatus OX=5322 GN=arg1 PE=2 SV=1	4
277	tr|A0A067NZQ5|A0A067NZQ5_PLEOS	Arginase OS=Pleurotus ostreatus PC15 OX=1137138 GN=PLEOSDRAFT_1087775 PE=3 SV=1	4
278	tr|A0A067NWC9|A0A067NWC9_PLEOS	Uncharacterized protein OS=Pleurotus ostreatus PC15 OX=1137138 GN=PLEOSDRAFT_1089520 PE=3 SV=1	4
279	tr|A0A067NF07|A0A067NF07_PLEOS	Peptidylprolyl isomerase OS=Pleurotus ostreatus PC15 OX=1137138 GN=PLEOSDRAFT_1057861 PE=4 SV=1	2
280	tr|A0A067N3J5|A0A067N3J5_PLEOS	Serine hydroxymethyltransferase OS=Pleurotus ostreatus PC15 OX=1137138 GN=PLEOSDRAFT_1059932 PE=3 SV=1	2
281	tr|A0A067NXD6|A0A067NXD6_PLEOS	Uncharacterized protein OS=Pleurotus ostreatus PC15 OX=1137138 GN=PLEOSDRAFT_1093558 PE=4 SV=1	3
282	tr|A0A067NP30|A0A067NP30_PLEOS	Peptidase S53 domain-containing protein OS=Pleurotus ostreatus PC15 OX=1137138 GN=PLEOSDRAFT_1039782 PE=4 SV=1	2
283	tr|Q96TV5|Q96TV5_PLEOS	Putative ribosomal protein S19 (Fragment) OS=Pleurotus ostreatus OX=5322 GN=rps19 PE=2 SV=1	3
283	tr|A0A067NT54|A0A067NT54_PLEOS	Uncharacterized protein OS=Pleurotus ostreatus PC15 OX=1137138 GN=PLEOSDRAFT_1070466 PE=3 SV=1	3

284	tr|A0A067P7P3|A0A067P7P3_PLEOS	Nascent polypeptide-associated complex subunit beta OS=Pleurotus ostreatus PC15 OX=1137138 GN=PLEOSDRAFT_1091873 PE=3 SV=1	2
285	tr|A0A067P5K9|A0A067P5K9_PLEOS	Lysine--tRNA ligase OS=Pleurotus ostreatus PC15 OX=1137138 GN=PLEOSDRAFT_1061805 PE=3 SV=1	2
286	tr|A0A067NX86|A0A067NX86_PLEOS	Uncharacterized protein OS=Pleurotus ostreatus PC15 OX=1137138 GN=PLEOSDRAFT_1089639 PE=3 SV=1	2
287	tr|A0A067NV79|A0A067NV79_PLEOS	Proteasome subunit alpha type OS=Pleurotus ostreatus PC15 OX=1137138 GN=PLEOSDRAFT_1083621 PE=3 SV=1	2
288	tr|A0A067NSA2|A0A067NSA2_PLEOS	Ribosomal_L7Ae domain-containing protein OS=Pleurotus ostreatus PC15 OX=1137138 GN=PLEOSDRAFT_1043668 PE=3 SV=1	2
289	tr|A0A067NL12|A0A067NL12_PLEOS	Carbohydrate esterase family 4 protein OS=Pleurotus ostreatus PC15 OX=1137138 GN=PLEOSDRAFT_1067159 PE=4 SV=1	2
289	tr|A0A067NJF8|A0A067NJF8_PLEOS	Carbohydrate esterase family 4 protein OS=Pleurotus ostreatus PC15 OX=1137138 GN=PLEOSDRAFT_1078467 PE=4 SV=1	1
289	tr|A0A067NGD7|A0A067NGD7_PLEOS	Carbohydrate esterase family 4 protein OS=Pleurotus ostreatus PC15 OX=1137138 GN=PLEOSDRAFT_1106007 PE=4 SV=1	1
289	tr|A0A067N8C4|A0A067N8C4_PLEOS	Carbohydrate esterase family 4 protein OS=Pleurotus ostreatus PC15 OX=1137138 GN=PLEOSDRAFT_1113735 PE=4 SV=1	1
290	tr|A0A067NEH1|A0A067NEH1_PLEOS	ATP synthase subunit 4, mitochondrial OS=Pleurotus ostreatus PC15 OX=1137138 GN=ATP4 PE=4 SV=1	2
291	tr|A0A067N6M3|A0A067N6M3_PLEOS	MFS domain-containing protein	2

		OS=Pleurotus ostreatus PC15 OX=1137138 GN=PLEOSDRAFT_1095022 PE=3 SV=1	
292	tr|A0A067P4I9|A0A067P4I9_PLEOS	40S ribosomal protein S24 OS=Pleurotus ostreatus PC15 OX=1137138 GN=PLEOSDRAFT_1053942 PE=3 SV=1	2
293	tr|A0A067NYE3|A0A067NYE3_PLEOS	Superoxide dismutase OS=Pleurotus ostreatus PC15 OX=1137138 GN=PLEOSDRAFT_1091329 PE=3 SV=1	2
293	tr|V5MZX5|V5MZX5_PLEOS	Superoxide dismutase OS=Pleurotus ostreatus OX=5322 PE=2 SV=1	1
293	tr|V5MZR6|V5MZR6_PLEOS	Superoxide dismutase OS=Pleurotus ostreatus OX=5322 PE=3 SV=1	1
293	tr|A0A482GME7|A0A482GME7_PLEOS	Superoxide dismutase OS=Pleurotus ostreatus OX=5322 PE=2 SV=1	1
294	tr|A0A067PAB4|A0A067PAB4_PLEOS	HMA domain-containing protein OS=Pleurotus ostreatus PC15 OX=1137138 GN=PLEOSDRAFT_1110514 PE=4 SV=1	2
295	tr|A0A067NU13|A0A067NU13_PLEOS	Uncharacterized protein OS=Pleurotus ostreatus PC15 OX=1137138 GN=PLEOSDRAFT_1089399 PE=4 SV=1	4
295	sp|Q7M4T6|PIA1_PLEOS	Serine proteinase inhibitor IA-1 OS=Pleurotus ostreatus OX=5322 PE=1 SV=1	2
295	sp|Q7M4T5|PIA2_PLEOS	Serine proteinase inhibitor IA-2 OS=Pleurotus ostreatus OX=5322 PE=1 SV=1	2
296	tr|A0A067P4E6|A0A067P4E6_PLEOS	DLH domain-containing protein OS=Pleurotus ostreatus PC15 OX=1137138 GN=PLEOSDRAFT_1088515 PE=4 SV=1	3
297	tr|A0A067NDL4|A0A067NDL4_PLEOS	Actin-depolymerizing factor 1 OS=Pleurotus ostreatus PC15 OX=1137138 GN=PLEOSDRAFT_1058112 PE=3 SV=1	3
298	tr|A0A067NN60|A0A067NN60_PLEOS	26S proteasome regulatory subunit	3

		RPN11 OS=Pleurotus ostreatus PC15 OX=1137138 GN=PLEOSDRAFT_1039215 PE=3 SV=1	
299	tr|A0A067PCL4|A0A067PCL4_PLEOS	Glutamate decarboxylase (Fragment) OS=Pleurotus ostreatus PC15 OX=1137138 GN=PLEOSDRAFT_42493 PE=3 SV=1	2
300	tr|A0A067PA11|A0A067PA11_PLEOS	Uncharacterized protein OS=Pleurotus ostreatus PC15 OX=1137138 GN=PLEOSDRAFT_1052108 PE=4 SV=1	3
301	tr|A0A067NC52|A0A067NC52_PLEOS	Transket_pyr domain-containing protein OS=Pleurotus ostreatus PC15 OX=1137138 GN=PLEOSDRAFT_1077982 PE=3 SV=1	3
302	tr|A0A067NJR9|A0A067NJR9_PLEOS	Uncharacterized protein OS=Pleurotus ostreatus PC15 OX=1137138 GN=PLEOSDRAFT_1090600 PE=3 SV=1	2
303	tr|A0A067NVZ8|A0A067NVZ8_PLEOS	BTB domain-containing protein (Fragment) OS=Pleurotus ostreatus PC15 OX=1137138 GN=PLEOSDRAFT_1014342 PE=4 SV=1	3
304	tr|A0A067NMW7|A0A067NMW7_PLEOS	ATP synthase subunit d, mitochondrial OS=Pleurotus ostreatus PC15 OX=1137138 GN=ATP7 PE=3 SV=1	2
305	tr|A0A067P3F5|A0A067P3F5_PLEOS	AB hydrolase-1 domain-containing protein OS=Pleurotus ostreatus PC15 OX=1137138 GN=PLEOSDRAFT_1111604 PE=4 SV=1	2
305	tr|A0A067P4W5|A0A067P4W5_PLEOS	AB hydrolase-1 domain-containing protein OS=Pleurotus ostreatus PC15 OX=1137138 GN=PLEOSDRAFT_1101897 PE=4 SV=1	1
306	tr|A0A067NS12|A0A067NS12_PLEOS	HABP4_PAI-RBP1 domain-containing protein OS=Pleurotus ostreatus PC15 OX=1137138 GN=PLEOSDRAFT_1111477 PE=4 SV=1	3
307	tr|A0A067NGK8|A0A067NGK8_PLEOS	Uncharacterized protein OS=Pleurotus ostreatus PC15 OX=1137138	2

		GN=PLEOSDRAFT_1049340 PE=4 SV=1	
308	tr|A0A067NJF4|A0A067NJF4_PLEOS	Glucosamine-6-phosphate isomerase OS=Pleurotus ostreatus PC15 OX=1137138 GN=PLEOSDRAFT_1096996 PE=3 SV=1	5
308	tr|A0A6B9ETI2|A0A6B9ETI2_PLEOS	Zinc cluster transcription factor 32 (Fragment) OS=Pleurotus ostreatus OX=5322 PE=2 SV=1	1
309	tr|A0A067NBL2|A0A067NBL2_PLEOS	60S ribosomal protein L41 OS=Pleurotus ostreatus PC15 OX=1137138 GN=PLEOSDRAFT_45869 PE=3 SV=1	2
310	tr|A0A067P1S4|A0A067P1S4_PLEOS	Aspartate aminotransferase OS=Pleurotus ostreatus PC15 OX=1137138 GN=PLEOSDRAFT_1052049 PE=3 SV=1	2
311	tr|A0A067NUQ4|A0A067NUQ4_PLEOS	UV excision repair protein RAD23 OS=Pleurotus ostreatus PC15 OX=1137138 GN=PLEOSDRAFT_1041327 PE=3 SV=1	4
312	tr|A0A067NSI3|A0A067NSI3_PLEOS	Uncharacterized protein OS=Pleurotus ostreatus PC15 OX=1137138 GN=PLEOSDRAFT_1062835 PE=3 SV=1	3
313	tr|A0A067P1D3|A0A067P1D3_PLEOS	Septin-type G domain-containing protein OS=Pleurotus ostreatus PC15 OX=1137138 GN=PLEOSDRAFT_1051920 PE=3 SV=1	2
314	tr|A0A067NCV7|A0A067NCV7_PLEOS	Vacuolar proton pump subunit B OS=Pleurotus ostreatus PC15 OX=1137138 GN=PLEOSDRAFT_1090158 PE=3 SV=1	3
315	tr|A0A067NEA3|A0A067NEA3_PLEOS	Uncharacterized protein OS=Pleurotus ostreatus PC15 OX=1137138 GN=PLEOSDRAFT_1089736 PE=4 SV=1	2
316	tr|A0A067NLK0|A0A067NLK0_PLEOS	Uncharacterized protein OS=Pleurotus ostreatus PC15 OX=1137138 GN=PLEOSDRAFT_1090331 PE=3 SV=1	2
317	tr|A0A067NZQ8|A0A067NZQ8_PLEOS	Alanine--tRNA ligase OS=Pleurotus ostreatus PC15 OX=1137138 GN=ALA1	2

		PE=3 SV=1	
318	tr|A0A067NVM8|A0A067NVM8_PLEOS	Seryl-tRNA synthetase OS=Pleurotus ostreatus PC15 OX=1137138 GN=PLEOSDRAFT_1088157 PE=4 SV=1	3
319	tr|A0A067NIR7|A0A067NIR7_PLEOS	Endoplasmic reticulum transmembrane protein OS=Pleurotus ostreatus PC15 OX=1137138 GN=PLEOSDRAFT_1067999 PE=3 SV=1	2
320	tr|A0A067P443|A0A067P443_PLEOS	Uncharacterized protein OS=Pleurotus ostreatus PC15 OX=1137138 GN=PLEOSDRAFT_1088007 PE=4 SV=1	2
321	tr|A0A067NKK5|A0A067NKK5_PLEOS	CCT-theta OS=Pleurotus ostreatus PC15 OX=1137138 GN=PLEOSDRAFT_1056867 PE=3 SV=1	2
322	tr|A0A067P2W0|A0A067P2W0_PLEOS	Uncharacterized protein OS=Pleurotus ostreatus PC15 OX=1137138 GN=PLEOSDRAFT_1062711 PE=3 SV=1	3
323	tr|A0A067NQ72|A0A067NQ72_PLEOS	Protein kinase domain-containing protein OS=Pleurotus ostreatus PC15 OX=1137138 GN=PLEOSDRAFT_61429 PE=4 SV=1	3
324	tr|A0A067PB82|A0A067PB82_PLEOS	PCI domain-containing protein OS=Pleurotus ostreatus PC15 OX=1137138 GN=PLEOSDRAFT_1060814 PE=3 SV=1	2
325	tr|A0A067NZ02|A0A067NZ02_PLEOS	Adenylyl cyclase-associated protein OS=Pleurotus ostreatus PC15 OX=1137138 GN=PLEOSDRAFT_1052012 PE=3 SV=1	2
326	tr|A0A067NV95|A0A067NV95_PLEOS	LCCL domain-containing protein OS=Pleurotus ostreatus PC15 OX=1137138 GN=PLEOSDRAFT_1054079 PE=4 SV=1	2
327	tr|A0A067NRR6|A0A067NRR6_PLEOS	Uncharacterized protein OS=Pleurotus ostreatus PC15 OX=1137138 GN=PLEOSDRAFT_1089864 PE=3 SV=1	5
328	tr|A0A067NAA2|A0A067NAA2_PLEOS	Uncharacterized protein OS=Pleurotus ostreatus PC15 OX=1137138	4

		GN=PLEOSDRAFT_1097837 PE=4 SV=1	
329	tr|A0A067NFD3|A0A067NFD3_PLEOS	Dipeptidyl-peptidase V OS=Pleurotus ostreatus PC15 OX=1137138 GN=PLEOSDRAFT_1050747 PE=3 SV=1	3
329	tr|A0A077K7X8|A0A077K7X8_PLEER	Dipeptidyl-peptidase V OS=Pleurotus eryngii OX=5323 GN=s9ap PE=3 SV=1	2
330	tr|A0A067NZH2|A0A067NZH2_PLEOS	T-complex protein 1 subunit delta OS=Pleurotus ostreatus PC15 OX=1137138 GN=PLEOSDRAFT_1061134 PE=3 SV=1	2
331	tr|A0A067N5Y6|A0A067N5Y6_PLEOS	Uncharacterized protein OS=Pleurotus ostreatus PC15 OX=1137138 GN=PLEOSDRAFT_1098210 PE=4 SV=1	2
332	tr|A0A067NPY7|A0A067NPY7_PLEOS	RanBD1 domain-containing protein OS=Pleurotus ostreatus PC15 OX=1137138 GN=PLEOSDRAFT_1039394 PE=4 SV=1	4
333	tr|A0A067NIB4|A0A067NIB4_PLEOS	DLH domain-containing protein OS=Pleurotus ostreatus PC15 OX=1137138 GN=PLEOSDRAFT_1105627 PE=4 SV=1	3
334	tr|A0A067NFJ2|A0A067NFJ2_PLEOS	Thiamine thiazole synthase OS=Pleurotus ostreatus PC15 OX=1137138 GN=PLEOSDRAFT_1057999 PE=3 SV=1	2
335	tr|A0A067NZ20|A0A067NZ20_PLEOS	Uncharacterized protein OS=Pleurotus ostreatus PC15 OX=1137138 GN=PLEOSDRAFT_1088906 PE=3 SV=1	5
336	tr|A0A067NPK7|A0A067NPK7_PLEOS	Isocitrate lyase OS=Pleurotus ostreatus PC15 OX=1137138 GN=PLEOSDRAFT_1067024 PE=3 SV=1	1
337	tr|A0A067P1V4|A0A067P1V4_PLEOS	Citrate synthase OS=Pleurotus ostreatus PC15 OX=1137138 GN=PLEOSDRAFT_1110467 PE=3 SV=1	1
338	tr|A0A067NVH0|A0A067NVH0_PLEOS	Uncharacterized protein OS=Pleurotus ostreatus PC15 OX=1137138 GN=PLEOSDRAFT_1088203 PE=4 SV=1	4
339	tr|A0A067NXA6|A0A067NXA6_PLEOS	Uncharacterized protein OS=Pleurotus	1

		ostreatus PC15 OX=1137138 GN=PLEOSDRAFT_156494 PE=4 SV=1	
340	tr|A0A067P6M0|A0A067P6M0_PLEOS	CYTOSOL_AP domain-containing protein OS=Pleurotus ostreatus PC15 OX=1137138 GN=PLEOSDRAFT_1075581 PE=3 SV=1	1
341	tr|A0A067NRL6|A0A067NRL6_PLEOS	Cytochrome b5 heme-binding domain-containing protein OS=Pleurotus ostreatus PC15 OX=1137138 GN=PLEOSDRAFT_1064455 PE=3 SV=1	2
342	tr|A0A067NGV7|A0A067NGV7_PLEOS	Prolyl-tRNA synthetase OS=Pleurotus ostreatus PC15 OX=1137138 GN=PLEOSDRAFT_1094905 PE=3 SV=1	1
343	tr|A0A067NA41|A0A067NA41_PLEOS	Uncharacterized protein OS=Pleurotus ostreatus PC15 OX=1137138 GN=PLEOSDRAFT_1067519 PE=3 SV=1	2
344	tr|A0A067P6N3|A0A067P6N3_PLEOS	Glutaredoxin domain-containing protein OS=Pleurotus ostreatus PC15 OX=1137138 GN=PLEOSDRAFT_1054608 PE=4 SV=1	2
345	tr|A0A067P286|A0A067P286_PLEOS	Succinate--CoA ligase [ADP-forming] subunit beta, mitochondrial OS=Pleurotus ostreatus PC15 OX=1137138 GN=PLEOSDRAFT_1095962 PE=3 SV=1	1
346	tr|A0A067NGT5|A0A067NGT5_PLEOS	Uncharacterized protein OS=Pleurotus ostreatus PC15 OX=1137138 GN=PLEOSDRAFT_1077820 PE=3 SV=1	1
347	tr|A0A067NNW3|A0A067NNW3_PLEOS	Uncharacterized protein (Fragment) OS=Pleurotus ostreatus PC15 OX=1137138 GN=PLEOSDRAFT_1054697 PE=3 SV=1	2
348	tr|A0A067P3B6|A0A067P3B6_PLEOS	Uncharacterized protein OS=Pleurotus ostreatus PC15 OX=1137138 GN=PLEOSDRAFT_1053579 PE=4 SV=1	1
349	tr|A0A067NC64|A0A067NC64_PLEOS	Valyl-tRNA synthetase OS=Pleurotus ostreatus PC15 OX=1137138 GN=PLEOSDRAFT_1094347 PE=3 SV=1	1

350	tr|A0A067NNF1|A0A067NNF1_PLEOS	Uncharacterized protein OS=Pleurotus ostreatus PC15 OX=1137138 GN=PLEOSDRAFT_1038297 PE=4 SV=1	1
351	tr|A0A067NS50|A0A067NS50_PLEOS	Importin subunit alpha OS=Pleurotus ostreatus PC15 OX=1137138 GN=PLEOSDRAFT_1036627 PE=3 SV=1	2
352	tr|A0A067P056|A0A067P056_PLEOS	Aldedh domain-containing protein OS=Pleurotus ostreatus PC15 OX=1137138 GN=PLEOSDRAFT_1092884 PE=3 SV=1	3
353	tr|A0A067NQ67|A0A067NQ67_PLEOS	Peptidase_S8 domain-containing protein OS=Pleurotus ostreatus PC15 OX=1137138 GN=PLEOSDRAFT_31354 PE=3 SV=1	1
354	tr|A0A067N6B7|A0A067N6B7_PLEOS	Uncharacterized protein OS=Pleurotus ostreatus PC15 OX=1137138 GN=PLEOSDRAFT_1068380 PE=3 SV=1	3
355	tr|A0A067P1P7|A0A067P1P7_PLEOS	NDUFV2, NADH dehydrogenase 24 kd subunit OS=Pleurotus ostreatus PC15 OX=1137138 GN=NDUFV2 PE=3 SV=1	1
356	tr|A0A067NTR2|A0A067NTR2_PLEOS	Glycoside hydrolase family 13 protein OS=Pleurotus ostreatus PC15 OX=1137138 GN=PLEOSDRAFT_61816 PE=4 SV=1	2
357	tr|A0A067NP31|A0A067NP31_PLEOS	Uncharacterized protein OS=Pleurotus ostreatus PC15 OX=1137138 GN=PLEOSDRAFT_160448 PE=3 SV=1	1
358	tr|A0A067NRK5|A0A067NRK5_PLEOS	Biotin carboxylase OS=Pleurotus ostreatus PC15 OX=1137138 GN=PLEOSDRAFT_51540 PE=4 SV=1	1
359	tr|A0A067PA83|A0A067PA83_PLEOS	40S ribosomal protein S30 OS=Pleurotus ostreatus PC15 OX=1137138 GN=PLEOSDRAFT_1087561 PE=3 SV=1	2
360	tr|A0A067NTN9|A0A067NTN9_PLEOS	Uncharacterized protein OS=Pleurotus ostreatus PC15 OX=1137138 GN=PLEOSDRAFT_1088347 PE=3 SV=1	1
361	tr|A0A067P416|A0A067P416_PLEOS	Adenylate kinase OS=Pleurotus ostreatus	2

		PC15 OX=1137138 GN=ADK1 PE=3 SV=1	
362	tr|A0A067NCS9|A0A067NCS9_PLEOS	Uncharacterized protein OS=Pleurotus ostreatus PC15 OX=1137138 GN=PLEOSDRAFT_1067547 PE=3 SV=1	2
363	tr|A0A067NLY5|A0A067NLY5_PLEOS	Ubiquitin-like modifier-activating enzyme ATG7 OS=Pleurotus ostreatus PC15 OX=1137138 GN=PLEOSDRAFT_1058604 PE=3 SV=1	1
364	tr|A0A067NIC8|A0A067NIC8_PLEOS	AMP-binding domain-containing protein OS=Pleurotus ostreatus PC15 OX=1137138 GN=PLEOSDRAFT_1059065 PE=4 SV=1	1
365	tr|A0A067N7H6|A0A067N7H6_PLEOS	Uncharacterized protein OS=Pleurotus ostreatus PC15 OX=1137138 GN=PLEOSDRAFT_1090596 PE=3 SV=1	3
366	tr|A0A067NES4|A0A067NES4_PLEOS	Uncharacterized protein OS=Pleurotus ostreatus PC15 OX=1137138 GN=PLEOSDRAFT_30410 PE=4 SV=1	1
367	tr|A0A067N9G3|A0A067N9G3_PLEOS	Cytochrome b5 heme-binding domain-containing protein OS=Pleurotus ostreatus PC15 OX=1137138 GN=PLEOSDRAFT_1067485 PE=3 SV=1	2
368	tr|A0A067NZS6|A0A067NZS6_PLEOS	Uncharacterized protein (Fragment) OS=Pleurotus ostreatus PC15 OX=1137138 GN=PLEOSDRAFT_1032586 PE=4 SV=1	2
368	tr|A0A067P6V5|A0A067P6V5_PLEOS	Uncharacterized protein OS=Pleurotus ostreatus PC15 OX=1137138 GN=PLEOSDRAFT_1110902 PE=4 SV=1	1
369	tr|A0A067NHB7|A0A067NHB7_PLEOS	Uncharacterized protein OS=Pleurotus ostreatus PC15 OX=1137138 GN=PLEOSDRAFT_1059697 PE=4 SV=1	1
370	tr|A0A067N530|A0A067N530_PLEOS	Uncharacterized protein (Fragment) OS=Pleurotus ostreatus PC15 OX=1137138 GN=PLEOSDRAFT_1011921 PE=3 SV=1	2
371	tr|A0A067NVN5|A0A067NVN5_PLEOS	W2 domain-containing protein OS=Pleurotus ostreatus PC15	1

		OX=1137138 GN=PLEOSDRAFT_1089622 PE=3 SV=1	
372	tr|A0A067NLN1|A0A067NLN1_PLEOS	Uncharacterized protein OS=Pleurotus ostreatus PC15 OX=1137138 GN=PLEOSDRAFT_1092760 PE=3 SV=1	1
373	tr|A0A067NH82|A0A067NH82_PLEOS	Uncharacterized protein OS=Pleurotus ostreatus PC15 OX=1137138 GN=PLEOSDRAFT_1090865 PE=3 SV=1	1
374	tr|A0A067P0G6|A0A067P0G6_PLEOS	Uncharacterized protein (Fragment) OS=Pleurotus ostreatus PC15 OX=1137138 GN=PLEOSDRAFT_1055810 PE=4 SV=1	1
375	tr|A0A067NZ85|A0A067NZ85_PLEOS	Uncharacterized protein OS=Pleurotus ostreatus PC15 OX=1137138 GN=PLEOSDRAFT_1089134 PE=4 SV=1	5
376	tr|A0A067N5Y3|A0A067N5Y3_PLEOS	Dihydrolipoyl dehydrogenase OS=Pleurotus ostreatus PC15 OX=1137138 GN=PLEOSDRAFT_62289 PE=3 SV=1	1
377	tr|A0A067NBE6|A0A067NBE6_PLEOS	cobW domain-containing protein OS=Pleurotus ostreatus PC15 OX=1137138 GN=PLEOSDRAFT_1057631 PE=3 SV=1	1
378	tr|A0A067NT88|A0A067NT88_PLEOS	Uncharacterized protein OS=Pleurotus ostreatus PC15 OX=1137138 GN=PLEOSDRAFT_1088671 PE=4 SV=1	1
379	tr|A0A067NK42|A0A067NK42_PLEOS	Uncharacterized protein OS=Pleurotus ostreatus PC15 OX=1137138 GN=PLEOSDRAFT_1096799 PE=3 SV=1	3
380	tr|A0A067PD94|A0A067PD94_PLEOS	Uncharacterized protein OS=Pleurotus ostreatus PC15 OX=1137138 GN=PLEOSDRAFT_1032174 PE=4 SV=1	1
381	tr|A0A067NN21|A0A067NN21_PLEOS	Uncharacterized protein OS=Pleurotus ostreatus PC15 OX=1137138 GN=PLEOSDRAFT_1046493 PE=4 SV=1	1
382	tr|A0A067N928|A0A067N928_PLEOS	Thioredoxin domain-containing protein OS=Pleurotus ostreatus PC15 OX=1137138 GN=PLEOSDRAFT_1059662	2

		PE=3 SV=1	
383	tr|A0A067NVK7|A0A067NVK7_PLEOS	Uncharacterized protein OS=Pleurotus ostreatus PC15 OX=1137138 GN=PLEOSDRAFT_1053360 PE=3 SV=1	2
384	tr|A0A067NRA4|A0A067NRA4_PLEOS	Saccharopine dehydrogenase [NAD(+), L-lysine-forming] OS=Pleurotus ostreatus PC15 OX=1137138 GN=PLEOSDRAFT_1092207 PE=3 SV=1	1
385	tr|A0A067NJ31|A0A067NJ31_PLEOS	Uncharacterized protein OS=Pleurotus ostreatus PC15 OX=1137138 GN=PLEOSDRAFT_1089575 PE=4 SV=1	1
386	tr|A0A067NCM1|A0A067NCM1_PLEOS	AAA domain-containing protein OS=Pleurotus ostreatus PC15 OX=1137138 GN=PLEOSDRAFT_1046070 PE=3 SV=1	1
387	tr|A0A067NF91|A0A067NF91_PLEOS	AAA domain-containing protein OS=Pleurotus ostreatus PC15 OX=1137138 GN=PLEOSDRAFT_1066016 PE=3 SV=1	1
388	tr|A0A067NJB2|A0A067NJB2_PLEOS	Jacalin-type lectin domain-containing protein OS=Pleurotus ostreatus PC15 OX=1137138 GN=PLEOSDRAFT_1097387 PE=4 SV=1	1
388	tr|A0A067NTY8|A0A067NTY8_PLEOS	Jacalin-type lectin domain-containing protein OS=Pleurotus ostreatus PC15 OX=1137138 GN=PLEOSDRAFT_1105974 PE=4 SV=1	1
389	tr|A0A067NI49|A0A067NI49_PLEOS	CHZ domain-containing protein OS=Pleurotus ostreatus PC15 OX=1137138 GN=PLEOSDRAFT_173886 PE=3 SV=1	1
390	tr|A0A067P5X3|A0A067P5X3_PLEOS	Uncharacterized protein (Fragment) OS=Pleurotus ostreatus PC15 OX=1137138 GN=PLEOSDRAFT_22618 PE=3 SV=1	1
391	tr|A0A067P1G9|A0A067P1G9_PLEOS	COX4 subunit of cytochrome c oxidase OS=Pleurotus ostreatus PC15 OX=1137138 GN=COX4 PE=4 SV=1	1

392	tr|A0A067NYD2|A0A067NYD2_PLEOS	Uncharacterized protein OS=Pleurotus ostreatus PC15 OX=1137138 GN=PLEOSDRAFT_1091324 PE=3 SV=1	1
393	tr|A0A067NTH7|A0A067NTH7_PLEOS	Peptidyl-prolyl cis-trans isomerase OS=Pleurotus ostreatus PC15 OX=1137138 GN=PLEOSDRAFT_1088739 PE=4 SV=1	1
394	tr|A0A067NQM0|A0A067NQM0_PLEOS	Uncharacterized protein OS=Pleurotus ostreatus PC15 OX=1137138 GN=PLEOSDRAFT_1111258 PE=3 SV=1	2
395	tr|A0A067NJ94|A0A067NJ94_PLEOS	Uncharacterized protein OS=Pleurotus ostreatus PC15 OX=1137138 GN=PLEOSDRAFT_1090255 PE=4 SV=1	2
396	tr|A0A067ND63|A0A067ND63_PLEOS	Cytochrome b5 heme-binding domain-containing protein OS=Pleurotus ostreatus PC15 OX=1137138 GN=PLEOSDRAFT_1047612 PE=3 SV=1	1
397	RRRRRtr|A0A067NT46|A0A067NT46_PLEOS	REVERSED Uncharacterized protein OS=Pleurotus ostreatus PC15 OX=1137138 GN=PLEOSDRAFT_1063034 PE=4 SV=1	1
398	tr|Q5W9E8|Q5W9E8_PLEOS	Pleurotolysin B OS=Pleurotus ostreatus OX=5322 GN=plyB PE=1 SV=1	16
398	tr|D0FZZ3|D0FZZ3_PLEER	Erylysin B OS=Pleurotus eryngii OX=5323 GN=eryB PE=2 SV=1	10
399	tr|A0A097IYG6|A0A097IYG6_PLEER	Serine proteinase OS=Pleurotus eryngii OX=5323 GN=Spr PE=2 SV=1	4
400	tr|A0A067P1D2|A0A067P1D2_PLEOS	Cell division control protein 42 homolog OS=Pleurotus ostreatus PC15 OX=1137138 GN=PLEOSDRAFT_1110796 PE=3 SV=1	3
401	tr|A0A067N5G6|A0A067N5G6_PLEOS	Uncharacterized protein (Fragment) OS=Pleurotus ostreatus PC15 OX=1137138 GN=PLEOSDRAFT_186159 PE=3 SV=1	2
402	tr|A0A067NME3|A0A067NME3_PLEOS	Serine/threonine-protein phosphatase OS=Pleurotus ostreatus PC15 OX=1137138 GN=PLEOSDRAFT_1067551	1

		PE=3 SV=1	
403	tr|A0A067NUN8|A0A067NUN8_PLEOS	ATP synthase subunit 5, mitochondrial OS=Pleurotus ostreatus PC15 OX=1137138 GN=ATP5 PE=3 SV=1	3
404	tr|A0A067NPL8|A0A067NPL8_PLEOS	CULLIN_2 domain-containing protein OS=Pleurotus ostreatus PC15 OX=1137138 GN=PLEOSDRAFT_1058212 PE=3 SV=1	1
405	tr|A0A067P946|A0A067P946_PLEOS	ATP synthase subunit gamma (Fragment) OS=Pleurotus ostreatus PC15 OX=1137138 GN=ATP3 PE=3 SV=1	1
406	tr|A0A067NZU3|A0A067NZU3_PLEOS	Tryptophan synthase OS=Pleurotus ostreatus PC15 OX=1137138 GN=PLEOSDRAFT_60756 PE=3 SV=1	1
407	tr|A0A067NZ22|A0A067NZ22_PLEOS	Iso_dh domain-containing protein OS=Pleurotus ostreatus PC15 OX=1137138 GN=PLEOSDRAFT_1064180 PE=3 SV=1	1
408	tr|A0A067NYB2|A0A067NYB2_PLEOS	Proteasome subunit alpha type OS=Pleurotus ostreatus PC15 OX=1137138 GN=PLEOSDRAFT_1039242 PE=3 SV=1	1
409	tr|A0A067NY97|A0A067NY97_PLEOS	Threonyl-tRNA synthetase OS=Pleurotus ostreatus PC15 OX=1137138 GN=PLEOSDRAFT_1032700 PE=3 SV=1	1
410	tr|A0A067NXW5|A0A067NXW5_PLEOS	ERF-3 OS=Pleurotus ostreatus PC15 OX=1137138 GN=PLEOSDRAFT_1054926 PE=3 SV=1	1
411	tr|A0A067NXG5|A0A067NXG5_PLEOS	Polysacc_synt_4 domain-containing protein OS=Pleurotus ostreatus PC15 OX=1137138 GN=PLEOSDRAFT_1051577 PE=4 SV=1	1
412	tr|A0A067NW13|A0A067NW13_PLEOS	NAD_binding_2 domain-containing protein OS=Pleurotus ostreatus PC15 OX=1137138 GN=PLEOSDRAFT_1037028 PE=4 SV=1	2
413	tr|A0A067NVN9|A0A067NVN9_PLEOS	Uncharacterized protein OS=Pleurotus ostreatus PC15 OX=1137138	1

		GN=PLEOSDRAFT_155740 PE=3 SV=1	
414	tr|A0A067NTY1|A0A067NTY1_PLEOS	Uncharacterized protein (Fragment) OS=Pleurotus ostreatus PC15 OX=1137138 GN=PLEOSDRAFT_1057539 PE=4 SV=1	2
415	tr|A0A067NRD1|A0A067NRD1_PLEOS	Cytochrome b-c1 complex subunit Rieske, mitochondrial OS=Pleurotus ostreatus PC15 OX=1137138 GN=PLEOSDRAFT_1088400 PE=3 SV=1	1
416	tr|A0A067NR85|A0A067NR85_PLEOS	Uncharacterized protein OS=Pleurotus ostreatus PC15 OX=1137138 GN=PLEOSDRAFT_1070108 PE=4 SV=1	1
417	tr|A0A067NCX5|A0A067NCX5_PLEOS	Uncharacterized protein OS=Pleurotus ostreatus PC15 OX=1137138 GN=PLEOSDRAFT_52286 PE=4 SV=1	2
418	tr|A0A067N8X6|A0A067N8X6_PLEOS	Uncharacterized protein OS=Pleurotus ostreatus PC15 OX=1137138 GN=PLEOSDRAFT_1107407 PE=4 SV=1	1
419	RRRRRtr|A0A067N7U4|A0A067N7U4_PLEOS	REVERSED t-SNARE coiled-coil homology domain-containing protein OS=Pleurotus ostreatus PC15 OX=1137138 GN=PLEOSDRAFT_1090707 PE=3 SV=1	1
419	RRRRRtr|A0A067NYJ9|A0A067NYJ9_PLEOS	REVERSED t-SNARE coiled-coil homology domain-containing protein OS=Pleurotus ostreatus PC15 OX=1137138 GN=PLEOSDRAFT_1040365 PE=3 SV=1	1
420	tr|G8DA07|G8DA07_9AGAR	Beta-flanking protein (Fragment) OS=Pleurotus tuoliensis OX=879823 GN=beta-fg PE=4 SV=1	1
420	tr|G8DA03|G8DA03_9AGAR	Beta-flanking protein (Fragment) OS=Pleurotus tuoliensis OX=879823 GN=beta-fg PE=4 SV=1	1
420	tr|G8D9Z9|G8D9Z9_9AGAR	Beta-flanking protein (Fragment) OS=Pleurotus tuoliensis OX=879823 GN=beta-fg PE=4 SV=1	1
420	tr|A0A067P3S1|A0A067P3S1_PLEOS	Uncharacterized protein OS=Pleurotus ostreatus PC15 OX=1137138	1

		GN=PLEOSDRAFT_166368 PE=4 SV=1	
421	tr|A0A067P4G3|A0A067P4G3_PLEOS	Uncharacterized protein OS=Pleurotus ostreatus PC15 OX=1137138 GN=PLEOSDRAFT_166158 PE=4 SV=1	1
421	tr|A0A067NMC2|A0A067NMC2_PLEOS	Uncharacterized protein OS=Pleurotus ostreatus PC15 OX=1137138 GN=PLEOSDRAFT_161220 PE=4 SV=1	1
422	tr|A0A067P3X5|A0A067P3X5_PLEOS	Uncharacterized protein OS=Pleurotus ostreatus PC15 OX=1137138 GN=PLEOSDRAFT_1074047 PE=3 SV=1	1
423	tr|A0A067P3B8|A0A067P3B8_PLEOS	U6 snRNA-associated Sm-like protein LSm5 OS=Pleurotus ostreatus PC15 OX=1137138 GN=LSM5 PE=3 SV=1	2
424	tr|A0A067P2W3|A0A067P2W3_PLEOS	Importin N-terminal domain-containing protein OS=Pleurotus ostreatus PC15 OX=1137138 GN=PLEOSDRAFT_1091551 PE=3 SV=1	1
424	tr|A0A067NZT9|A0A067NZT9_PLEOS	Importin N-terminal domain-containing protein OS=Pleurotus ostreatus PC15 OX=1137138 GN=PLEOSDRAFT_172384 PE=3 SV=1	1
425	tr|A0A067P2I0|A0A067P2I0_PLEOS	GAE domain-containing protein OS=Pleurotus ostreatus PC15 OX=1137138 GN=PLEOSDRAFT_1053480 PE=4 SV=1	1
425	tr|A0A067NFG1|A0A067NFG1_PLEOS	AP-1 complex subunit gamma OS=Pleurotus ostreatus PC15 OX=1137138 GN=PLEOSDRAFT_1057262 PE=3 SV=1	1
426	tr|A0A067P193|A0A067P193_PLEOS	Protein AF-9 homolog OS=Pleurotus ostreatus PC15 OX=1137138 GN=YAF9 PE=3 SV=1	1
427	tr|A0A067P119|A0A067P119_PLEOS	HABP4_PAI-RBP1 domain-containing protein OS=Pleurotus ostreatus PC15 OX=1137138 GN=PLEOSDRAFT_1069268 PE=4 SV=1	1
428	tr|A0A067P0Z2|A0A067P0Z2_PLEOS	Uncharacterized protein OS=Pleurotus ostreatus PC15 OX=1137138	1

		GN=PLEOSDRAFT_1112326 PE=4 SV=1	
429	tr|A0A067NVR0|A0A067NVR0_PLEOS	Uncharacterized protein OS=Pleurotus ostreatus PC15 OX=1137138 GN=PLEOSDRAFT_1089630 PE=4 SV=1	1
430	tr|A0A067NV28|A0A067NV28_PLEOS	CVNH domain-containing protein (Fragment) OS=Pleurotus ostreatus PC15 OX=1137138 GN=PLEOSDRAFT_1091822 PE=4 SV=1	1
431	tr|A0A067NUY3|A0A067NUY3_PLEOS	Lactoylglutathione lyase OS=Pleurotus ostreatus PC15 OX=1137138 GN=PLEOSDRAFT_1112538 PE=3 SV=1	1
432	tr|A0A067NU90|A0A067NU90_PLEOS	6-phosphogluconolactonase OS=Pleurotus ostreatus PC15 OX=1137138 GN=PLEOSDRAFT_1062579 PE=3 SV=1	1
433	tr|A0A067NU03|A0A067NU03_PLEOS	Uncharacterized protein OS=Pleurotus ostreatus PC15 OX=1137138 GN=PLEOSDRAFT_1036222 PE=4 SV=1	1
434	tr|A0A067NTN6|A0A067NTN6_PLEOS	GLTP domain-containing protein OS=Pleurotus ostreatus PC15 OX=1137138 GN=PLEOSDRAFT_1093240 PE=4 SV=1	1
435	tr|A0A067NTG0|A0A067NTG0_PLEOS	Class E vacuolar protein-sorting machinery protein HSE1 OS=Pleurotus ostreatus PC15 OX=1137138 GN=PLEOSDRAFT_159193 PE=3 SV=1	1
436	tr|A0A067NT94|A0A067NT94_PLEOS	NIPSNAP domain-containing protein OS=Pleurotus ostreatus PC15 OX=1137138 GN=PLEOSDRAFT_1036108 PE=3 SV=1	1
437	tr|A0A067NSD9|A0A067NSD9_PLEOS	QCR6, subunit of the ubiquinol cytochrome-c reductase complex OS=Pleurotus ostreatus PC15 OX=1137138 GN=QCR6 PE=3 SV=1	1
438	tr|A0A067NS58|A0A067NS58_PLEOS	Clathrin heavy chain OS=Pleurotus ostreatus PC15 OX=1137138 GN=PLEOSDRAFT_1066219 PE=3 SV=1	1

439	tr|A0A067NS15|A0A067NS15_PLEOS	WD_REPEATS_REGION domain-containing protein OS=Pleurotus ostreatus PC15 OX=1137138 GN=PLEOSDRAFT_1105781 PE=3 SV=1	1
440	tr|A0A067NP81|A0A067NP81_PLEOS	F-box domain-containing protein OS=Pleurotus ostreatus PC15 OX=1137138 GN=PLEOSDRAFT_1112462 PE=4 SV=1	1
441	tr|A0A067NMT1|A0A067NMT1_PLEOS	DJ-1_PfpI domain-containing protein OS=Pleurotus ostreatus PC15 OX=1137138 GN=PLEOSDRAFT_1045544 PE=4 SV=1	1
442	tr|A0A067NLX0|A0A067NLX0_PLEOS	Iso_dh domain-containing protein OS=Pleurotus ostreatus PC15 OX=1137138 GN=PLEOSDRAFT_1038313 PE=3 SV=1	1
443	tr|A0A067NLD2|A0A067NLD2_PLEOS	CRAL-TRIO domain-containing protein OS=Pleurotus ostreatus PC15 OX=1137138 GN=PLEOSDRAFT_61366 PE=4 SV=1	1
444	tr|A0A067NL42|A0A067NL42_PLEOS	Uncharacterized protein OS=Pleurotus ostreatus PC15 OX=1137138 GN=PLEOSDRAFT_1102839 PE=3 SV=1	1
445	tr|A0A067NJ33|A0A067NJ33_PLEOS	Eukaryotic translation initiation factor 3 subunit E OS=Pleurotus ostreatus PC15 OX=1137138 GN=INT6 PE=3 SV=1	1
446	tr|A0A067NIZ8|A0A067NIZ8_PLEOS	Chitinase OS=Pleurotus ostreatus PC15 OX=1137138 GN=PLEOSDRAFT_1041965 PE=3 SV=1	1
447	tr|A0A067NIW9|A0A067NIW9_PLEOS	Sm domain-containing protein OS=Pleurotus ostreatus PC15 OX=1137138 GN=PLEOSDRAFT_1105826 PE=4 SV=1	1
447	tr|A0A067NE87|A0A067NE87_PLEOS	Sm domain-containing protein OS=Pleurotus ostreatus PC15 OX=1137138 GN=PLEOSDRAFT_1044754 PE=4 SV=1	1
448	tr|A0A067NI63|A0A067NI63_PLEOS	Uncharacterized protein OS=Pleurotus	1

		ostreatus PC15 OX=1137138 GN=PLEOSDRAFT_1109082 PE=4 SV=1	
449	tr|A0A067NI17|A0A067NI17_PLEOS	Protein phosphatase PP2A regulatory subunit B OS=Pleurotus ostreatus PC15 OX=1137138 GN=PLEOSDRAFT_1057132 PE=3 SV=1	1
450	tr|A0A067NGS6|A0A067NGS6_PLEOS	MFS domain-containing protein OS=Pleurotus ostreatus PC15 OX=1137138 GN=PLEOSDRAFT_1089962 PE=3 SV=1	1
451	tr|A0A067NGG8|A0A067NGG8_PLEOS	Uncharacterized protein OS=Pleurotus ostreatus PC15 OX=1137138 GN=PLEOSDRAFT_1089917 PE=4 SV=1	1
452	tr|A0A067NG73|A0A067NG73_PLEOS	5-aminoimidazole-4-carboxamide ribonucleotide formyltransferase OS=Pleurotus ostreatus PC15 OX=1137138 GN=PLEOSDRAFT_159040 PE=3 SV=1	1
453	tr|A0A067NFC6|A0A067NFC6_PLEOS	acidPPc domain-containing protein OS=Pleurotus ostreatus PC15 OX=1137138 GN=PLEOSDRAFT_1095079 PE=4 SV=1	1
454	tr|A0A067NEB1|A0A067NEB1_PLEOS	Lipase_3 domain-containing protein OS=Pleurotus ostreatus PC15 OX=1137138 GN=PLEOSDRAFT_1090000 PE=4 SV=1	1
455	tr|A0A067NE83|A0A067NE83_PLEOS	Uncharacterized protein OS=Pleurotus ostreatus PC15 OX=1137138 GN=PLEOSDRAFT_1090236 PE=4 SV=1	1
456	tr|A0A067NDF8|A0A067NDF8_PLEOS	Uncharacterized protein OS=Pleurotus ostreatus PC15 OX=1137138 GN=PLEOSDRAFT_1109280 PE=4 SV=1	1
457	tr|A0A067NBR1|A0A067NBR1_PLEOS	AB hydrolase-1 domain-containing protein OS=Pleurotus ostreatus PC15 OX=1137138 GN=PLEOSDRAFT_1078511 PE=4 SV=1	1
458	tr|A0A067N9K9|A0A067N9K9_PLEOS	Uncharacterized protein OS=Pleurotus ostreatus PC15 OX=1137138	1

		GN=PLEOSDRAFT_1094340 PE=4 SV=1	
459	tr|A0A067N8K5|A0A067N8K5_PLEOS	Uncharacterized protein OS=Pleurotus ostreatus PC15 OX=1137138 GN=PLEOSDRAFT_1090797 PE=4 SV=1	1
460	tr|A0A067N7K3|A0A067N7K3_PLEOS	GMC_OxRdtase_N domain-containing protein OS=Pleurotus ostreatus PC15 OX=1137138 GN=PLEOSDRAFT_1114195 PE=3 SV=1	1
461	tr|A0A067N6Z6|A0A067N6Z6_PLEOS	DNA-directed RNA polymerase OS=Pleurotus ostreatus PC15 OX=1137138 GN=PLEOSDRAFT_1047914 PE=3 SV=1	1
462	tr|A0A067N566|A0A067N566_PLEOS	Uncharacterized protein OS=Pleurotus ostreatus PC15 OX=1137138 GN=PLEOSDRAFT_1090751 PE=4 SV=1	1
463	tr|A0A067NX49|A0A067NX49_PLEOS	NDUFS2, NADH ubiquinone oxidoreductase 49 kd subunit OS=Pleurotus ostreatus PC15 OX=1137138 GN=NDUFS2 PE=3 SV=1	1
464	tr|A0A067NRK4|A0A067NRK4_PLEOS	Mitochondrial import inner membrane translocase subunit TIM10 OS=Pleurotus ostreatus PC15 OX=1137138 GN=PLEOSDRAFT_1101545 PE=3 SV=1	1
465	tr|A0A067P1R2|A0A067P1R2_PLEOS	Alpha-NAC OS=Pleurotus ostreatus PC15 OX=1137138 GN=PLEOSDRAFT_1052034 PE=3 SV=1	1
466	tr|A0A067P2I9|A0A067P2I9_PLEOS	Small nuclear ribonucleoprotein Sm D2 OS=Pleurotus ostreatus PC15 OX=1137138 GN=PLEOSDRAFT_1088461 PE=3 SV=1	2
467	tr|A0A067P619|A0A067P619_PLEOS	Uncharacterized protein OS=Pleurotus ostreatus PC15 OX=1137138 GN=PLEOSDRAFT_43137 PE=4 SV=1	1
468	tr|A0A067P5E0|A0A067P5E0_PLEOS	COX5A, subunit of cytochrome c oxidase OS=Pleurotus ostreatus PC15 OX=1137138 GN=COX5A PE=3 SV=1	2
469	tr|O94154|O94154_PLEOS	Catalase OS=Pleurotus ostreatus	1

		OX=5322 GN=pcat2 PE=3 SV=1	
469	tr|A0A067NWW1|A0A067NWW1_PLEOS	Catalase OS=Pleurotus ostreatus PC15 OX=1137138 GN=PLEOSDRAFT_1111887 PE=3 SV=1	1
469	tr|A0A482GQN6|A0A482GQN6_PLEOS	Catalase OS=Pleurotus ostreatus OX=5322 PE=2 SV=1	1
469	tr|A0A2H4UZK6|A0A2H4UZK6_PLEOS	Catalase (Fragment) OS=Pleurotus ostreatus OX=5322 GN=cat1 PE=3 SV=1	1
470	tr|A0A067NI47|A0A067NI47_PLEOS	Glycoside hydrolase family 30 protein OS=Pleurotus ostreatus PC15 OX=1137138 GN=PLEOSDRAFT_1114125 PE=3 SV=1	1
471	tr|A0A067NKU9|A0A067NKU9_PLEOS	U6 snRNA-associated Sm-like protein LSm2 OS=Pleurotus ostreatus PC15 OX=1137138 GN=PLEOSDRAFT_1104337 PE=3 SV=1	1
472	tr|A0A067NA28|A0A067NA28_PLEOS	Uncharacterized protein OS=Pleurotus ostreatus PC15 OX=1137138 GN=PLEOSDRAFT_174441 PE=4 SV=1	3
473	tr|A0A067P4Y6|A0A067P4Y6_PLEOS	Uncharacterized protein OS=Pleurotus ostreatus PC15 OX=1137138 GN=PLEOSDRAFT_1082174 PE=3 SV=1	1
474	tr|A0A067PBT5|A0A067PBT5_PLEOS	Pyridoxal 5'-phosphate synthase (glutamine hydrolyzing) OS=Pleurotus ostreatus PC15 OX=1137138 GN=PLEOSDRAFT_1052656 PE=3 SV=1	1
475	tr|A0A067NV77|A0A067NV77_PLEOS	26S proteasome regulatory subunit RPN2 OS=Pleurotus ostreatus PC15 OX=1137138 GN=PLEOSDRAFT_1111576 PE=3 SV=1	1
476	tr|A0A067NKF6|A0A067NKF6_PLEOS	Uncharacterized protein OS=Pleurotus ostreatus PC15 OX=1137138 GN=PLEOSDRAFT_1096842 PE=4 SV=1	1
477	tr|A0A067NG49|A0A067NG49_PLEOS	Uncharacterized protein OS=Pleurotus ostreatus PC15 OX=1137138 GN=PLEOSDRAFT_1113316 PE=4 SV=1	1
478	tr|A0A067NGX1|A0A067NGX1_PLEOS	Uncharacterized protein OS=Pleurotus	1

		ostreatus PC15 OX=1137138 GN=PLEOSDRAFT_158972 PE=4 SV=1	
479	tr|Q9C1M8|Q9C1M8_PLESA	Catalase OS=Pleurotus sajor-caju OX=50053 PE=2 SV=1	22
480	tr|A0A067P6P6|A0A067P6P6_PLEOS	Histone H3 OS=Pleurotus ostreatus PC15 OX=1137138 GN=PLEOSDRAFT_1070599 PE=3 SV=1	1
480	tr|A0A067NQZ3|A0A067NQZ3_PLEOS	Histone H3 OS=Pleurotus ostreatus PC15 OX=1137138 GN=PLEOSDRAFT_1088405 PE=3 SV=1	1
480	tr|A0A067NIP1|A0A067NIP1_PLEOS	Histone H3 OS=Pleurotus ostreatus PC15 OX=1137138 GN=PLEOSDRAFT_1057320 PE=3 SV=1	1
481	tr|A0A067NRM1|A0A067NRM1_PLEOS	Uncharacterized protein OS=Pleurotus ostreatus PC15 OX=1137138 GN=PLEOSDRAFT_1053760 PE=4 SV=1	1
482	tr|A0A067NG33|A0A067NG33_PLEOS	Glycogen [starch] synthase OS=Pleurotus ostreatus PC15 OX=1137138 GN=PLEOSDRAFT_1114673 PE=3 SV=1	1
483	tr|A0A067P8F7|A0A067P8F7_PLEOS	Plug_translocon domain-containing protein OS=Pleurotus ostreatus PC15 OX=1137138 GN=PLEOSDRAFT_1074503 PE=3 SV=1	1
484	tr|A0A067P107|A0A067P107_PLEOS	TIP120 domain-containing protein OS=Pleurotus ostreatus PC15 OX=1137138 GN=PLEOSDRAFT_1035867 PE=3 SV=1	1
485	tr|A0A067N4A7|A0A067N4A7_PLEOS	Uncharacterized protein (Fragment) OS=Pleurotus ostreatus PC15 OX=1137138 GN=PLEOSDRAFT_1051364 PE=4 SV=1	1
486	tr|A0A067NV66|A0A067NV66_PLEOS	UBC core domain-containing protein OS=Pleurotus ostreatus PC15 OX=1137138 GN=PLEOSDRAFT_1091903 PE=3 SV=1	1
487	tr|A0A067NJ05|A0A067NJ05_PLEOS	Cytochrome c1, component of the mitochondrial respiratory chain OS=Pleurotus ostreatus PC15	1

		OX=1137138 GN=CYT1 PE=4 SV=1	
488	tr|A0A067NXU3|A0A067NXU3_PLEOS	Isocitrate dehydrogenase [NADP] OS=Pleurotus ostreatus PC15 OX=1137138 GN=PLEOSDRAFT_48714 PE=3 SV=1	1
489	tr|A0A067PAK5|A0A067PAK5_PLEOS	Aldo_ket_red domain-containing protein OS=Pleurotus ostreatus PC15 OX=1137138 GN=PLEOSDRAFT_1087819 PE=4 SV=1	1
490	tr|A0A067NY19|A0A067NY19_PLEOS	DHHA2 domain-containing protein OS=Pleurotus ostreatus PC15 OX=1137138 GN=PLEOSDRAFT_48690 PE=4 SV=1	1
491	tr|A0A067PBM1|A0A067PBM1_PLEOS	Uncharacterized protein OS=Pleurotus ostreatus PC15 OX=1137138 GN=PLEOSDRAFT_1033460 PE=4 SV=1	1
492	tr|A0A067P131|A0A067P131_PLEOS	Phosphotransferase OS=Pleurotus ostreatus PC15 OX=1137138 GN=PLEOSDRAFT_1091253 PE=3 SV=1	1
493	tr|A0A067NDV1|A0A067NDV1_PLEOS	Phosphatidylserine decarboxylase proenzyme 2 OS=Pleurotus ostreatus PC15 OX=1137138 GN=PSD2 PE=3 SV=1	1
494	tr|A0A067ND66|A0A067ND66_PLEOS	Dihydrolipoyllysine-residue succinyltransferase OS=Pleurotus ostreatus PC15 OX=1137138 GN=PLEOSDRAFT_1078199 PE=3 SV=1	1
494	tr|A0A067NVA9|A0A067NVA9_PLEOS	Dihydrolipoyllysine-residue succinyltransferase OS=Pleurotus ostreatus PC15 OX=1137138 GN=PLEOSDRAFT_1053292 PE=3 SV=1	1
495	tr|A0A067P258|A0A067P258_PLEOS	Cytochrome b-c1 complex subunit 7 OS=Pleurotus ostreatus PC15 OX=1137138 GN=QCR7 PE=3 SV=1	1
496	tr|A0A067P470|A0A067P470_PLEOS	Uncharacterized protein OS=Pleurotus ostreatus PC15 OX=1137138 GN=PLEOSDRAFT_1088477 PE=3 SV=1	2
497	tr|A0A067NYN0|A0A067NYN0_PLEOS	Uncharacterized protein OS=Pleurotus ostreatus PC15 OX=1137138	1

		GN=PLEOSDRAFT_1091279 PE=4 SV=1	
498	tr|A0A067N5Q8|A0A067N5Q8_PLEOS	Glutamate decarboxylase OS=Pleurotus ostreatus PC15 OX=1137138 GN=PLEOSDRAFT_174888 PE=3 SV=1	1
499	tr|A0A067P591|A0A067P591_PLEOS	Histidine--tRNA ligase (Fragment) OS=Pleurotus ostreatus PC15 OX=1137138 GN=PLEOSDRAFT_1036410 PE=3 SV=1	1
500	tr|A0A067NZM0|A0A067NZM0_PLEOS	PHB domain-containing protein OS=Pleurotus ostreatus PC15 OX=1137138 GN=PLEOSDRAFT_39521 PE=3 SV=1	1
501	tr|A0A067NXP9|A0A067NXP9_PLEOS	Uncharacterized protein OS=Pleurotus ostreatus PC15 OX=1137138 GN=PLEOSDRAFT_1061874 PE=4 SV=1	1
502	tr|A0A067P091|A0A067P091_PLEOS	CCT-alpha OS=Pleurotus ostreatus PC15 OX=1137138 GN=PLEOSDRAFT_1091554 PE=3 SV=1	1
503	tr|A0A067N7V4|A0A067N7V4_PLEOS	NAD(P)-bd_dom domain-containing protein OS=Pleurotus ostreatus PC15 OX=1137138 GN=PLEOSDRAFT_1108385 PE=4 SV=1	1
504	tr|A0A067NRI8|A0A067NRI8_PLEOS	Uncharacterized protein OS=Pleurotus ostreatus PC15 OX=1137138 GN=PLEOSDRAFT_1055630 PE=4 SV=1	1
505	tr|A0A067PDG2|A0A067PDG2_PLEOS	eRF1_1 domain-containing protein OS=Pleurotus ostreatus PC15 OX=1137138 GN=PLEOSDRAFT_1061512 PE=3 SV=1	1
506	tr|A0A067P8C7|A0A067P8C7_PLEOS	Uncharacterized protein OS=Pleurotus ostreatus PC15 OX=1137138 GN=PLEOSDRAFT_1111111 PE=4 SV=1	2
507	tr|A0A067NPW7|A0A067NPW7_PLEOS	Uncharacterized protein OS=Pleurotus ostreatus PC15 OX=1137138 GN=PLEOSDRAFT_1054987 PE=4 SV=1	1
508	tr|A0A067NY79|A0A067NY79_PLEOS	PCI domain-containing protein OS=Pleurotus ostreatus PC15 OX=1137138 GN=PLEOSDRAFT_1034736	2

		PE=3 SV=1	
509	tr|A0A067NN93|A0A067NN93_PLEOS	Prohibitin OS=Pleurotus ostreatus PC15 OX=1137138 GN=PLEOSDRAFT_1044936 PE=3 SV=1	1
510	tr|A0A067NTG4|A0A067NTG4_PLEOS	Peptidase A1 domain-containing protein OS=Pleurotus ostreatus PC15 OX=1137138 GN=PLEOSDRAFT_1089322 PE=3 SV=1	1
511	tr|A0A088S7J9|A0A088S7J9_PLEOS	Peptide-methionine (S)-S-oxide reductase OS=Pleurotus ostreatus OX=5322 PE=2 SV=1	1
511	tr|A0A067N9R6|A0A067N9R6_PLEOS	Peptide-methionine (S)-S-oxide reductase OS=Pleurotus ostreatus PC15 OX=1137138 GN=PLEOSDRAFT_46396 PE=3 SV=1	1
512	tr|A0A067N771|A0A067N771_PLEOS	NMO domain-containing protein (Fragment) OS=Pleurotus ostreatus PC15 OX=1137138 GN=PLEOSDRAFT_1047903 PE=4 SV=1	1
513	tr|A0A067P030|A0A067P030_PLEOS	Uncharacterized protein OS=Pleurotus ostreatus PC15 OX=1137138 GN=PLEOSDRAFT_1033115 PE=4 SV=1	1
514	tr|A0A067NI18|A0A067NI18_PLEOS	Delta-aminolevulinic acid dehydratase OS=Pleurotus ostreatus PC15 OX=1137138 GN=PLEOSDRAFT_1093313 PE=3 SV=1	1
515	tr|A0A067NEN5|A0A067NEN5_PLEOS	Uncharacterized protein OS=Pleurotus ostreatus PC15 OX=1137138 GN=PLEOSDRAFT_1077434 PE=4 SV=1	1
516	tr|A0A067N956|A0A067N956_PLEOS	Uncharacterized protein OS=Pleurotus ostreatus PC15 OX=1137138 GN=PLEOSDRAFT_1090372 PE=3 SV=1	1
517	tr|A0A067NVQ6|A0A067NVQ6_PLEOS	S1 motif domain-containing protein OS=Pleurotus ostreatus PC15 OX=1137138 GN=PLEOSDRAFT_1088225 PE=3 SV=1	1
518	tr|A0A067NZF0|A0A067NZF0_PLEOS	Dipeptidyl peptidase 3 OS=Pleurotus ostreatus PC15 OX=1137138	2

		GN=PLEOSDRAFT_1091412 PE=3 SV=1	
519	tr|A0A067NMX5|A0A067NMX5_PLEOS	NDUFA4, NADH dehydrogenase alpha subcomplex, 9kDa subunit OS=Pleurotus ostreatus PC15 OX=1137138 GN=NDUFA4 PE=4 SV=1	1
520	tr|A0A067P322|A0A067P322_PLEOS	AAA domain-containing protein OS=Pleurotus ostreatus PC15 OX=1137138 GN=PLEOSDRAFT_1061289 PE=3 SV=1	1
521	tr|A0A067N2L3|A0A067N2L3_PLEOS	Uncharacterized protein OS=Pleurotus ostreatus PC15 OX=1137138 GN=PLEOSDRAFT_1090919 PE=4 SV=1	1
522	tr|A0A067NW73|A0A067NW73_PLEOS	Uncharacterized protein OS=Pleurotus ostreatus PC15 OX=1137138 GN=PLEOSDRAFT_1083804 PE=4 SV=1	2
523	tr|A0A067NKG1|A0A067NKG1_PLEOS	Glyco_hydro_63 domain-containing protein OS=Pleurotus ostreatus PC15 OX=1137138 GN=PLEOSDRAFT_1056050 PE=3 SV=1	1
524	tr|A0A067P5P8|A0A067P5P8_PLEOS	Protein transport protein SEC23 OS=Pleurotus ostreatus PC15 OX=1137138 GN=PLEOSDRAFT_1091812 PE=3 SV=1	1
525	tr|A0A067NXJ2|A0A067NXJ2_PLEOS	Uncharacterized protein OS=Pleurotus ostreatus PC15 OX=1137138 GN=PLEOSDRAFT_1063593 PE=4 SV=1	1
526	tr|A0A067NY18|A0A067NY18_PLEOS	Uncharacterized protein OS=Pleurotus ostreatus PC15 OX=1137138 GN=PLEOSDRAFT_154062 PE=4 SV=1	1
527	tr|A0A067PDE2|A0A067PDE2_PLEOS	Glycoside hydrolase family 55 protein OS=Pleurotus ostreatus PC15 OX=1137138 GN=PLEOSDRAFT_37178 PE=4 SV=1	2
528	tr|A0A067NVE7|A0A067NVE7_PLEOS	Methionine adenosyltransferase 2 subunit beta OS=Pleurotus ostreatus PC15 OX=1137138 GN=PLEOSDRAFT_1088122 PE=3 SV=1	2

529	tr|A0A067N4B4|A0A067N4B4_PLEOS	Uncharacterized protein OS=Pleurotus ostreatus PC15 OX=1137138 GN=PLEOSDRAFT_1060105 PE=4 SV=1	1
530	tr|A0A067NG60|A0A067NG60_PLEOS	Uncharacterized protein OS=Pleurotus ostreatus PC15 OX=1137138 GN=PLEOSDRAFT_1089953 PE=4 SV=1	1
531	tr|A0A067P386|A0A067P386_PLEOS	Acetylglucosamine phosphomutase OS=Pleurotus ostreatus PC15 OX=1137138 GN=PLEOSDRAFT_1092332 PE=3 SV=1	1
532	tr|A0A067P375|A0A067P375_PLEOS	Uncharacterized protein OS=Pleurotus ostreatus PC15 OX=1137138 GN=PLEOSDRAFT_1062802 PE=4 SV=1	1
533	tr|A0A067NWY0|A0A067NWY0_PLEOS	Uncharacterized protein OS=Pleurotus ostreatus PC15 OX=1137138 GN=PLEOSDRAFT_1112875 PE=3 SV=1	2
534	tr|A0A067NL68|A0A067NL68_PLEOS	ATP synthase subunit delta, mitochondrial OS=Pleurotus ostreatus PC15 OX=1137138 GN=ATP16 PE=3 SV=1	1
535	RRRRRtr|A0A067P419|A0A067P419_PLEOS	REVERSED Uncharacterized protein OS=Pleurotus ostreatus PC15 OX=1137138 GN=PLEOSDRAFT_1035414 PE=4 SV=1	1
536	tr|A0A067NT24|A0A067NT24_PLEOS	Uncharacterized protein OS=Pleurotus ostreatus PC15 OX=1137138 GN=PLEOSDRAFT_1095851 PE=4 SV=1	1
537	tr|A0A067P266|A0A067P266_PLEOS	Uncharacterized protein OS=Pleurotus ostreatus PC15 OX=1137138 GN=PLEOSDRAFT_1100995 PE=4 SV=1	1
538	tr|A0A067NNJ9|A0A067NNJ9_PLEOS	1,4-alpha-glucan-branching enzyme (Fragment) OS=Pleurotus ostreatus PC15 OX=1137138 GN=PLEOSDRAFT_1112299 PE=3 SV=1	1
539	tr|A0A067NQS1|A0A067NQS1_PLEOS	Uncharacterized protein OS=Pleurotus ostreatus PC15 OX=1137138 GN=PLEOSDRAFT_1097206 PE=4 SV=1	1
539	tr|A0A067NHL1|A0A067NHL1_PLEOS	Uncharacterized protein OS=Pleurotus ostreatus PC15 OX=1137138	1

		GN=PLEOSDRAFT_1113075 PE=4 SV=1	
540	tr|A0A067NG67|A0A067NG67_PLEOS	Uncharacterized protein OS=Pleurotus ostreatus PC15 OX=1137138 GN=PLEOSDRAFT_1097379 PE=4 SV=1	1
541	tr|A0A067N3C6|A0A067N3C6_PLEOS	Uncharacterized protein OS=Pleurotus ostreatus PC15 OX=1137138 GN=PLEOSDRAFT_1098291 PE=4 SV=1	1
542	tr|A0A067NF06|A0A067NF06_PLEOS	AMP_N domain-containing protein OS=Pleurotus ostreatus PC15 OX=1137138 GN=PLEOSDRAFT_36612 PE=3 SV=1	1
543	tr|A0A067NP99|A0A067NP99_PLEOS	Ubiquitin-like domain-containing protein OS=Pleurotus ostreatus PC15 OX=1137138 GN=PLEOSDRAFT_1089245 PE=4 SV=1	1
544	tr|A0A067NRY6|A0A067NRY6_PLEOS	Phosphatidylglycerol/phosphatidylinositol transfer protein OS=Pleurotus ostreatus PC15 OX=1137138 GN=PLEOSDRAFT_1088496 PE=3 SV=1	1
545	tr|A0A067NSW7|A0A067NSW7_PLEOS	Proteasome subunit beta OS=Pleurotus ostreatus PC15 OX=1137138 GN=PLEOSDRAFT_1036095 PE=3 SV=1	1
546	tr|A0A067NDI3|A0A067NDI3_PLEOS	RRM domain-containing protein OS=Pleurotus ostreatus PC15 OX=1137138 GN=PLEOSDRAFT_1098260 PE=4 SV=1	1
548	tr|A0A067ND49|A0A067ND49_PLEOS	Thioredoxin domain-containing protein OS=Pleurotus ostreatus PC15 OX=1137138 GN=PLEOSDRAFT_1094465 PE=3 SV=1	1
549	tr|A0A067P360|A0A067P360_PLEOS	Chorismate synthase OS=Pleurotus ostreatus PC15 OX=1137138 GN=PLEOSDRAFT_1036017 PE=3 SV=1	1
550	tr|A0A067NMU1|A0A067NMU1_PLEOS	Pyruvate dehydrogenase E1 component subunit beta OS=Pleurotus ostreatus PC15 OX=1137138 GN=PLEOSDRAFT_1039228 PE=4 SV=1	1

551	tr|A0A067NVI9|A0A067NVI9_PLEOS	ADF-H domain-containing protein OS=Pleurotus ostreatus PC15 OX=1137138 GN=PLEOSDRAFT_1061975 PE=4 SV=1	2
552	tr|A0A067P420|A0A067P420_PLEOS	V-type proton ATPase subunit a OS=Pleurotus ostreatus PC15 OX=1137138 GN=PLEOSDRAFT_1091713 PE=3 SV=1	1
553	tr|A0A067ND43|A0A067ND43_PLEOS	Carbonic anhydrase OS=Pleurotus ostreatus PC15 OX=1137138 GN=PLEOSDRAFT_1058116 PE=3 SV=1	1
554	tr|A0A067NAA3|A0A067NAA3_PLEOS	SPX domain-containing protein OS=Pleurotus ostreatus PC15 OX=1137138 GN=PLEOSDRAFT_52207 PE=4 SV=1	1
555	tr|A0A067PDC4|A0A067PDC4_PLEOS	PKS_ER domain-containing protein OS=Pleurotus ostreatus PC15 OX=1137138 GN=PLEOSDRAFT_1033378 PE=4 SV=1	1
556	tr|A0A067P2K4|A0A067P2K4_PLEOS	KOW domain-containing protein OS=Pleurotus ostreatus PC15 OX=1137138 GN=PLEOSDRAFT_1091506 PE=3 SV=1	1
557	tr|A0A067P6B2|A0A067P6B2_PLEOS	Uncharacterized protein OS=Pleurotus ostreatus PC15 OX=1137138 GN=PLEOSDRAFT_1074367 PE=3 SV=1	1
558	tr|A0A067NH04|A0A067NH04_PLEOS	Profilin OS=Pleurotus ostreatus PC15 OX=1137138 GN=PLEOSDRAFT_1098183 PE=3 SV=1	1
559	RRRRRtr|A0A067NJD8|A0A067NJD8_PLEOS	REVERSED Uncharacterized protein OS=Pleurotus ostreatus PC15 OX=1137138 GN=PLEOSDRAFT_52581 PE=4 SV=1	1
560	tr|A0A067NAS1|A0A067NAS1_PLEOS	Uncharacterized protein OS=Pleurotus ostreatus PC15 OX=1137138 GN=PLEOSDRAFT_1085946 PE=4 SV=1	1
561	tr|A0A067NH25|A0A067NH25_PLEOS	UBC core domain-containing protein OS=Pleurotus ostreatus PC15	1

		OX=1137138 GN=PLEOSDRAFT_1068145 PE=3 SV=1	
562	tr|A0A067NZG7|A0A067NZG7_PLEOS	Glucosidase 2 subunit beta OS=Pleurotus ostreatus PC15 OX=1137138 GN=PLEOSDRAFT_1055546 PE=4 SV=1	1
563	tr|A0A067NSE0|A0A067NSE0_PLEOS	PCI domain-containing protein OS=Pleurotus ostreatus PC15 OX=1137138 GN=PLEOSDRAFT_1054135 PE=3 SV=1	1
564	tr|A0A067NCR9|A0A067NCR9_PLEOS	Eukaryotic translation initiation factor 6 OS=Pleurotus ostreatus PC15 OX=1137138 GN=TIF6 PE=3 SV=1	1
566	tr|A0A067NQ58|A0A067NQ58_PLEOS	Proteasome subunit beta OS=Pleurotus ostreatus PC15 OX=1137138 GN=PLEOSDRAFT_1063908 PE=3 SV=1	1
567	tr|A0A067N3M1|A0A067N3M1_PLEOS	Uncharacterized protein OS=Pleurotus ostreatus PC15 OX=1137138 GN=PLEOSDRAFT_1114640 PE=4 SV=1	1
568	tr|A0A067NYW8|A0A067NYW8_PLEOS	Uncharacterized protein OS=Pleurotus ostreatus PC15 OX=1137138 GN=PLEOSDRAFT_1110413 PE=4 SV=1	1
569	tr|A0A067N4U3|A0A067N4U3_PLEOS	Uncharacterized protein OS=Pleurotus ostreatus PC15 OX=1137138 GN=PLEOSDRAFT_1073003 PE=4 SV=1	1
572	tr|A0A067NYN3|A0A067NYN3_PLEOS	6,7-dimethyl-8-ribityllumazine synthase OS=Pleurotus ostreatus PC15 OX=1137138 GN=PLEOSDRAFT_1060713 PE=3 SV=1	1
573	tr|A0A067NFB0|A0A067NFB0_PLEOS	Dynein light chain OS=Pleurotus ostreatus PC15 OX=1137138 GN=PLEOSDRAFT_1057224 PE=3 SV=1	1
574	tr|A0A067NXJ7|A0A067NXJ7_PLEOS	Uncharacterized protein OS=Pleurotus ostreatus PC15 OX=1137138 GN=PLEOSDRAFT_1075744 PE=4 SV=1	1
575	tr|A0A067ND80|A0A067ND80_PLEOS	PSDC domain-containing protein OS=Pleurotus ostreatus PC15 OX=1137138 GN=PLEOSDRAFT_1094176	1

		PE=4 SV=1	
579	tr|A0A067P596|A0A067P596_PLEOS	RRM domain-containing protein OS=Pleurotus ostreatus PC15 OX=1137138 GN=PLEOSDRAFT_173268 PE=4 SV=1	1
581	tr|A0A067NUZ3|A0A067NUZ3_PLEOS	Phosphoenolpyruvate carboxykinase (ATP) (Fragment) OS=Pleurotus ostreatus PC15 OX=1137138 GN=PLEOSDRAFT_1089538 PE=4 SV=1	1
585	tr|A0A067NN90|A0A067NN90_PLEOS	40S ribosomal protein S25 OS=Pleurotus ostreatus PC15 OX=1137138 GN=PLEOSDRAFT_1064353 PE=3 SV=1	2
595	tr|A0A067N6E3|A0A067N6E3_PLEOS	SGNH_hydro domain-containing protein OS=Pleurotus ostreatus PC15 OX=1137138 GN=PLEOSDRAFT_34651 PE=4 SV=1	1
623	RRRRRtr|A0A067NKG9|A0A067NKG9_PLEOS	REVERSED IPPc domain-containing protein OS=Pleurotus ostreatus PC15 OX=1137138 GN=PLEOSDRAFT_52409 PE=4 SV=1	1
637	RRRRRtr|A0A067NJC7|A0A067NJC7_PLEOS	REVERSED Uncharacterized protein OS=Pleurotus ostreatus PC15 OX=1137138 GN=PLEOSDRAFT_1089531 PE=4 SV=1	1
639	tr|A0A067N8U4|A0A067N8U4_PLEOS	6-phosphogluconate dehydrogenase, decarboxylating OS=Pleurotus ostreatus PC15 OX=1137138 GN=PLEOSDRAFT_41725 PE=3 SV=1	28
650	RRRRRtr|A0A067NBS9|A0A067NBS9_PLEOS	REVERSED Glutathione synthetase OS=Pleurotus ostreatus PC15 OX=1137138 GN=PLEOSDRAFT_1046210 PE=3 SV=1	1
658	tr|A0A2Z4PGK3|A0A2Z4PGK3_PLEOS	Alpha,alpha-trehalose-phosphate synthase (UDP-forming) OS=Pleurotus ostreatus OX=5322 PE=2 SV=1	1
658	tr|A0A2S1Q3T7|A0A2S1Q3T7_9AGAR	Alpha,alpha-trehalose-phosphate synthase (UDP-forming) OS=Pleurotus tuoliensis OX=879823 GN=tps PE=2 SV=1	1

658	tr|A0A067NCF7|A0A067NCF7_PLEOS	Alpha,alpha-trehalose-phosphate synthase (UDP-forming) OS=Pleurotus ostreatus PC15 OX=1137138 GN=PLEOSDRAFT_1090402 PE=4 SV=1	1
661	tr|Q8X1M9|Q8X1M9_PLEOS	Pleurotolysin A OS=Pleurotus ostreatus OX=5322 GN=plyA PE=1 SV=1	29
661	tr|T2HUL2|T2HUL2_PLEER	Pe.pleurotolysin A OS=Pleurotus eryngii OX=5323 GN=Pe.PlyA PE=2 SV=1	26

Peptides: 95% confidence of identification, OS: organism name, OX: organism identifier GN: Gene name, PE: Protein existence and SV: Sequence version.

Table S2: Protein identification of *Lentinula edodes by Uniprot database*

N	Accession	Protein Name/Species	Peptides
1	tr|A0A1Q3EIF8|A0A1Q3EIF8_LENED	Actin 1 OS=Lentinula edodes OX=5353 GN=LENED_008925 PE=3 SV=1	23
2	tr|A0A1Q3E953|A0A1Q3E953_LENED	Elongation factor 1-alpha OS=Lentinula edodes OX=5353 GN=LENED_005448 PE=3 SV=1	27
3	tr|A0A1Q3EMJ0|A0A1Q3EMJ0_LENED	Heat shock protein HSS1 OS=Lentinula edodes OX=5353 GN=LENED_010478 PE=3 SV=1	20
4	tr|A0A1Q3ENT5|A0A1Q3ENT5_LENED	ATP synthase subunit beta OS=Lentinula edodes OX=5353 GN=LENED_010958 PE=3 SV=1	20
5	tr|A0A1Q3E9P0|A0A1Q3E9P0_LENED	Tubulin beta chain OS=Lentinula edodes OX=5353 GN=LENED_005691 PE=3 SV=1	13
6	tr|A0A1Q3E0W6|A0A1Q3E0W6_LENED	14-3-3 protein OS=Lentinula edodes OX=5353 GN=LENED_002351 PE=3 SV=1	19
6	tr|Q9UR29|Q9UR29_LENED	14-3-3 OS=Lentinula edodes OX=5353 PE=2 SV=1	18

7	tr|A0A1Q3DV62|A0A1Q3DV62_LENED	Elongation factor 2 OS=Lentinula edodes OX=5353 GN=LENED_000301 PE=3 SV=1	12
8	tr|A0A1Q3EJ33|A0A1Q3EJ33_LENED	Valosin-containing protein OS=Lentinula edodes OX=5353 GN=LENED_009161 PE=4 SV=1	7
9	tr|A0A1Q3EFH4|A0A1Q3EFH4_LENED	ATP synthase F1 alpha subunit OS=Lentinula edodes OX=5353 GN=LENED_007868 PE=3 SV=1	8
10	tr|A0A1Q3DVR0|A0A1Q3DVR0_LENED	Alpha beta-hydrolase OS=Lentinula edodes OX=5353 GN=LENED_000429 PE=3 SV=1	9
11	tr|A0A1Q3EFN7|A0A1Q3EFN7_LENED	5-methyltetrahydropteroyltriglutamate--homocysteine S-methyltransferase OS=Lentinula edodes OX=5353 GN=LENED_007892 PE=3 SV=1	8
12	tr|A0A1Q3DY35|A0A1Q3DY35_LENED	D-fructose-6-phosphate amidotransferase OS=Lentinula edodes OX=5353 GN=LENED_001319 PE=4 SV=1	5
13	tr|A0A1Q3E4Z7|A0A1Q3E4Z7_LENED	Heat-shock protein 90 OS=Lentinula edodes OX=5353 GN=LENED_003972 PE=3 SV=1	7
14	tr|A0A1Q3E0L1|A0A1Q3E0L1_LENED	Formate dehydrogenase OS=Lentinula edodes OX=5353 GN=LENED_002235 PE=3 SV=1	12
15	tr|A0A1Q3EMN1|A0A1Q3EMN1_LENED	Heat shock HSP70 protein OS=Lentinula edodes OX=5353 GN=LENED_010527 PE=3 SV=1	9
16	tr|A0A1Q3E8X6|A0A1Q3E8X6_LENED	40s ribosomal protein s3 OS=Lentinula edodes OX=5353 GN=LENED_005435 PE=3 SV=1	4
17	tr|A0A1Q3EA12|A0A1Q3EA12_LENED	Adenosylhomocysteinase OS=Lentinula edodes OX=5353 GN=LENED_005751 PE=3 SV=1	9
18	tr|A0A1Q3EQ96|A0A1Q3EQ96_LENED	Ubiquitin OS=Lentinula edodes OX=5353 GN=LENED_011525 PE=4	8

		SV=1	
18	tr|A0A1Q3EE24|A0A1Q3EE24_LENED	Polyubiquitin Ubi4 OS=Lentinula edodes OX=5353 GN=LENED_007305 PE=4 SV=1	8
18	tr|A0A1Q3EDZ6|A0A1Q3EDZ6_LENED	Ubiquitin C OS=Lentinula edodes OX=5353 GN=LENED_007306 PE=4 SV=1	8
18	tr|A0A1Q3E5S6|A0A1Q3E5S6_LENED	Ubiquitin OS=Lentinula edodes OX=5353 GN=LENED_004256 PE=3 SV=1	8
18	tr|A0A1Q3DZN1|A0A1Q3DZN1_LENED	Ubiquitin-domain-containing protein OS=Lentinula edodes OX=5353 GN=LENED_002001 PE=3 SV=1	8
19	tr|A0A1Q3E7V1|A0A1Q3E7V1_LENED	Histone H4 OS=Lentinula edodes OX=5353 GN=LENED_004960 PE=3 SV=1	6
19	tr|A0A1Q3EJ42|A0A1Q3EJ42_LENED	Histone H4 OS=Lentinula edodes OX=5353 GN=LENED_009215 PE=3 SV=1	5
19	tr|A0A1Q3EHM2|A0A1Q3EHM2_LENED	Histone H4 OS=Lentinula edodes OX=5353 GN=LENED_008656 PE=3 SV=1	5
19	tr|A0A1Q3E7P0|A0A1Q3E7P0_LENED	Histone H4 OS=Lentinula edodes OX=5353 GN=LENED_004964 PE=3 SV=1	5
19	tr|A0A1Q3E4V0|A0A1Q3E4V0_LENED	Histone H4 OS=Lentinula edodes OX=5353 GN=LENED_003908 PE=3 SV=1	5
19	tr|A0A1Q3DZB6|A0A1Q3DZB6_LENED	Histone H4 OS=Lentinula edodes OX=5353 GN=LENED_001866 PE=3 SV=1	5
20	tr|A0A1Q3ERH4|A0A1Q3ERH4_LENED	Heat shock protein 60 OS=Lentinula edodes OX=5353 GN=LENED_011894 PE=3 SV=1	4
21	tr|A0A1Q3EQW0|A0A1Q3EQW0_LENED	40S ribosomal protein S9 OS=Lentinula edodes OX=5353 GN=LENED_011753 PE=3 SV=1	4

22	tr|A0A1Q3E4Y6|A0A1Q3E4Y6_LENED	Small gtpase-binding protein OS=Lentinula edodes OX=5353 GN=LENED_003731 PE=4 SV=1	5
23	tr|A0A1Q3DXT9|A0A1Q3DXT9_LENED	Serine hydroxymethyltransferase OS=Lentinula edodes OX=5353 GN=LENED_001126 PE=3 SV=1	5
24	tr|A0A1Q3EFN5|A0A1Q3EFN5_LENED	Rab GDP dissociation inhibitor OS=Lentinula edodes OX=5353 GN=LENED_007921 PE=3 SV=1	4
25	tr|A0A1Q3E947|A0A1Q3E947_LENED	Phosphopyruvate hydratase OS=Lentinula edodes OX=5353 GN=LENED_005486 PE=3 SV=1	8
26	tr|A0A1Q3E1A7|A0A1Q3E1A7_LENED	Catalase OS=Lentinula edodes OX=5353 GN=LENED_002518 PE=3 SV=1	6
26	tr|A0A1Q3E135|A0A1Q3E135_LENED	Catalase OS=Lentinula edodes OX=5353 GN=LENED_002517 PE=3 SV=1	6
27	tr|A0A1Q3EEZ5|A0A1Q3EEZ5_LENED	Vacuolar proton pump subunit B OS=Lentinula edodes OX=5353 GN=LENED_007618 PE=3 SV=1	4
28	tr|A0A1Q3ES48|A0A1Q3ES48_LENED	Pyruvate carboxylase OS=Lentinula edodes OX=5353 GN=LENED_012225 PE=4 SV=1	6
29	tr|A0A1Q3EFX1|A0A1Q3EFX1_LENED	Pyruvate kinase OS=Lentinula edodes OX=5353 GN=LENED_007957 PE=3 SV=1	6
29	tr|A0A1Q3EFV3|A0A1Q3EFV3_LENED	Pyruvate kinase OS=Lentinula edodes OX=5353 GN=LENED_007958 PE=3 SV=1	6
30	tr|A0A1Q3EI78|A0A1Q3EI78_LENED	Calmodulin OS=Lentinula edodes OX=5353 GN=LENED_008884 PE=3 SV=1	7
31	tr|A0A1Q3DUG1|A0A1Q3DUG1_LENED	Transaldolase OS=Lentinula edodes OX=5353 GN=LENED_000019 PE=3 SV=1	3
32	tr|A0A1Q3ESZ7|A0A1Q3ESZ7_LENED	Histone H2B OS=Lentinula edodes	5

		OX=5353 GN=LENED_012555 PE=3 SV=1	
32	tr|A0A1Q3ESW3|A0A1Q3ESW3_LENED	Histone H2B OS=Lentinula edodes OX=5353 GN=LENED_012552 PE=3 SV=1	5
32	tr|A0A1Q3ED83|A0A1Q3ED83_LENED	Histone H2B OS=Lentinula edodes OX=5353 GN=LENED_007014 PE=3 SV=1	5
33	tr|A0A1Q3E591|A0A1Q3E591_LENED	Glycosyltransferase family 4 protein OS=Lentinula edodes OX=5353 GN=LENED_003981 PE=4 SV=1	4
34	tr|A0A1Q3E2E7|A0A1Q3E2E7_LENED	E3 ubiquitin ligase complex SCF subunit OS=Lentinula edodes OX=5353 GN=LENED_002931 PE=3 SV=1	3
35	tr|A0A1Q3EHD0|A0A1Q3EHD0_LENED	Arginosuccinase OS=Lentinula edodes OX=5353 GN=LENED_008560 PE=3 SV=1	4
36	tr|A0A1Q3E3Q9|A0A1Q3E3Q9_LENED	40S ribosomal protein S13 OS=Lentinula edodes OX=5353 GN=LENED_003344 PE=3 SV=1	3
37	tr|A0A1Q3E9V9|A0A1Q3E9V9_LENED	ATP citrate synthase OS=Lentinula edodes OX=5353 GN=LENED_005754 PE=4 SV=1	3
38	tr|A0A1Q3ETI2|A0A1Q3ETI2_LENED	DEAD-domain-containing protein OS=Lentinula edodes OX=5353 GN=LENED_012798 PE=4 SV=1	5
39	tr|A0A1Q3E3B6|A0A1Q3E3B6_LENED	Malate dehydrogenase OS=Lentinula edodes OX=5353 GN=LENED_003196 PE=3 SV=1	8
40	tr|A0A1Q3ES03|A0A1Q3ES03_LENED	Ribosomal protein L15 OS=Lentinula edodes OX=5353 GN=LENED_012198 PE=3 SV=1	3
41	tr|A0A1Q3DV01|A0A1Q3DV01_LENED	Inorganic diphosphatase OS=Lentinula edodes OX=5353 GN=LENED_000233 PE=3 SV=1	4
42	tr|A0A1Q3EDI6|A0A1Q3EDI6_LENED	Multifunctional fusion protein	3

		OS=Lentinula edodes OX=5353 GN=LENED_007122 PE=3 SV=1	
43	tr|Q870G6|Q870G6_LENED	Guanine nucleotide binding protein beta subunit OS=Lentinula edodes OX=5353 GN=Gb1 PE=2 SV=2	3
44	tr|A0A6G9ENH5|A0A6G9ENH5_LENED	Ribosomal protein L4 (Fragment) OS=Lentinula edodes OX=5353 GN=rpl4 PE=2 SV=1	6
44	tr|A0A1Q3E0D6|A0A1Q3E0D6_LENED	60s ribosomal protein l2 OS=Lentinula edodes OX=5353 GN=LENED_002258 PE=3 SV=1	6
45	tr|A0A1Q3DXN7|A0A1Q3DXN7_LENED	NADP+-dependent D-mannitol dehydrogenase OS=Lentinula edodes OX=5353 GN=LENED_001165 PE=3 SV=1	3
46	tr|A0A1Q3DXZ4|A0A1Q3DXZ4_LENED	Glutathione S-transferase OS=Lentinula edodes OX=5353 GN=LENED_001278 PE=4 SV=1	3
47	tr|A0A1Q3EAN2|A0A1Q3EAN2_LENED	2-methylcitrate dehydratase OS=Lentinula edodes OX=5353 GN=LENED_006068 PE=3 SV=1	3
48	tr|A0A1Q3E465|A0A1Q3E465_LENED	Phosphoglycerate kinase OS=Lentinula edodes OX=5353 GN=LENED_003585 PE=3 SV=1	3
49	tr|A0A1Q3E920|A0A1Q3E920_LENED	Mitochondrial carrier OS=Lentinula edodes OX=5353 GN=LENED_005477 PE=3 SV=1	3
50	tr|A0A1Q3DWS6|A0A1Q3DWS6_LENED	20S proteasome subunit OS=Lentinula edodes OX=5353 GN=LENED_000919 PE=3 SV=1	3
51	tr|A0A1Q3EI91|A0A1Q3EI91_LENED	Aconitate hydratase, mitochondrial OS=Lentinula edodes OX=5353 GN=LENED_008893 PE=3 SV=1	3
52	tr|A0A1Q3EFV4|A0A1Q3EFV4_LENED	Importin subunit alpha OS=Lentinula edodes OX=5353 GN=LENED_007964 PE=3 SV=1	3
53	tr|A0A1Q3ENS8|A0A1Q3ENS8_LENED	Carbamoyl-phosphate synthase	2

		OS=Lentinula edodes OX=5353 GN=LENED_010929 PE=4 SV=1	
54	tr|A0A1Q3E242|A0A1Q3E242_LENED	60S ribosomal protein L7 OS=Lentinula edodes OX=5353 GN=LENED_002880 PE=3 SV=1	4
55	tr|A0A1Q3EEZ4|A0A1Q3EEZ4_LENED	40S ribosomal protein S12 OS=Lentinula edodes OX=5353 GN=LENED_007650 PE=3 SV=1	4
56	tr|A0A1Q3EAB8|A0A1Q3EAB8_LENED	Aldehyde dehydrogenase OS=Lentinula edodes OX=5353 GN=LENED_005944 PE=3 SV=1	3
56	tr|A0A1Q3EAK3|A0A1Q3EAK3_LENED	Aldehyde dehydrogenase OS=Lentinula edodes OX=5353 GN=LENED_005943 PE=3 SV=1	2
57	tr|A0A1Q3EF26|A0A1Q3EF26_LENED	Proteasome subunit alpha type OS=Lentinula edodes OX=5353 GN=LENED_007690 PE=3 SV=1	2
58	tr|A0A1Q3ELR1|A0A1Q3ELR1_LENED	40S ribosomal protein S0 OS=Lentinula edodes OX=5353 GN=RPS0 PE=3 SV=1	4
59	tr|A0A1Q3EQX8|A0A1Q3EQX8_LENED	Heat shock protein 70 OS=Lentinula edodes OX=5353 GN=LENED_011775 PE=4 SV=1	3
60	tr|A0A1Q3EDY8|A0A1Q3EDY8_LENED	Glucose-6-phosphate 1-dehydrogenase OS=Lentinula edodes OX=5353 GN=LENED_007192 PE=3 SV=1	3
61	tr|A0A1Q3E3H1|A0A1Q3E3H1_LENED	CCT-beta OS=Lentinula edodes OX=5353 GN=LENED_003321 PE=3 SV=1	2
62	tr|A0A1Q3EEB4|A0A1Q3EEB4_LENED	Fumarate reductase OS=Lentinula edodes OX=5353 GN=LENED_007413 PE=4 SV=1	2
63	tr|A0A1Q3E273|A0A1Q3E273_LENED	GTP-binding nuclear protein OS=Lentinula edodes OX=5353 GN=LENED_002785 PE=3 SV=1	1
64	tr|A0A1Q3E158|A0A1Q3E158_LENED	60S ribosomal protein L24	3

		OS=Lentinula edodes OX=5353 GN=LENED_002546 PE=3 SV=1	
65	tr|A0A1Q3EMA1|A0A1Q3EMA1_LENED	DEAD-domain-containing protein OS=Lentinula edodes OX=5353 GN=LENED_010403 PE=4 SV=1	3
66	tr|A0A1Q3EAC0|A0A1Q3EAC0_LENED	CCT-theta OS=Lentinula edodes OX=5353 GN=LENED_005869 PE=3 SV=1	3
67	tr|A0A1Q3E2R5|A0A1Q3E2R5_LENED	Citrulline--aspartate ligase OS=Lentinula edodes OX=5353 GN=LENED_003123 PE=3 SV=1	5
68	tr|A0A1Q3ET88|A0A1Q3ET88_LENED	Mitochondrial intermediate peptidase OS=Lentinula edodes OX=5353 GN=LENED_012676 PE=3 SV=1	2
69	tr|A0A1Q3ELK6|A0A1Q3ELK6_LENED	60S ribosomal protein L13a OS=Lentinula edodes OX=5353 GN=LENED_010028 PE=3 SV=1	2
69	tr|A0A1Q3ELC2|A0A1Q3ELC2_LENED	60S ribosomal protein L13a OS=Lentinula edodes OX=5353 GN=LENED_010027 PE=3 SV=1	2
70	tr|A0A1Q3EFW3|A0A1Q3EFW3_LENED	Cell division control GTP binding protein OS=Lentinula edodes OX=5353 GN=LENED_007969 PE=3 SV=1	3
71	tr|A0A1Q3DZ08|A0A1Q3DZ08_LENED	40S ribosomal protein S26 OS=Lentinula edodes OX=5353 GN=LENED_001749 PE=3 SV=1	3
72	tr|A0A1Q3DW87|A0A1Q3DW87_LENED	ARF SAR OS=Lentinula edodes OX=5353 GN=LENED_000433 PE=3 SV=1	2
73	tr|A0A1Q3ETC5|A0A1Q3ETC5_LENED	General substrate transporter OS=Lentinula edodes OX=5353 GN=LENED_012753 PE=4 SV=1	5
73	tr|A0A1Q3EMD9|A0A1Q3EMD9_LENED	PKS_ER domain-containing protein OS=Lentinula edodes OX=5353 GN=LENED_010425 PE=4 SV=1	2

73	tr|A0A1Q3EHM1|A0A1Q3EHM1_LENED	PKS_ER domain-containing protein OS=Lentinula edodes OX=5353 GN=LENED_008625 PE=4 SV=1	2
74	tr|A0A1Q3END7|A0A1Q3END7_LENED	Histone H2A OS=Lentinula edodes OX=5353 GN=LENED_010804 PE=3 SV=1	3
74	tr|A0A1Q3EDG0|A0A1Q3EDG0_LENED	Histone H2A OS=Lentinula edodes OX=5353 GN=LENED_007015 PE=3 SV=1	3
74	tr|A0A1Q3DZC5|A0A1Q3DZC5_LENED	Histone H2A OS=Lentinula edodes OX=5353 GN=LENED_001877 PE=3 SV=1	3
75	tr|A0A1Q3DWU1|A0A1Q3DWU1_LENED	40S ribosomal protein S7 OS=Lentinula edodes OX=5353 GN=LENED_000946 PE=3 SV=1	2
76	tr|A0A1Q3EMB3|A0A1Q3EMB3_LENED	60s ribosomal protein l18 OS=Lentinula edodes OX=5353 GN=LENED_010306 PE=3 SV=1	2
77	tr|A0A1Q3EKG4|A0A1Q3EKG4_LENED	20s proteasome subunit OS=Lentinula edodes OX=5353 GN=LENED_009667 PE=3 SV=1	2
78	tr|A0A1Q3EHU8|A0A1Q3EHU8_LENED	26S proteasome subunit P45 OS=Lentinula edodes OX=5353 GN=LENED_008747 PE=3 SV=1	2
79	tr|A0A1Q3EFS2|A0A1Q3EFS2_LENED	Methylmalonate-semialdehyde dehydrogenase (CoA acylating) OS=Lentinula edodes OX=5353 GN=LENED_007919 PE=4 SV=1	3
80	sp|Q764D2|RS3A_LENED	40S ribosomal protein S1 OS=Lentinula edodes OX=5353 GN=RPS1 PE=2 SV=1	3
81	tr|A0A1Q3E3B0|A0A1Q3E3B0_LENED	Eukaryotic translation initiation factor 5A OS=Lentinula edodes OX=5353 GN=LENED_003333 PE=3 SV=1	4
82	tr|A0A1Q3DZC7|A0A1Q3DZC7_LENED	Ras-domain-containing protein OS=Lentinula edodes OX=5353 GN=LENED_001763 PE=4 SV=1	1

83	tr|A0A1Q3E4E3|A0A1Q3E4E3_LENED	40S ribosomal protein S4 OS=Lentinula edodes OX=5353 GN=LENED_003654 PE=3 SV=1	2
84	tr|A0A1Q3EGF5|A0A1Q3EGF5_LENED	Gtp-binding protein ypt1 OS=Lentinula edodes OX=5353 GN=LENED_008182 PE=4 SV=1	1
84	tr|A0A1Q3DX36|A0A1Q3DX36_LENED	Ras-like GTP-binding protein RYL2 OS=Lentinula edodes OX=5353 GN=LENED_000945 PE=4 SV=1	1
85	tr|A0A1Q3E3J5|A0A1Q3E3J5_LENED	Glyceraldehyde-3-phosphate dehydrogenase OS=Lentinula edodes OX=5353 GN=LENED_003203 PE=3 SV=1	2
85	sp|Q9UR38|G3P_LENED	Glyceraldehyde-3-phosphate dehydrogenase OS=Lentinula edodes OX=5353 GN=gpd PE=2 SV=1	2
86	tr|A0A1Q3DV87|A0A1Q3DV87_LENED	60s ribosomal protein l6 OS=Lentinula edodes OX=5353 GN=LENED_000333 PE=3 SV=1	3
87	tr|A0A1Q3EK94|A0A1Q3EK94_LENED	Cytoplasmic protein OS=Lentinula edodes OX=5353 GN=LENED_009593 PE=4 SV=1	2
88	tr|A0A1Q3DZH6|A0A1Q3DZH6_LENED	Mitochondrial carrier OS=Lentinula edodes OX=5353 GN=LENED_001930 PE=3 SV=1	3
89	tr|A0A1Q3EM93|A0A1Q3EM93_LENED	60S ribosomal protein L11 OS=Lentinula edodes OX=5353 GN=LENED_010367 PE=3 SV=1	1
90	tr|A0A1Q3EFI5|A0A1Q3EFI5_LENED	Alpha subunit of the F1 sector of mitochondrial F1F0 ATP OS=Lentinula edodes OX=5353 GN=LENED_007869 PE=3 SV=1	4
91	tr|A0A1Q3DZM1|A0A1Q3DZM1_LENED	Phosphoenolpyruvate carboxykinase (ATP) OS=Lentinula edodes OX=5353 GN=LENED_001864 PE=3 SV=1	4
92	tr|A0A1Q3EAH5|A0A1Q3EAH5_LENED	UTP--glucose-1-phosphate uridylyltransferase OS=Lentinula edodes OX=5353 GN=LENED_006002	1

		PE=3 SV=1	
93	tr|A0A1Q3E3Z8|A0A1Q3E3Z8_LENED	Homocitrate synthase OS=Lentinula edodes OX=5353 GN=LENED_003602 PE=3 SV=1	1
94	tr|A0A1Q3EN67|A0A1Q3EN67_LENED	Acetylglutamate kinase OS=Lentinula edodes OX=5353 GN=LENED_010616 PE=3 SV=1	1
95	tr|A0A1Q3DZ95|A0A1Q3DZ95_LENED	Phosphoglycerate mutase (2,3-diphosphoglycerate-independent) OS=Lentinula edodes OX=5353 GN=LENED_001820 PE=3 SV=1	2
96	tr|A0A1Q3EPG8|A0A1Q3EPG8_LENED	60S acidic ribosomal protein P0 OS=Lentinula edodes OX=5353 GN=LENED_011228 PE=3 SV=1	1
97	tr|A0A1Q3EEJ5|A0A1Q3EEJ5_LENED	Glucoamylase OS=Lentinula edodes OX=5353 GN=LENED_007456 PE=3 SV=1	3
98	tr|A0A1Q3EU67|A0A1Q3EU67_LENED	Mediator of RNA polymerase II transcription subunit 10 OS=Lentinula edodes OX=5353 GN=MED10 PE=3 SV=1	2
99	tr|A0A1Q3DWI3|A0A1Q3DWI3_LENED	Uncharacterized protein OS=Lentinula edodes OX=5353 GN=LENED_000734 PE=4 SV=1	2
99	tr|A0A1Q3DWE6|A0A1Q3DWE6_LENED	Uncharacterized protein OS=Lentinula edodes OX=5353 GN=LENED_000733 PE=4 SV=1	2
100	tr|A0A1Q3EC93|A0A1Q3EC93_LENED	Septin OS=Lentinula edodes OX=5353 GN=LENED_006644 PE=3 SV=1	1
101	tr|A0A1Q3EB42|A0A1Q3EB42_LENED	Heat shock protein OS=Lentinula edodes OX=5353 GN=LENED_006130 PE=3 SV=1	1
102	tr|A0A1Q3EM76|A0A1Q3EM76_LENED	5-oxoprolinase OS=Lentinula edodes OX=5353 GN=LENED_010355 PE=4 SV=1	1
103	tr|A0A1Q3E4S1|A0A1Q3E4S1_LENED	26S proteasome subunit P45 OS=Lentinula edodes OX=5353	1

		GN=LENED_003879 PE=3 SV=1	
104	tr|A0A6G9EM66|A0A6G9EM66_LENED	Glucose-6-phosphate isomerase (Fragment) OS=Lentinula edodes OX=5353 GN=pgi PE=2 SV=1	3
104	tr|A0A1Q3ETQ7|A0A1Q3ETQ7_LENED	Glucose-6-phosphate isomerase OS=Lentinula edodes OX=5353 GN=LENED_012869 PE=3 SV=1	3
105	tr|A0A1Q3E3S1|A0A1Q3E3S1_LENED	Endoplasmic reticulum chaperone BiP OS=Lentinula edodes OX=5353 GN=LENED_003354 PE=3 SV=1	4
106	tr|A0A1Q3EQ64|A0A1Q3EQ64_LENED	GTP binding protein OS=Lentinula edodes OX=5353 GN=LENED_011480 PE=3 SV=1	1
107	tr|A0A1Q3EQ38|A0A1Q3EQ38_LENED	6-phosphogluconate dehydrogenase, decarboxylating OS=Lentinula edodes OX=5353 GN=LENED_011465 PE=3 SV=1	3
108	tr|A0A1Q3E3G4|A0A1Q3E3G4_LENED	Ribosomal protein S5 OS=Lentinula edodes OX=5353 GN=LENED_003385 PE=3 SV=1	1
109	tr|A0A1Q3DUZ7|A0A1Q3DUZ7_LENED	Udp-n-acetylglucosamine diphosphorylase OS=Lentinula edodes OX=5353 GN=LENED_000210 PE=4 SV=1	1
110	tr|A0A1Q3E886|A0A1Q3E886_LENED	Proteasome subunit alpha type OS=Lentinula edodes OX=5353 GN=LENED_004987 PE=3 SV=1	1
110	tr|A0A1Q3E7S8|A0A1Q3E7S8_LENED	Proteasome subunit alpha type OS=Lentinula edodes OX=5353 GN=LENED_004988 PE=3 SV=1	1
111	tr|A0A1Q3EN90|A0A1Q3EN90_LENED	3-hydroxyacyl-[acyl-carrier-protein] dehydratase OS=Lentinula edodes OX=5353 GN=LENED_010754 PE=3 SV=1	1
112	tr|A0A1Q3DX58|A0A1Q3DX58_LENED	Ubiquitin activating enzyme OS=Lentinula edodes OX=5353 GN=LENED_000880 PE=3 SV=1	1

113	tr|A0A1Q3E6M9|A0A1Q3E6M9_LENED	Serine/threonine-protein phosphatase OS=Lentinula edodes OX=5353 GN=LENED_004573 PE=3 SV=1	1
114	tr|A0A1Q3ESU3|A0A1Q3ESU3_LENED	Rpt2-26s proteasome regulatory subunit OS=Lentinula edodes OX=5353 GN=LENED_012425 PE=3 SV=1	1
115	tr|A0A1Q3E1Y2|A0A1Q3E1Y2_LENED	Ribosomal protein L23a OS=Lentinula edodes OX=5353 GN=LENED_002619 PE=3 SV=1	1
116	tr|A0A1Q3E8J3|A0A1Q3E8J3_LENED	Plasma membrane ATPase OS=Lentinula edodes OX=5353 GN=LENED_005220 PE=3 SV=1	1
117	tr|A0A1Q3DZQ7|A0A1Q3DZQ7_LENED	Phosphoglucomutase OS=Lentinula edodes OX=5353 GN=pgm PE=2 SV=1	1
118	tr|A0A1Q3ER54|A0A1Q3ER54_LENED	Mitogen-activated protein kinase OS=Lentinula edodes OX=5353 GN=LENED_011865 PE=3 SV=1	1
119	tr|A0A1Q3EDG4|A0A1Q3EDG4_LENED	T-complex protein 1 OS=Lentinula edodes OX=5353 GN=LENED_007078 PE=3 SV=1	1
120	tr|A0A1Q3EAH4|A0A1Q3EAH4_LENED	40S ribosomal protein S5 OS=Lentinula edodes OX=5353 GN=LENED_005990 PE=3 SV=1	1
121	tr|A0A1Q3E8T0|A0A1Q3E8T0_LENED	Leucine aminopeptidase OS=Lentinula edodes OX=5353 GN=LENED_005212 PE=3 SV=1	2
122	tr|A0A1Q3E0B3|A0A1Q3E0B3_LENED	Eukaryotic initiation factor 4f subunit p130 OS=Lentinula edodes OX=5353 GN=LENED_002123 PE=3 SV=1	1
122	tr|A0A1Q3DZY9|A0A1Q3DZY9_LENED	Eukaryotic initiation factor 4f subunit p130 OS=Lentinula edodes OX=5353 GN=LENED_002119 PE=3 SV=1	1
123	tr|A0A1Q3ER32|A0A1Q3ER32_LENED	Malate dehydrogenase OS=Lentinula edodes OX=5353 GN=LENED_011840 PE=3 SV=1	1

124	tr|A0A1Q3DYM0|A0A1Q3DYM0_LENED	Proteasome subunit beta OS=Lentinula edodes OX=5353 GN=LENED_001597 PE=3 SV=1	1
125	tr|A0A1Q3EMU0|A0A1Q3EMU0_LENED	Succinate dehydrogenase [ubiquinone] flavoprotein subunit, mitochondrial OS=Lentinula edodes OX=5353 GN=LENED_010483 PE=3 SV=1	2
126	tr|A0A1Q3EQ01|A0A1Q3EQ01_LENED	White collar 1 protein OS=Lentinula edodes OX=5353 GN=LENED_011425 PE=4 SV=1	2
127	tr|A0A1Q3EHC7|A0A1Q3EHC7_LENED	Non-specific serine/threonine protein kinase OS=Lentinula edodes OX=5353 GN=LENED_008488 PE=3 SV=1	1
128	tr|A0A1Q3DYF7|A0A1Q3DYF7_LENED	Acyl-oxidase OS=Lentinula edodes OX=5353 GN=LENED_001431 PE=3 SV=1	1
128	tr|A0A1Q3DY69|A0A1Q3DY69_LENED	Acyl-oxidase OS=Lentinula edodes OX=5353 GN=LENED_001430 PE=3 SV=1	1
129	tr|A0A1Q3EAY4|A0A1Q3EAY4_LENED	Formate dehydrogenase OS=Lentinula edodes OX=5353 GN=LENED_006034 PE=3 SV=1	8
130	tr|A0A1Q3DZ81|A0A1Q3DZ81_LENED	Ribosomal protein l12 OS=Lentinula edodes OX=5353 GN=LENED_001827 PE=3 SV=1	2
131	tr|A0A1Q3ER04|A0A1Q3ER04_LENED	Proteasome subunit alpha type OS=Lentinula edodes OX=5353 GN=LENED_011810 PE=3 SV=1	1
132	tr|A0A1Q3EQA5|A0A1Q3EQA5_LENED	D-galacturonic acid reductase OS=Lentinula edodes OX=5353 GN=LENED_011536 PE=4 SV=1	1
133	tr|A0A1Q3E7Z4|A0A1Q3E7Z4_LENED	Aldo ketoreductase OS=Lentinula edodes OX=5353 GN=LENED_005000 PE=4 SV=1	1
133	tr|A0A1Q3E3M7|A0A1Q3E3M7_LENED	Aldo keto reductase OS=Lentinula edodes OX=5353 GN=LENED_003314	1

		PE=4 SV=1	
134	tr|A0A1Q3E4Z6|A0A1Q3E4Z6_LENED	26S proteasome subunit P45 OS=Lentinula edodes OX=5353 GN=LENED_003963 PE=3 SV=1	1
135	tr|A0A1Q3DVG4|A0A1Q3DVG4_LENED	60S ribosomal protein L9 OS=Lentinula edodes OX=5353 GN=LENED_000342 PE=3 SV=1	1
136	tr|A0A1Q3EHJ0|A0A1Q3EHJ0_LENED	UDP-glucose 6-dehydrogenase OS=Lentinula edodes OX=5353 GN=LENED_008601 PE=3 SV=1	1
137	tr|A0A1Q3DXK3|A0A1Q3DXK3_LENED	S-(hydroxymethyl)glutathione dehydrogenase OS=Lentinula edodes OX=5353 GN=LENED_000926 PE=3 SV=1	3
138	tr|A0A1Q3EQA3|A0A1Q3EQA3_LENED	Small nuclear ribonucleoprotein Sm D2 OS=Lentinula edodes OX=5353 GN=LENED_011540 PE=3 SV=1	1
139	tr|A0A1Q3EH22|A0A1Q3EH22_LENED	Voltage-dependent ion-selective channel OS=Lentinula edodes OX=5353 GN=LENED_008414 PE=4 SV=1	2
140	tr|A0A1Q3EDA2|A0A1Q3EDA2_LENED	Small nuclear ribonucleoprotein Sm D3 OS=Lentinula edodes OX=5353 GN=LENED_007022 PE=3 SV=1	1
141	tr|A0A1Q3DWK9|A0A1Q3DWK9_LENED	Actin 1 OS=Lentinula edodes OX=5353 GN=LENED_000851 PE=3 SV=1	7
142	tr|A0A1Q3E652|A0A1Q3E652_LENED	Peptide chain release factor eRF aRF subunit 1 OS=Lentinula edodes OX=5353 GN=LENED_004158 PE=3 SV=1	1
143	tr|A0A1Q3E2E5|A0A1Q3E2E5_LENED	Wd repeat-containing protein 24 OS=Lentinula edodes OX=5353 GN=LENED_003009 PE=3 SV=1	1
143	tr|A0A1Q3EPC9|A0A1Q3EPC9_LENED	40S ribosomal protein S14 OS=Lentinula edodes OX=5353 GN=LENED_011178 PE=3 SV=1	1

144	tr|A0A1Q3DUU2|A0A1Q3DUU2_LENED	60S acidic ribosomal protein P1 OS=Lentinula edodes OX=5353 GN=LENED_000146 PE=3 SV=1	1
145	tr|A4UX71|A4UX71_LENED	CIPB OS=Lentinula edodes OX=5353 GN=cipB PE=2 SV=1	1
146	tr|A0A1Q3EMC8|A0A1Q3EMC8_LENED	Vacuolar protein 8 OS=Lentinula edodes OX=5353 GN=LENED_010392 PE=3 SV=1	1
147	tr|A0A1Q3EJI7|A0A1Q3EJI7_LENED	Superoxide dismutase OS=Lentinula edodes OX=5353 GN=LENED_009318 PE=3 SV=1	3
148	tr|A0A1Q3EAG0|A0A1Q3EAG0_LENED	Carboxylic ester hydrolase OS=Lentinula edodes OX=5353 GN=LENED_005967 PE=3 SV=1	4
149	tr|A0A1Q3E9V7|A0A1Q3E9V7_LENED	Ca2+-binding actin-bundling protein OS=Lentinula edodes OX=5353 GN=LENED_005773 PE=4 SV=1	1
150	tr|A0A1Q3E3A9|A0A1Q3E3A9_LENED	40S ribosomal protein S6 OS=Lentinula edodes OX=5353 GN=LENED_003322 PE=3 SV=1	1
151	tr|A0A1Q3DXW7|A0A1Q3DXW7_LENED	L-arabinitol 4-dehydrogenase OS=Lentinula edodes OX=5353 GN=LENED_001158 PE=3 SV=1	1
152	tr|Q870G4|Q870G4_LENED	Rab small monomeric gtpase OS=Lentinula edodes OX=5353 GN=Rab7 PE=2 SV=1	1
153	tr|A0A1Q3ERE4|A0A1Q3ERE4_LENED	Cytochrome c oxidase subunit OS=Lentinula edodes OX=5353 GN=LENED_011974 PE=3 SV=1	1
154	tr|A0A1Q3ER18|A0A1Q3ER18_LENED	S-adenosylmethionine synthase OS=Lentinula edodes OX=5353 GN=LENED_011822 PE=3 SV=1	1
155	tr|A0A1Q3EQ23|A0A1Q3EQ23_LENED	Proteasome subunit beta OS=Lentinula edodes OX=5353 GN=LENED_011458 PE=3 SV=1	1
156	tr|A0A1Q3EQ02|A0A1Q3EQ02_LENED	60s ribosomal protein l31 OS=Lentinula edodes OX=5353	1

		GN=LENED_011430 PE=3 SV=1	
157	tr|A0A1Q3EMR3|A0A1Q3EMR3_LENED	NADH:flavin oxidoreductase NADH oxidase OS=Lentinula edodes OX=5353 GN=LENED_010567 PE=4 SV=1	1
158	tr|A0A1Q3EM47|A0A1Q3EM47_LENED	Transformer-2-beta isoform 3 OS=Lentinula edodes OX=5353 GN=LENED_010307 PE=4 SV=1	1
158	tr|A0A1Q3EM26|A0A1Q3EM26_LENED	Transformer-2-beta isoform 3 OS=Lentinula edodes OX=5353 GN=LENED_010308 PE=4 SV=1	1
159	tr|A0A1Q3EL39|A0A1Q3EL39_LENED	Protein OS=Lentinula edodes OX=5353 GN=LENED_009926 PE=3 SV=1	1
160	tr|A0A1Q3EK74|A0A1Q3EK74_LENED	Cleavage and polyadenylation specificity factor subunit 5 OS=Lentinula edodes OX=5353 GN=LENED_009596 PE=3 SV=1	1
161	tr|A0A1Q3EJW0|A0A1Q3EJW0_LENED	Uridylate kinase OS=Lentinula edodes OX=5353 GN=LENED_009505 PE=3 SV=1	1
161	sp|O59845|KCY_LENED	UMP-CMP kinase OS=Lentinula edodes OX=5353 GN=uck1 PE=1 SV=1	1
162	tr|A0A1Q3EJ63|A0A1Q3EJ63_LENED	Peptidyl-prolyl cis-trans isomerase OS=Lentinula edodes OX=5353 GN=LENED_009208 PE=3 SV=1	2
162	tr|A0A1Q3EJ55|A0A1Q3EJ55_LENED	Peptidyl-prolyl cis-trans isomerase OS=Lentinula edodes OX=5353 GN=LENED_009207 PE=3 SV=1	2
163	tr|A0A1Q3EJ00|A0A1Q3EJ00_LENED	Actin-related protein 2 OS=Lentinula edodes OX=5353 GN=LENED_009147 PE=3 SV=1	1
164	tr|A0A1Q3EH92|A0A1Q3EH92_LENED	Coatomer subunit gamma OS=Lentinula edodes OX=5353 GN=LENED_008489 PE=3 SV=1	1
165	tr|A0A1Q3EH82|A0A1Q3EH82_LENED	ARID domain-containing protein OS=Lentinula edodes OX=5353	1

		GN=LENED_008479 PE=4 SV=1	
166	tr|A0A1Q3EFL8|A0A1Q3EFL8_LENED	Peptide-methionine (R)-S-oxide reductase OS=Lentinula edodes OX=5353 GN=LENED_007876 PE=3 SV=1	1
167	tr|A0A1Q3EEZ2|A0A1Q3EEZ2_LENED	Adhesion regulating molecule OS=Lentinula edodes OX=5353 GN=LENED_007662 PE=4 SV=1	1
168	tr|A0A1Q3EEF3|A0A1Q3EEF3_LENED	Glucosamine-6-phosphate isomerase OS=Lentinula edodes OX=5353 GN=LENED_007454 PE=3 SV=1	1
169	tr|A0A1Q3EDL7|A0A1Q3EDL7_LENED	Peroxisomal hydratase-dehydrogenase-epimerase OS=Lentinula edodes OX=5353 GN=LENED_007065 PE=4 SV=1	1
170	tr|A0A1Q3EDG2|A0A1Q3EDG2_LENED	Obg-like ATPase 1 OS=Lentinula edodes OX=5353 GN=LENED_007087 PE=3 SV=1	1
171	tr|A0A1Q3EDB8|A0A1Q3EDB8_LENED	Ribosomal protein S12 S23 OS=Lentinula edodes OX=5353 GN=LENED_007048 PE=3 SV=1	2
172	tr|A0A1Q3ED50|A0A1Q3ED50_LENED	Nascent polypeptide-associated complex subunit beta OS=Lentinula edodes OX=5353 GN=LENED_006972 PE=3 SV=1	1
173	tr|A0A1Q3EAI4|A0A1Q3EAI4_LENED	UTP--glucose-1-phosphate uridylyltransferase OS=Lentinula edodes OX=5353 GN=LENED_006000 PE=3 SV=1	1
174	tr|A0A1Q3E7L7|A0A1Q3E7L7_LENED	Glycolipid transfer protein OS=Lentinula edodes OX=5353 GN=LENED_004935 PE=4 SV=1	1
175	tr|A0A1Q3E7H5|A0A1Q3E7H5_LENED	Glutamyl-tRNA synthetase OS=Lentinula edodes OX=5353 GN=LENED_004885 PE=3 SV=1	1
176	tr|A0A1Q3E4U8|A0A1Q3E4U8_LENED	Glycoside hydrolase family 17 protein OS=Lentinula edodes OX=5353	1

		GN=LENED_003912 PE=4 SV=1	
177	tr|A0A1Q3E3S6|A0A1Q3E3S6_LENED	3-isopropylmalate dehydratase OS=Lentinula edodes OX=5353 GN=LENED_003504 PE=3 SV=1	1
178	tr|A0A1Q3E2Z3|A0A1Q3E2Z3_LENED	Inosine-5'-monophosphate dehydrogenase OS=Lentinula edodes OX=5353 GN=LENED_003073 PE=3 SV=1	1
179	tr|A0A1Q3E0M7|A0A1Q3E0M7_LENED	60S ribosomal protein L33 OS=Lentinula edodes OX=5353 GN=LENED_002087 PE=3 SV=1	1
180	tr|A0A1Q3E009|A0A1Q3E009_LENED	60S ribosomal protein L23 OS=Lentinula edodes OX=5353 GN=LENED_002115 PE=3 SV=1	1
181	tr|A0A1Q3DZX9|A0A1Q3DZX9_LENED	Protein phosphatase PP2A regulatory subunit B OS=Lentinula edodes OX=5353 GN=LENED_001982 PE=3 SV=1	1
182	tr|A0A1Q3DZ25|A0A1Q3DZ25_LENED	Phosphoglycerate mutase-like protein OS=Lentinula edodes OX=5353 GN=LENED_001498 PE=3 SV=1	1
182	tr|A0A1Q3DYD4|A0A1Q3DYD4_LENED	Phosphoglycerate mutase-like protein OS=Lentinula edodes OX=5353 GN=LENED_001497 PE=3 SV=1	1
183	tr|A0A1Q3DYI3|A0A1Q3DYI3_LENED	Chromosome condensation complex protein OS=Lentinula edodes OX=5353 GN=LENED_001291 PE=4 SV=1	1
184	tr|A0A1Q3DX90|A0A1Q3DX90_LENED	DEAD-domain-containing protein OS=Lentinula edodes OX=5353 GN=LENED_001096 PE=3 SV=1	1
185	tr|A0A1Q3DW13|A0A1Q3DW13_LENED	Proteasome subunit alpha type OS=Lentinula edodes OX=5353 GN=LENED_000587 PE=3 SV=1	1
186	RRRRRtr|A0A1Q3E7R2|A0A1Q3E7R2_LENED	REVERSED Sphingomyelin phosphodiesterase OS=Lentinula edodes OX=5353 GN=LENED_005001	1

		PE=3 SV=1	
187	tr|A0A1Q3EGV5|A0A1Q3EGV5_LENED	40S ribosomal protein S20 OS=Lentinula edodes OX=5353 GN=LENED_008386 PE=3 SV=1	1
188	tr|A0A1Q3ENN8|A0A1Q3ENN8_LENED	Serine-type endopeptidase OS=Lentinula edodes OX=5353 GN=LENED_010921 PE=3 SV=1	2
189	tr|A0A1Q3EHG3|A0A1Q3EHG3_LENED	Aspartate aminotransferase OS=Lentinula edodes OX=5353 GN=LENED_008562 PE=3 SV=1	3
190	tr|A0A1Q3E1X4|A0A1Q3E1X4_LENED	Peptidase S28 OS=Lentinula edodes OX=5353 GN=LENED_002692 PE=4 SV=1	1
191	tr|A0A1Q3E354|A0A1Q3E354_LENED	Centromere-associated protein E OS=Lentinula edodes OX=5353 GN=LENED_003198 PE=4 SV=1	1
191	tr|A0A1Q3E2X6|A0A1Q3E2X6_LENED	Centromere-associated protein E OS=Lentinula edodes OX=5353 GN=LENED_003197 PE=4 SV=1	1
192	tr|A0A1Q3EJP2|A0A1Q3EJP2_LENED	Myosin regulatory light chain cdc4 OS=Lentinula edodes OX=5353 GN=LENED_009409 PE=4 SV=1	1
193	tr|A0A1Q3EAG9|A0A1Q3EAG9_LENED	60s ribosomal protein l20 OS=Lentinula edodes OX=5353 GN=LENED_005978 PE=3 SV=1	1
194	tr|A0A1Q3DYE7|A0A1Q3DYE7_LENED	60S ribosomal protein L17 L23 OS=Lentinula edodes OX=5353 GN=LENED_001436 PE=3 SV=1	1
195	tr|A0A1Q3EM03|A0A1Q3EM03_LENED	Arp2/3 complex 34 kDa subunit OS=Lentinula edodes OX=5353 GN=LENED_010200 PE=3 SV=1	1
196	tr|A0A1Q3EEY4|A0A1Q3EEY4_LENED	40s ribosomal protein s19 OS=Lentinula edodes OX=5353 GN=LENED_007639 PE=3 SV=1	1
197	tr|A0A1Q3ESP3|A0A1Q3ESP3_LENED	BTB domain-containing protein OS=Lentinula edodes OX=5353 GN=LENED_012366 PE=4 SV=1	1

197	tr|A0A1Q3ESF4|A0A1Q3ESF4_LENED	BTB domain-containing protein OS=Lentinula edodes OX=5353 GN=LENED_012367 PE=4 SV=1	1
198	tr|A0A1Q3E868|A0A1Q3E868_LENED	Glutathione S-transferase C-terminal-like protein OS=Lentinula edodes OX=5353 GN=LENED_005172 PE=3 SV=1	1
199	tr|A0A1Q3E6K9|A0A1Q3E6K9_LENED	Glutamate dehydrogenase OS=Lentinula edodes OX=5353 GN=LENED_004547 PE=3 SV=1	2
200	tr|A0A1Q3E2B0|A0A1Q3E2B0_LENED	Small COPII coat GTPase SAR1 OS=Lentinula edodes OX=5353 GN=LENED_002978 PE=3 SV=1	1
201	tr|A0A1Q3E746|A0A1Q3E746_LENED	Extracellular metalloproteinase OS=Lentinula edodes OX=5353 GN=LENED_004682 PE=3 SV=1	1
202	tr|A0A1Q3EF98|A0A1Q3EF98_LENED	Cytoplasm protein OS=Lentinula edodes OX=5353 GN=LENED_007772 PE=4 SV=1	1
203	tr|C5NN47|C5NN47_LENED	Protein disulfide-isomerase OS=Lentinula edodes OX=5353 GN=pdi1 PE=2 SV=1	1
203	tr|A0A1Q3EDT5|A0A1Q3EDT5_LENED	Protein disulfide-isomerase OS=Lentinula edodes OX=5353 GN=LENED_007225 PE=3 SV=1	1
204	tr|A0A1Q3DW02|A0A1Q3DW02_LENED	DEAD-domain-containing protein OS=Lentinula edodes OX=5353 GN=LENED_000528 PE=3 SV=1	1
205	tr|A0A1Q3EIY5|A0A1Q3EIY5_LENED	Glutamate--ammonia ligase OS=Lentinula edodes OX=5353 GN=LENED_009124 PE=3 SV=1	1
206	tr|A0A1Q3ER08|A0A1Q3ER08_LENED	Small nuclear ribonucleoprotein Sm D1 OS=Lentinula edodes OX=5353 GN=LENED_011791 PE=3 SV=1	1
207	tr|A0A1Q3E3Y2|A0A1Q3E3Y2_LENED	Pkinase-domain-containing protein OS=Lentinula edodes OX=5353 GN=LENED_003564 PE=3 SV=1	1

208	tr|A0A1Q3E5D9|A0A1Q3E5D9_LENED	Short-chain dehydrogenase reductase sdr OS=Lentinula edodes OX=5353 GN=LENED_004130 PE=4 SV=1	1
209	tr|A0A1Q3EQ42|A0A1Q3EQ42_LENED	Nuclear and cytoplasmic polyadenylated rna-binding protein pub1 OS=Lentinula edodes OX=5353 GN=LENED_011471 PE=4 SV=1	1
210	tr|A0A1Q3EDT6|A0A1Q3EDT6_LENED	Uncharacterized protein OS=Lentinula edodes OX=5353 GN=LENED_007212 PE=4 SV=1	1
211	tr|A0A1Q3EJP1|A0A1Q3EJP1_LENED	Amp-ligase OS=Lentinula edodes OX=5353 GN=LENED_009404 PE=4 SV=1	1
212	tr|A0A1Q3EA66|A0A1Q3EA66_LENED	Dipeptidyl peptidase iii OS=Lentinula edodes OX=5353 GN=LENED_005884 PE=4 SV=1	1
213	tr|A0A1Q3DW44|A0A1Q3DW44_LENED	E set domain-containing protein OS=Lentinula edodes OX=5353 GN=LENED_000596 PE=3 SV=1	1
214	tr|A0A1Q3DUI6|A0A1Q3DUI6_LENED	60S ribosomal protein L8 OS=Lentinula edodes OX=5353 GN=LENED_000020 PE=3 SV=1	2
214	tr|A0A1Q3E0J4|A0A1Q3E0J4_LENED	60S ribosomal protein L8 OS=Lentinula edodes OX=5353 GN=LENED_002174 PE=3 SV=1	2
214	tr|A0A1Q3E059|A0A1Q3E059_LENED	60S ribosomal protein L8 OS=Lentinula edodes OX=5353 GN=LENED_002175 PE=3 SV=1	2
215	tr|A0A1Q3ETE1|A0A1Q3ETE1_LENED	Mitochondrial outer membrane protein IML2 OS=Lentinula edodes OX=5353 GN=LENED_012622 PE=4 SV=1	1
216	tr|A0A1Q3ECH7|A0A1Q3ECH7_LENED	Uncharacterized protein OS=Lentinula edodes OX=5353 GN=LENED_006734 PE=4 SV=1	1
217	tr|A0A1Q3ETP4|A0A1Q3ETP4_LENED	Sm-like ribonucleo protein OS=Lentinula edodes OX=5353	1

		GN=LENED_012841 PE=4 SV=1	
218	tr|A0A1Q3EIX0|A0A1Q3EIX0_LENED	Isocitrate dehydrogenase [NAD] subunit, mitochondrial OS=Lentinula edodes OX=5353 GN=LENED_009096 PE=3 SV=1	1
220	tr|A0A1Q3EAK5|A0A1Q3EAK5_LENED	Isoleucyl-tRNA synthetase OS=Lentinula edodes OX=5353 GN=LENED_006037 PE=3 SV=1	1
221	tr|A0A1Q3EKF9|A0A1Q3EKF9_LENED	DUF427-domain-containing protein OS=Lentinula edodes OX=5353 GN=LENED_009717 PE=4 SV=1	1
223	tr|A0A1Q3EK11|A0A1Q3EK11_LENED	Chorismate synthase OS=Lentinula edodes OX=5353 GN=LENED_009444 PE=3 SV=1	1
224	tr|A0A1Q3E8E6|A0A1Q3E8E6_LENED	Phosphotransferase OS=Lentinula edodes OX=5353 GN=LENED_005054 PE=3 SV=1	1
225	tr|A0A1Q3E4B5|A0A1Q3E4B5_LENED	Autophagy-related protein OS=Lentinula edodes OX=5353 GN=LENED_003559 PE=3 SV=1	1
225	tr|A0A1Q3E3W7|A0A1Q3E3W7_LENED	Autophagy-related protein OS=Lentinula edodes OX=5353 GN=LENED_003558 PE=3 SV=1	1
226	tr|A0A1Q3EF43|A0A1Q3EF43_LENED	Mov34-domain-containing protein OS=Lentinula edodes OX=5353 GN=LENED_007716 PE=3 SV=1	1
229	tr|A0A1Q3EM21|A0A1Q3EM21_LENED	Udp-xylose synthase OS=Lentinula edodes OX=5353 GN=LENED_010301 PE=4 SV=1	1
234	tr|A0A1Q3ERQ2|A0A1Q3ERQ2_LENED	1,3-beta-glucanosyltransferase OS=Lentinula edodes OX=5353 GN=LENED_012085 PE=3 SV=1	1
235	tr|A0A1Q3EKA9|A0A1Q3EKA9_LENED	NAD-aldehyde dehydrogenase OS=Lentinula edodes OX=5353 GN=LENED_009666 PE=3 SV=1	2
237	RRRRRtr|A0A1Q3ET10|A0A1Q3ET10_LENED	REVERSED Kinesin-like protein OS=Lentinula edodes OX=5353	1

		GN=LENED_012572 PE=3 SV=1	
248	tr|A0A1Q3EHR2|A0A1Q3EHR2_LENED	PKS_ER domain-containing protein OS=Lentinula edodes OX=5353 GN=LENED_008624 PE=4 SV=1	2
250	tr|A0A1Q3E2X8|A0A1Q3E2X8_LENED	Proteasome subunit beta OS=Lentinula edodes OX=5353 GN=LENED_002970 PE=3 SV=1	1
259	tr|A0A1Q3DZQ1|A0A1Q3DZQ1_LENED	Uncharacterized protein OS=Lentinula edodes OX=5353 GN=LENED_001902 PE=4 SV=1	1
262	tr|A0A1Q3DV72|A0A1Q3DV72_LENED	T-complex protein 1 subunit eta OS=Lentinula edodes OX=5353 GN=LENED_000311 PE=3 SV=1	1
266	tr|A0A1Q3DZ42|A0A1Q3DZ42_LENED	Aldehyde dehydrogenase OS=Lentinula edodes OX=5353 GN=LENED_001782 PE=3 SV=1	1
269	tr|A0A1Q3EAJ7|A0A1Q3EAJ7_LENED	Delta-aminolevulinic acid dehydratase OS=Lentinula edodes OX=5353 GN=LENED_006009 PE=3 SV=1	1
272	tr|A0A1Q3ETH4|A0A1Q3ETH4_LENED	Rab-type small GTP-binding protein OS=Lentinula edodes OX=5353 GN=LENED_012761 PE=4 SV=1	1
332	tr|A0A1Q3EQ64|A0A1Q3EQ64_LENED	GTP binding protein OS=Lentinula edodes OX=5353 GN=LENED_011480 PE=3 SV=1	1

Peptides: 95% confidence of identification, OS: organism name, OX: organism identifier GN: Gene name, PE: Protein existence and SV: Sequence version.
